# Comparing Statistical Methods for Constructing Large Scale Gene Networks

**DOI:** 10.1371/journal.pone.0029348

**Published:** 2012-01-17

**Authors:** Jeffrey D. Allen, Yang Xie, Min Chen, Luc Girard, Guanghua Xiao

**Affiliations:** 1 Quantitative Biomedical Research Center, University of Texas Southwestern Medical Center, Dallas, Texas, United States of America; 2 Harold C. Simmons Comprehensive Cancer Center, University of Texas Southwestern Medical Center, Dallas, Texas, United States of America; 3 Department of Pharmacology, University of Texas Southwestern Medical Center, Dallas, Texas, United States of America; 4 Hamon Center for Therapeutic Oncology Research, University of Texas Southwestern Medical Center, Dallas, Texas, United States of America; Umeå University, Sweden

## Abstract

The gene regulatory network (GRN) reveals the regulatory relationships among genes and can provide a systematic understanding of molecular mechanisms underlying biological processes. The importance of computer simulations in understanding cellular processes is now widely accepted; a variety of algorithms have been developed to study these biological networks. The goal of this study is to provide a comprehensive evaluation and a practical guide to aid in choosing statistical methods for constructing large scale GRNs. Using both simulation studies and a real application in E. coli data, we compare different methods in terms of sensitivity and specificity in identifying the true connections and the hub genes, the ease of use, and computational speed. Our results show that these algorithms performed reasonably well, and each method has its own advantages: (1) GeneNet, WGCNA (Weighted Correlation Network Analysis), and ARACNE (Algorithm for the Reconstruction of Accurate Cellular Networks) performed well in constructing the global network structure; (2) GeneNet and SPACE (Sparse PArtial Correlation Estimation) performed well in identifying a few connections with high specificity.

## Introduction

Gene regulatory networks describe interactions among genes and how they work together to form modules to carry out cell functions. GRNs provide a systematic understanding of molecular mechanisms underlying biological processes [1]–[6]; the visualization of direct dependencies facilitates systematic interpretation and comprehension of the relationships among genes. In the GRN, genes that interact with many other genes are called hub genes. The hub genes are likely to be drivers of the disease status due to their key positions in the GRNs. Recently, analysis of hub genes has shown to be a promising approach in identifying key tumorigenic genes [7]–[10].

Gene expression microarrays monitor the transcription activities of thousands of genes simultaneously, which provides great opportunities to explore large scale regulatory networks. Genetic dependency graphs can and have been constructed through a variety of approaches. Four categories of statistical methods have been proposed to construct the GRN from gene expression microarray data: (1) Probabilistic networks-based approaches, mainly Bayesian networks (BN), (2) correlation-based methods, (3) partial-correlation-based methods, and (4) Information-theory-based methods. We give the detailed description of each type in the Methods section.

In this paper, we compared several statistical methods for constructing GRNs. Our goal is to provide a comprehensive evaluation and a practical guide to help investigators choose between different methods for constructing large scale GRNs. The main contributions of this paper include: (1) The performance on constructing large scale GRNs is compared with a wide range of sample sizes and numbers of genes in the network; (2) The performance of identifying correct hub genes, which are likely to be the disease driver genes, is compared among different methods; (3) In addition to previously reviewed methods (Bayesian Networks [11] and GeneNet [12]), three recently developed programs (Sparse PArtial Correlation Estimation (SPACE) [13], Weighted Correlation Network Analysis (WGCNA) [14], and ARACNE (Algorithm for the Reconstruction of Accurate Cellular Networks) [15], [16]) are included in the comparison.

In this study, we are interested not only in comparing the performances of various network construction methods, but also in how the number of microarray experiments affects the accuracy of the constructed network. In the simulation study, we simulated different numbers of microarray experiments for each simulation setting to study the effect of sample size on the performance of various methods.

## Methods

### Statistical Methods

Here we give a brief summary of four categories of GRN construction approaches; the detailed methodology for each approach has been described in other papers [11]–[13], [17]–[19]. For fair comparisons, the default parameters were used for each algorithm without additional tuning. We have provided Sweave documents to accompany this study as shown in Sweave S1; Sweave is a literate programming framework which combines the source code (in R) and documentation (in LaTeX) in one file, to facilitate the reproduction of our results.

Correlation-based methods [14], [17], [20] are the most straightforward way to explore the gene co-expression network. They usually define a gene co-expression similarity matrix 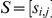, where 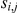 is the pair-wise transcription correlation coefficients between genes 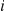 and 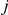, and 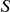 is the correlation matrix. Then either a hard [21] or soft threshold [14], [17] is applied to 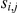 to determine the biological meaningfulness of the connections. These co-expression-based methods have been used in several studies and have shown their usefulness in interpreting biological results and identifying important gene modules [6], [20], [22]–[24]. WGCNA is a relatively new statistical approach based on correlations and has been used to identify several novel disease-related genes. Therefore, we will use WGCNA as a representative method for the correlation-based approach. The WGCNA R package implements both weighted and unweighted correlation networks and identifies modules/sub-networks using hierarchical clustering approaches. Aside from the functions for network construction and module/sub-network identification, the R package also provides functions for calculating topological properties and network visualization [14]. Furthermore, the WGCNA R package includes interfaces with several commonly used bioinformatics tools for network visualization (e.g. VisANT [25] and Cytoscape [26]) and enrichment analysis (e.g. DAVID [27]). The WGCNA method has been successfully applied in several studies [28]–[31].

Partial-correlation-based methods are based on Gaussian graphic model [32] theory. They infer the conditional dependency by the non-zero entries in the concentration matrix, 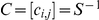, also called the precision matrix, which is the inverse of covariance matrix. The zero entries 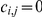 in the concentration matrix imply conditional independency between the expression levels of gene 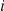 and 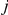 given the expression of all other genes; in other words, two genes do not interact directly with each other. Two recently published methods: SPACE [13] and GeneNet [12] will be used to represent partial-correlation-based methods. GeneNet uses Moore-Penrose pseudoinverse [33] and bootstrap methods to obtain a shrunk estimate of the concentration matrix. The SPACE algorithm converts the concentration matrix estimation problem to a regression problem and optimizes the results with a symmetric constraint and an 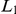 penalization. Therefore, SPACE tends to get more globally optimized results when compared to GeneNet. In this study, the partial correlation referred to first order partial correlation.

Information-theory-based methods, such as ARACNE, use mutual information (MI) to determine the dependency among the genes and then remove indirect interactions using data processing inequality (DPI). ARACNE has been successfully applied to construct gene regulatory networks in the context of specific cellular types, and demonstrated good performance. Since the calculation of mutual information does not assume a monotonic relationship, an advantage of information-theory-based methods is the ability to identify the non-linear or irregular dependencies, which will be missed by Pearson correlation. Therefore, the information-theory-based methods could out-perform correlation-based methods if the gene network contains many non-monotonic dependencies.

Probabilistic networks take a wholly different approach by attempting to search through the space of all the possible topological network arrangements given certain constraints. BNs are based on a probabilistic graphical model that represents a set of variables and their probabilistic independencies and are applicable to many areas in science and technology [34]. The probabilistic nature of BNs allows them to handle noise inherent in both biological processes and microarray experiments. The gene expression profiles could provide a complete joint distribution of gene expression levels, while a BN expands the joint probability in terms of simpler conditional probabilities. In our study, we have applied BNArray [35], B-course [36], BNT [37], and Werhli's implementation of BN [11]. BNArray does not run appropriately in our computation settings. Werhli's implementation of BN uniformly outperformed others, which is probably due to the fact that Werhli's method is specifically developed for constructing GRNs, while BNT and B-course are designed for general use. So, in this study, Werhli's BN implementation was used to represent the best performance of BN methods. The statistical methods used in this study, as well as their inference categories and implementation platforms are summarized in Table 1.

**Table 1:** Method Comparison.

Statistical method	Category	Implementation
BNArray [35]	BN	R
B-Course [36]	BN	C
BNT [37]	BN	Matlab
Werhli's BN Implementation [11]	BN	Matlab
SPACE [13]	Partial Correlation	C,R
GeneNet [12]	Partial Correlation	R
WGCNA [14]	Correlation	C, R
Aracne [15], [16]	Information Theory	C++, Java

Statistical methods for constructing GRN compared in this study.

### Performance Metrics

Some types of networks require that connections be acyclic. Other types of networks may differ on whether or not the connections are directed (causal). BN methods are acyclic and impose a direction on each edge; for the purposes of this study, these directions are ignored. The GRNs without directions are also called gene association networks.

We used the receiver operating characteristic (ROC) curves to study the sensitivity and specificity of each algorithm to minimize the influence of any default thresholds or cutoff values, and the area under the curve (AUC) was used to quantify the performance of each method. Clearly, the larger the area under the curve, the better the algorithm performed. ROC curves were determined by changing the threshold for connection strength (for example, connection strength for the SPACE algorithm refers to the absolute values of the estimated partial correlations). Two genes with a connection strength higher than the threshold were deemed to be connected.

The AUC measures the performance of the algorithm across all sensitivity and specificity ranges. In practice, biological researchers are more interested in a small subset of that performance curve – specifically, the part of the curve with high specificity. In order to calculate a metric more relevant to this application, we can use a partial AUC. This metric calculates the AUC for the ROC curve only where specificity is greater than some threshold. In this study we use the region in which specificity is greater than 99.5% (i.e. the false positive rate is less than 0.005) to calculate the pAUC. We also examined the pAUC with false positive rate less than 0.05 and obtained very similar results. The global AUC is more intuitive in measuring the overall predictive performance, while the pAUC provides a useful metric in measuring predictive performance at high specificity, which is usually the focus for biological researchers. In this study, we will use both AUC and pAUC to comprehensively evaluate the performance of network construction.

Another aspect of performance evaluated in this study was the detection of “hub genes”. A hub gene is a highly connected gene in a network; such genes are often of biological interest because of their critical involvement in regulatory pathways or sub-networks and these genes often incur a substantial effect on the pathways as a whole. Thus, we also evaluate each method's ability to identify hub genes in each network using gene “connectivity.” Gene connectivity (or the degree of a gene) is a way of stating how connected a gene is within a network. Some methods produced adjacency matrices with entirely non-zero entries. Such networks are “complete” and all nodes in each graph have the same “degree,” in that each gene is connected to each other gene. Due to this we define each gene's connectivity score in a given network by computing the sum of the weights of all connections associated with that gene. This score can then be compared to the actual connectivity score of a gene in the true network. Second, we also calculate the sensitivity and specificity of each method's connectivity predictions. To do this, we first classify each gene as a hub gene or not based on the true network using some cutoff. We then utilize an ROC curve which discloses the threshold-independent performance of a method on a given network and quantify this curve using the AUC.

### Hardware

Computational equipment used in this study included a Dell T300 server with 16 GB 667 MHz, DDR2 RAM, Dual Core Intel Xeon E 3113 (3.0 GHz) CPU and the Windows Server 2008 Operating System; and a RedHat Enterprise Linux server with 48 GB of RAM and two Intel Xeon X5650 (2.66 GHz) CPUs.

## Results

### Simulation Studies

In the simulation studies, the network structures were simulated based on the real protein-protein interaction networks [38], [39], with an approximately scale free topology. The strengths of dependencies were randomly simulated from a normal distribution N(0.5,0.2) with the sign (positive or negative regulation) simulated from a binomial distribution with probability 0.5. Specifically, 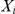, the expression of gene 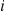, was simulated from conditional normal distributions 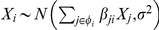, where 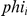 refers to a set of genes that regulate gene 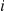 based on the simulated network structure, 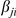 is the strength of dependency of gene 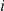 on gene 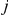, and 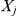, is the expression level of gene 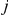 which is true for most microarray studies [40].

**Figure 1:**
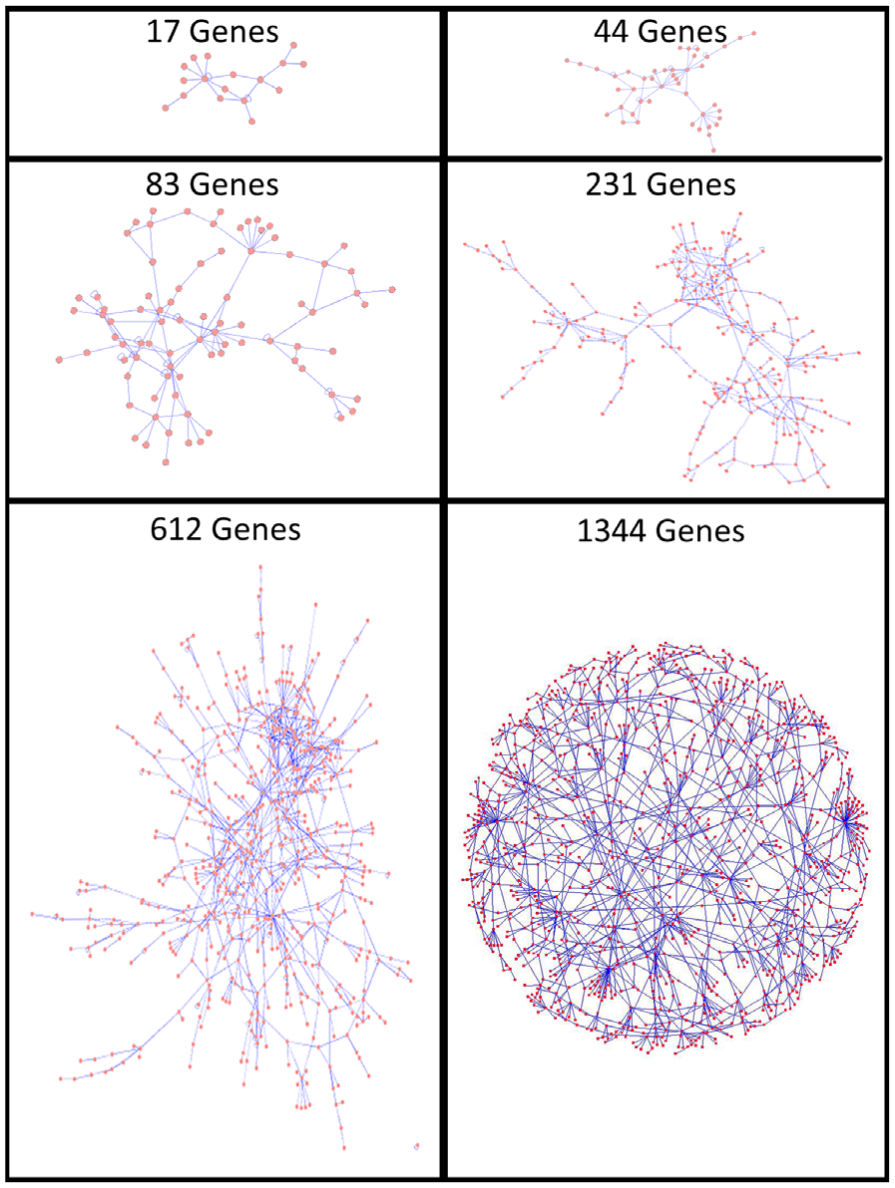
Diagram depicting the network structures of each of the six network sizes used in this study.

In each study, the datasets were simulated across two independent variables: (1) network size, and (2) numbers of samples. The number of genes represented in the networks varied over a wide range. Simulated networks had one of six sizes: 17 genes with 20 connections, 44 genes with 57 connections, 83 genes with 114 connections, 231 genes with 311 connections, 612 genes with 911 connections, or 1344 genes with 1511 connections as shown in Figure 1. We base these networks off of real protein-protein interaction networks and, to construct networks of different sizes, vary the number of references required to support each connection. The other variable in the simulated data was the number of samples (microarrays) in a dataset. Datasets had 20, 50, 100, 200, 500, or 1,000 simulated microarray samples. Obviously, the intuition is that, as the number of samples increases, the algorithms would be able to perform better [41]. Datasets were generated for all combinations of these variables, producing 36 total data sets for each simulation study.

### Network Construction Performance

**Figure 2:**
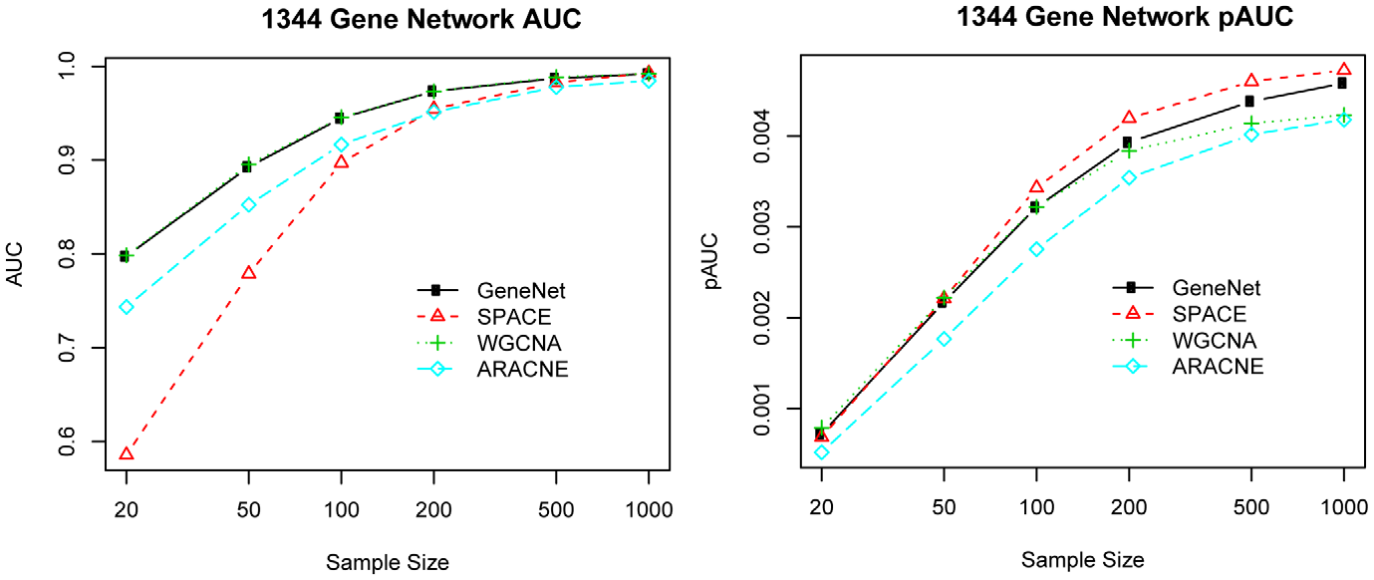
The AUCs and pAUCs for 1344-gene network in simulation 1. Left: The area under the curve using various network construction methods across various sample sizes on a network with 1344 genes; Right: The partial area under the curve for FPR
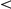
0.005 for various methods.

We first calculated the ROC curves for each combination of network size, sample size, and construction algorithm; the results of the 17-gene network are shown in Supplementary Figure S1. We use both AUC and pAUC to evaluate the network construction performance of different methods. Figure 2 shows the performance on the 1344-gene network, and the detailed performance on all other simulation settings can be seen in Supplementary Figures S2 and S3. From all simulation settings, we can see that as the sample size increased, the performance of all methods tended to improve; this is expected and consistent with previous research [41].

For the performance in constructing the global network structure (1344-gene network) measured by AUC, WGCNA and GeneNet performed best, followed by ARACNE, and SPACE performed the worst (Figure 2A). The differences decreased as the number of samples increased. When the sample size reached 1000, all the method performed very well (with AUC close to 1). In the five other sizes of networks (Figure S2), the performance of various methods were similar for smaller networks. The Bayesian method could only compute the smaller networks and failed for all the datasets involving 1,000 samples on our computing equipment. It performed comparably to the other methods on the smallest network (17 genes) as can be seen in Figure S2.

For identifying a few connection with high specificity, SPACE outperformed the other methods across all simulation settings followed by GeneNet and then WGCNA (Figure 2B). In the five other sizes of networks (Figure S3), SPACE and GeneNet were both the best-performing methods; SPACE slightly outperformed GeneNet for smaller numbers of samples (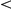
100).

The performance of the Bayesian networks is inconsistent with the general belief that Bayesian networks produce the most accurate networks. That is probably because BN methods perform better for smaller gene network construction and it could also be impacted by the simulation setups. In order to verify this, we studied the performance of constructing GRNs using the same 11-gene network as was used by Werhli et al [11]. We found that on this 11-gene network, the Bayesian method outperformed the other methods, which is consistent with the conclusion of Werhli's study (Figure S4). This may also be due to the underlying assumptions of each of these methods: Bayesian inference algorithms (typically) rely on categorical variables while partial-correlation and correlation-based algorithms assume a normal distribution for their variables. The data were simulated from a normal distribution to more accurately represent true gene expression experiments [40], thus we would expect degraded performance for Bayesian inference algorithms. A more specific comparison of performance is available in the supplementary material.

### Hub Gene Detection

Another important metric of interest in this study was the ability of a method to detect highly connected (or “hub”) genes within a network. These genes are often of particular biological interest as the activity of such hub genes may affect many genes in the biological network and hence drive disease status.

Performance in this area was measured by first calculating each gene's predicted connectivity (as described earlier) and comparing this against the binary classification of whether or not a gene was truly a hub gene in the true network. We computed ROC curves by changing the connectivity thresholds and used the AUC to measure the performance of detecting hub genes. We experimented with various cutoffs for the determination of hub genes, using either 4, 5, or 6 connections as the threshold. We obtained similar results on all three except for the smallest network (17 genes). This is because this network had only one gene which was classified as a hub gene when the threshold was set at either 5 or 6; on this network most methods performed perfectly or almost perfectly when using these thresholds. Three genes were classified as hub genes using a threshold of 4, which made for more meaningful performance measurements on this network, so we opted to use this threshold throughout the duration of the study.

**Figure 3:**
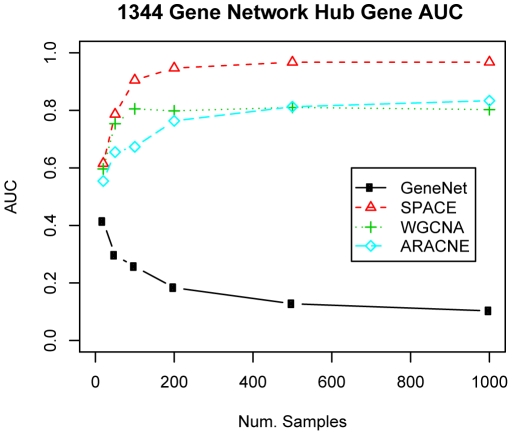
Comparison of the AUC performance on detecting hub genes. Measuring the performance of each method at detecting hub genes as measured by the Area Under the ROC Curve (AUC). Hub genes were classified as having 4 or more connections in the true network.

When examining the AUC for hub genes (Figure 3 and Supplementary Figure S5), SPACE was consistently the top performer for nearly all numbers of samples on all network sizes. The Bayesian method performed well on the smallest network, but was not competitive on the other network sizes. WGCNA performed well with very small numbers of samples, but was quickly outperformed by SPACE in every network.

GeneNet exhibited somewhat strange performance when dealing with hub genes. It was fairly competitive on the smaller networks, but produced severely degraded performance on the larger networks with AUCs well below 0.5 (which is the value of a random guess). This is likely due to the connectivity values produced by GeneNet. Most methods produced networks for which most connections were zeros or near-zero which produces near-zero connectivity values for most genes. When viewed as a histogram, the connectivity of all other algorithms was skewed to the right, while GeneNet had many more genes with high connectivity scores as shown in Supplementary Figure S6.

### Simulation Studies Under Non-Normal Distribution

**Figure 4:**
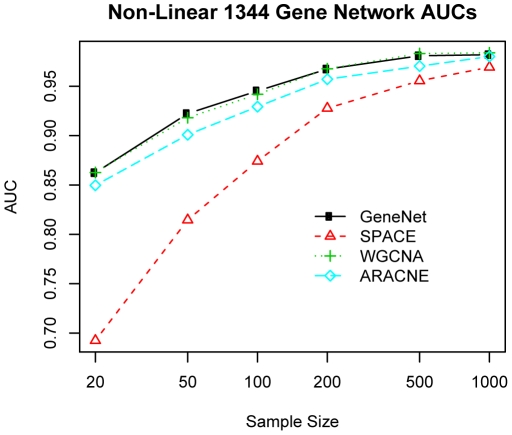
The AUCs for 1344-gene network in simulation 2 with non-normal distribution. The area under the curve using various network construction methods across various sample sizes on a network with 1344 genes.

To evaluate the performance of different methods when the underlying distribution is non-normal, we also simulated data under a non-normal distribution. In this simulation study, the expression data were simulated from a bimodal mixture of 2 normal distributions, which models the possible “on” and “off” status of a gene's expression. The mixture probability for each status is 0.5. The AUC curve for 1344-gene network with various methods and different numbers of samples were shown in Figure 4. The AUC and pAUC on all other simulation settings can be seen in Supplementary Figures S7 and S8. For the performance in constructing the global network structure, WGCNA and GeneNet still performed best, followed by ARACNE, and SPACE still performed the worst. The results were consistent with simulation study 1. If we use the performance of GeneNet and WGCNA as references, we notice that the performance of ARACNE improves while that of SPACE get worse. This could due to the fact that ARACNE does not rely on the normal assumption, while SPACE highly does, so under a non-normal distribution the performance of ARACNE improves while that of SPACE decreases relative to GeneNet and WGCNA.

### Computational Complexity and Program Usability

Aside from accuracy, one of the important attributes of each algorithm is computational complexity. In environments lacking a strong computational infrastructure, certain algorithms may be unfeasible (especially when processing a large dataset). The Bayesian algorithm was the only algorithm that caused concerns. Most other methods would finish computing within minutes on standard desktop hardware for any of the datasets we examined. The Bayesian algorithm, on the other hand, typically took hours or even days to compute and required advanced hardware.

Program usability is also a consideration, especially among groups with no special expertise in computer programming. Among the selected implementations, no specific one stands out as more or less usable than the others. Each provides a command-line interface; usability would largely be determined by a user's familiarity with a particular platform (R and/or C, or Matlab or JAVA). The only notable user-friendly feature offered in these packages was that the WCGNA package and ARACNE software provide many useful network analysis and visualization functions which are very convenient.

Also important is the ability to process and store the resultant networks in either adjacency list or matrix format. SPACE, which is designed to operate on sparse matrices, produced networks in which only approximately 10% of the network was non-zero, making it much easier to store in a compressed format than the networks produced by the other methods (which typically had 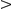
99% non-zero matrices).

### Empirical Study In E. coli

**Figure 5:**
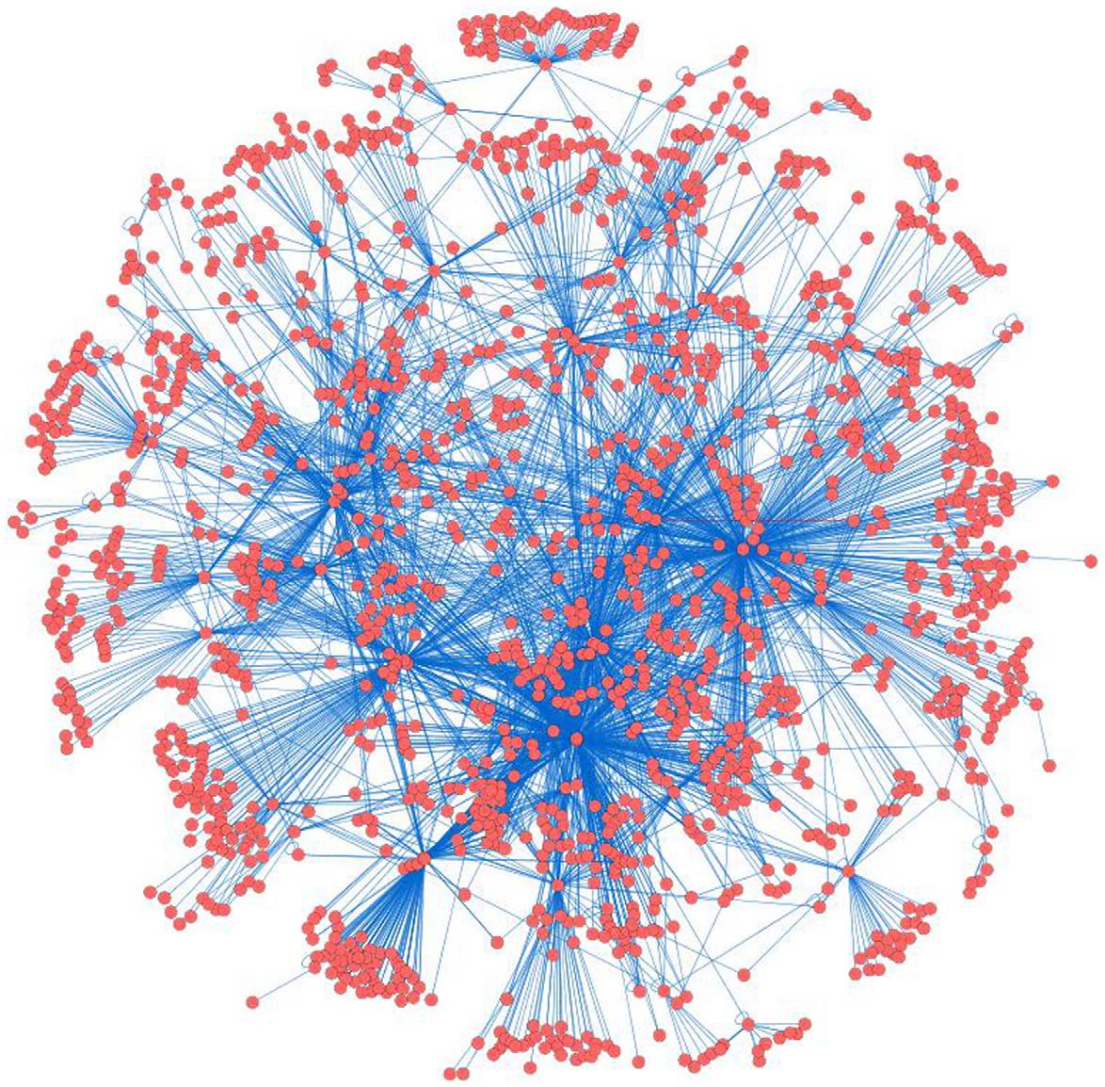
The transcriptional regulatory network for E. coli derived from the RegulonDB database. Each red dot is a gene, and a blue line between genes indicates a connection.

**Figure 6:**
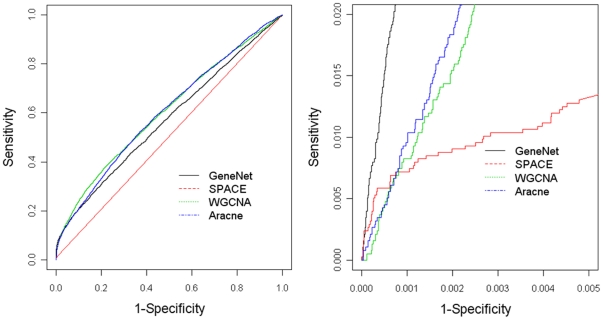
The performance in constructing gene regulatory network in E. coli. Left: The entire ROC curves using various network construction methods; Right: The corner of ROC with high specificity.

The predictive performance of our approach was tested using the Escherichia coli (E.coli) gene expression database entitled M3D (Many Microbe Microarrays Database [42]). The dataset contains 524 arrays measured under 264 experimental conditions. The data were measured using Affymetrix GeneChip E.coli Genome arrays with 4292 gene probes. The arrays measured under the same experimental conditions were averaged. From the gene expression data matrix, we used SPACE, GeneNet, WGCNA and ARACNE methods to derive the gene network in E. coli. To evaluate the performance, we used the transcriptional regulatory network from the RegulonDB [43], which provides the regulation targets of the transcriptional factors in E coli. An overview of the network is shown in Figure 5. The ROC curves of various methods were shows in Figure 6. For this real data example, the thresholds for a false positive rate of 0.005 are 0.05, 2.4E-7, 0.12, 0.37 for GeneNet, SPACE, WGCNA and Aracne, respectively. For constructing the global network structure, WGCNA and ARACNE performed the best, followed by GeneNet, with SPACE performing the worst. On the other hand, for identifying a few connections with high specificity, GeneNet and SPACE performed better than the others. Overall, the results were relatively consistent with the simulation studies.

## Discussion

We have measured the performance of various gene regulatory network construction methodologies against various sizes of simulated data with different numbers of samples. From this, a few conclusions can be drawn.

First, WGCNA and ARACNE performed well in constructing the global network, while SPACE did well in identifying a few connections with high specificity. GeneNet performed well in both aspects, but it is not suitable for identifying the hub genes, which can often be of biological interest. In the simulation study, SPACE performed well in identifying the hub genes as shown in Figure 3. Since there is no a single method that outperforms other methods in all aspects, the user should choose an appropriate method based on the purpose of the study.

In applying these methods to the real E. coli data, WGCNA and ARACNE performed best, which may indicate that these two methods are relatively more robust. Overall, the performance in real data seemed to be worse than that in the simulation study, and there are several possible reasons: (1) the real biological network is much more complex than the simulation study; (2) many true connections in this network are still unknown; (3) some of the connections in RegulonDB may not be supported by gene expression data [44]. Surprisingly, SPACE performed poorly in constructing the global network, which is because the SPACE algorithm uses an L1 penalty to shrink most of partial correlation to zero. If we manually decrease the penalty term, the performance improved as fewer partial correlations were shrunk to zero, but it also became much more computationally intensive. In this study, we used default parameters or recommended settings for each method whenever possible for a fair comparison. So, here we still present the results based the default setting of SPACE algorithm.

Another conclusion which can be drawn is that as sample sizes increase, the accuracy also increases. For the number of samples tested (20–1,000), the most significant performance improvements were obtained at the beginning; they began to saturate as the number of samples approached 1,000. This demonstrates that having thousands of samples may not offer significant performance improvements.

Also, this study demonstrates that it is feasible to use current techniques to generate accurate, informative networks even with dozens or hundreds of genes. Several algorithms scaled to such environments well without requiring sophisticated computational resources.

One disadvantage of probabilistic-network-based methods is the discretization of data. It is generally preferred to discretize into a small number of “buckets” which directly represent an underlying biological observation when using probability networks. To this end, data is typically discretized into binary buckets (implying that a gene is either “on” or “off”) or ternary buckets (signifying “under-expressed,” “normally expressed,” and “over expressed”). Unfortunately, fitting the data into any reasonable number of buckets will result in substantial data loss.

Finally, we found that the Bayesian methods did not scale to larger networks well. Because of the computational complexity as well as the memory requirements, these methods – as currently implemented – are not the ideal choice for such large networks. WGCNA, GeneNet, ARACNE and SPACE, on the other hand, were designed to construct the gene network at very large scales. Also, it worth mentioning that the WGCNA package provides several useful tools to facilitate the analysis and visualization of resulting networks, including tools to identify subnetworks and an interface to Cytoscape. The WGCNA package can be used for not only constructing gene networks but also for detecting modules/sub-networks, identifying hub genes, and selecting candidate genes as biomarkers.

## Supporting Information

Figure S1 **ROC Curves for the 17 Gene Network.** The Receiver Operating Characteristic (ROC) curves for the 17 gene network which will quantified using the Area Under the Curve (AUC).
(TIF)

Figure S2 **AUCs for All Network Sizes.** The relationship between sample size and the area under the ROC curve (AUC) values for each network size and network construction method.
(TIF)

Figure S3
**pAUCs for All Network Sizes.** The relationship between sample size and the partial area under the ROC curve (AUC) values for FPR
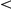
0.005 for each network size and network construction method.(TIF)

Figure S4 **AUCs on 11-gene network.** The AUCs for each method on Werhli's 11 gene network. As Werhli had demonstrated, the Bayesian method performs quite well compared to other network construction methods.
(TIF)

Figure S5 **Hub Gene Performance.** For all network sizes, the figure shows the relationship between the sample size and the area under the ROC curve (AUC) regarding each method's classification of hub genes by classifying a hub gene as a gene with 4 or more connections.
(TIF)

Figure S6 **Histograms of Connectivity Scores for Various Methods.** Depicts the differences in the distributions of the gene's connectivity values (weighted degree) across the different methods on the 44 gene network with 200 samples. Scores were normalized to [0,1] by dividing all predicted connectivity scores by the maximum connectivity score in that setup. Note that GeneNet is skewed such that most genes are highly-connected when compared to the other methods. This causes problems later on when evaluating the AUC scores for the classification of hub genes for this method.
(TIF)

Figure S7 **AUCs for All Network Sizes in Simulation Study 2.** The relationship between sample size and the area under the ROC curve (AUC) values for each network size and network construction method in simulation study 2 which uses non-normal distribution assumptions for expression values.
(TIF)

Figure S8
**pAUCs for All Network Sizes in simulation study 2.** The relationship between sample size and the area under the ROC curve (AUC) values for FPR
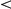
0.005 for each network size and network construction method in simulation study 2 which uses non-normal distribution assumptions for expression values.(TIF)

Sweave S1 **Sweave Documentation for all Analysis.** Documents the creation and analysis of the reverse-engineered methods for all network types and network construction methods.
(PDF)
