# Supplementary figures and images for: Comparing Statistical Methods for Constructing Large Scale Gene Networks
Source: PLoS One. 2012 Jan 17;7(1):e29348. doi: 10.1371/journal.pone.0029348 (PMC3260142; doi:10.1371/journal.pone.0029348)

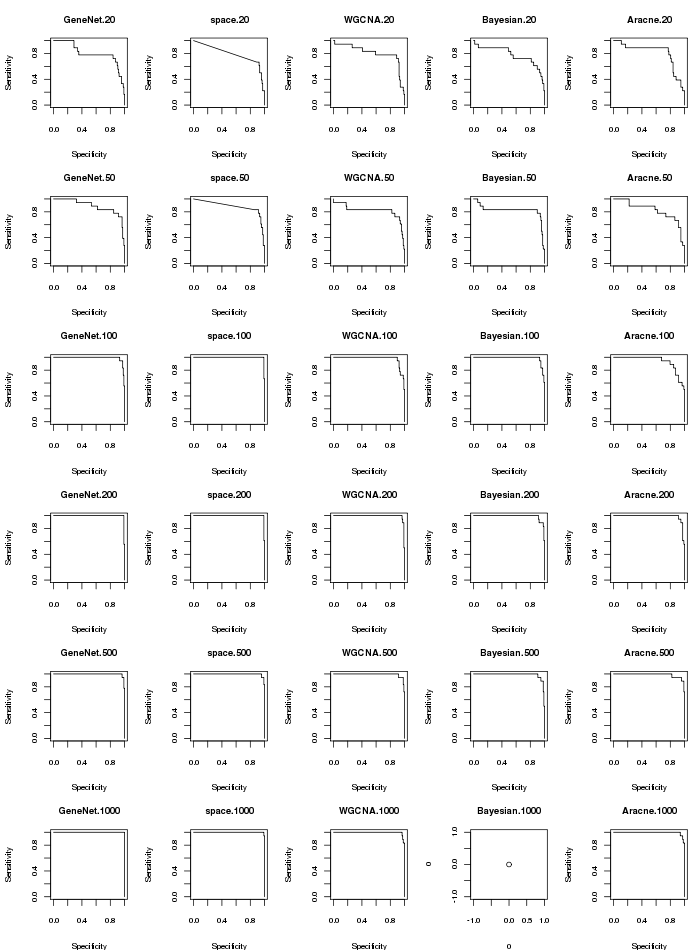

Supplement: Figure S1 — ROC Curves for the 17 Gene Network. The Receiver Operating Characteristic (ROC) curves for the 17 gene network which will quantified using the Area Under the Curve (AUC). (TIF) [file pone.0029348.s001.tif]

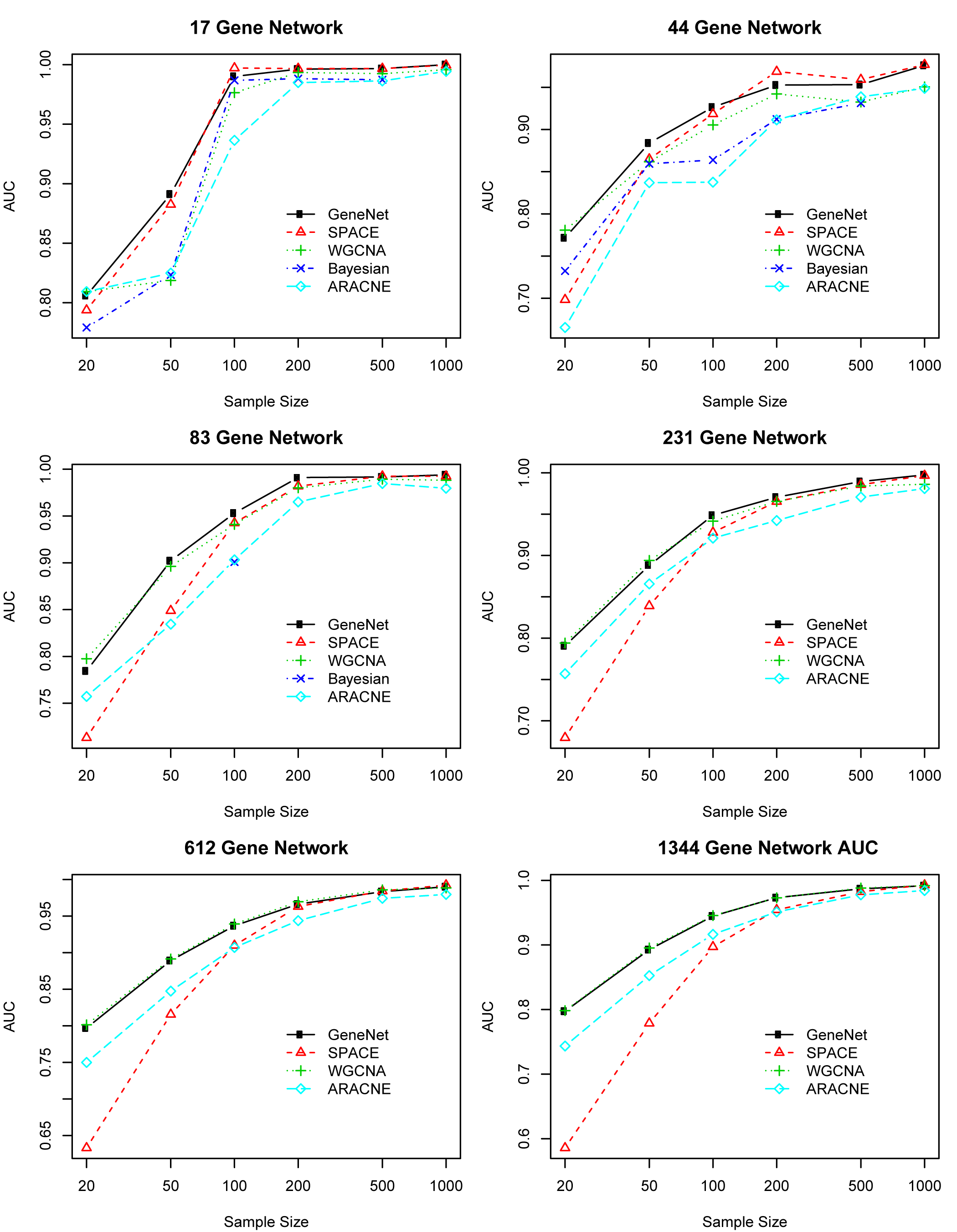

Supplement: Figure S2 — AUCs for All Network Sizes. The relationship between sample size and the area under the ROC curve (AUC) values for each network size and network construction method. (TIF) [file pone.0029348.s002.tif]

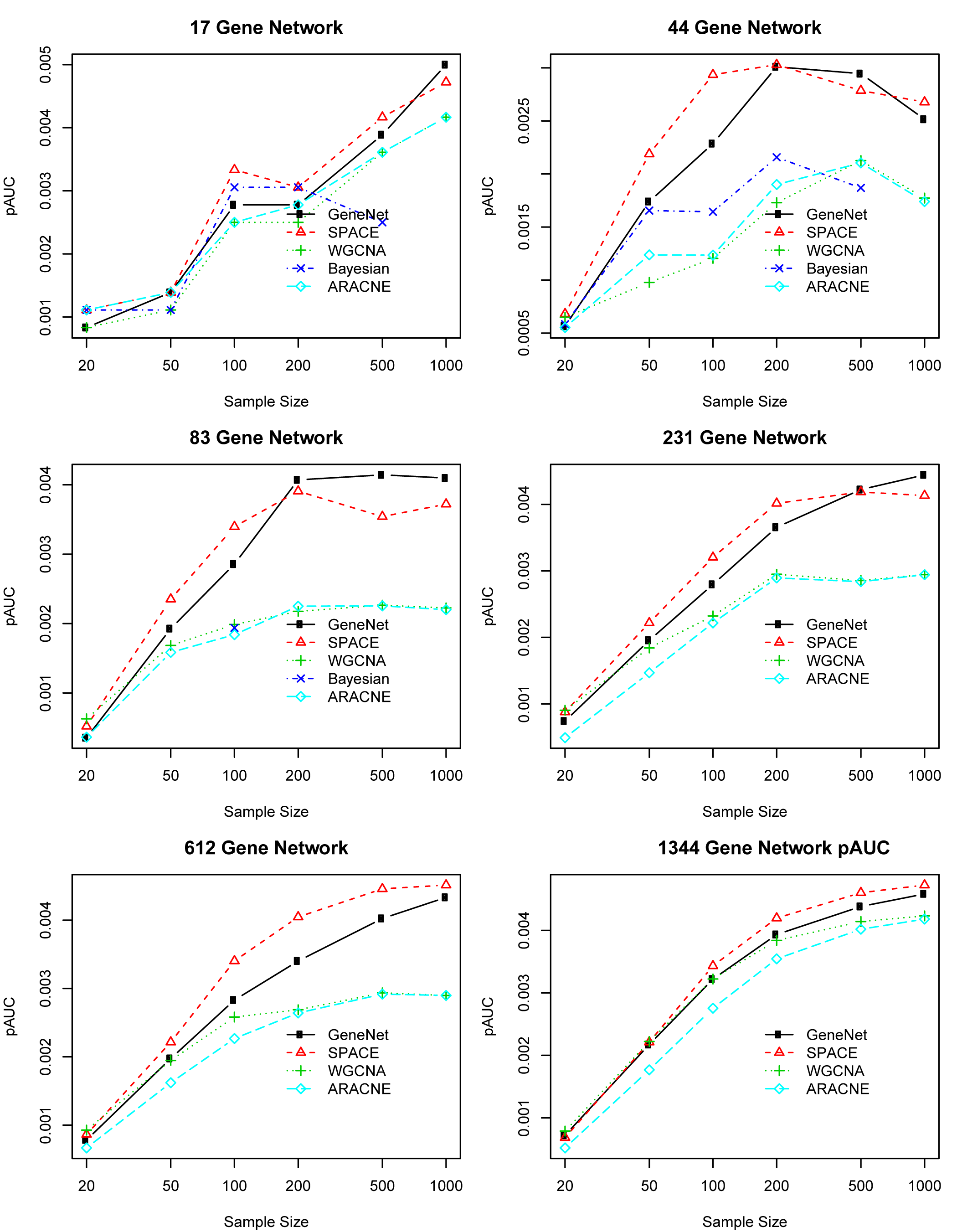

Supplement: Figure S3 — pAUCs for All Network Sizes. The relationship between sample size and the partial area under the ROC curve (AUC) values for FPR0.005 for each network size and network construction method. (TIF) [file pone.0029348.s003.tif]

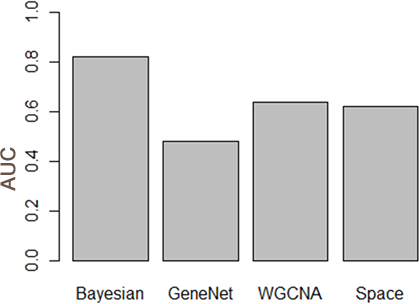

Supplement: Figure S4 — AUCs on 11-gene network. The AUCs for each method on Werhli's 11 gene network. As Werhli had demonstrated, the Bayesian method performs quite well compared to other network construction methods. (TIF) [file pone.0029348.s004.tif]

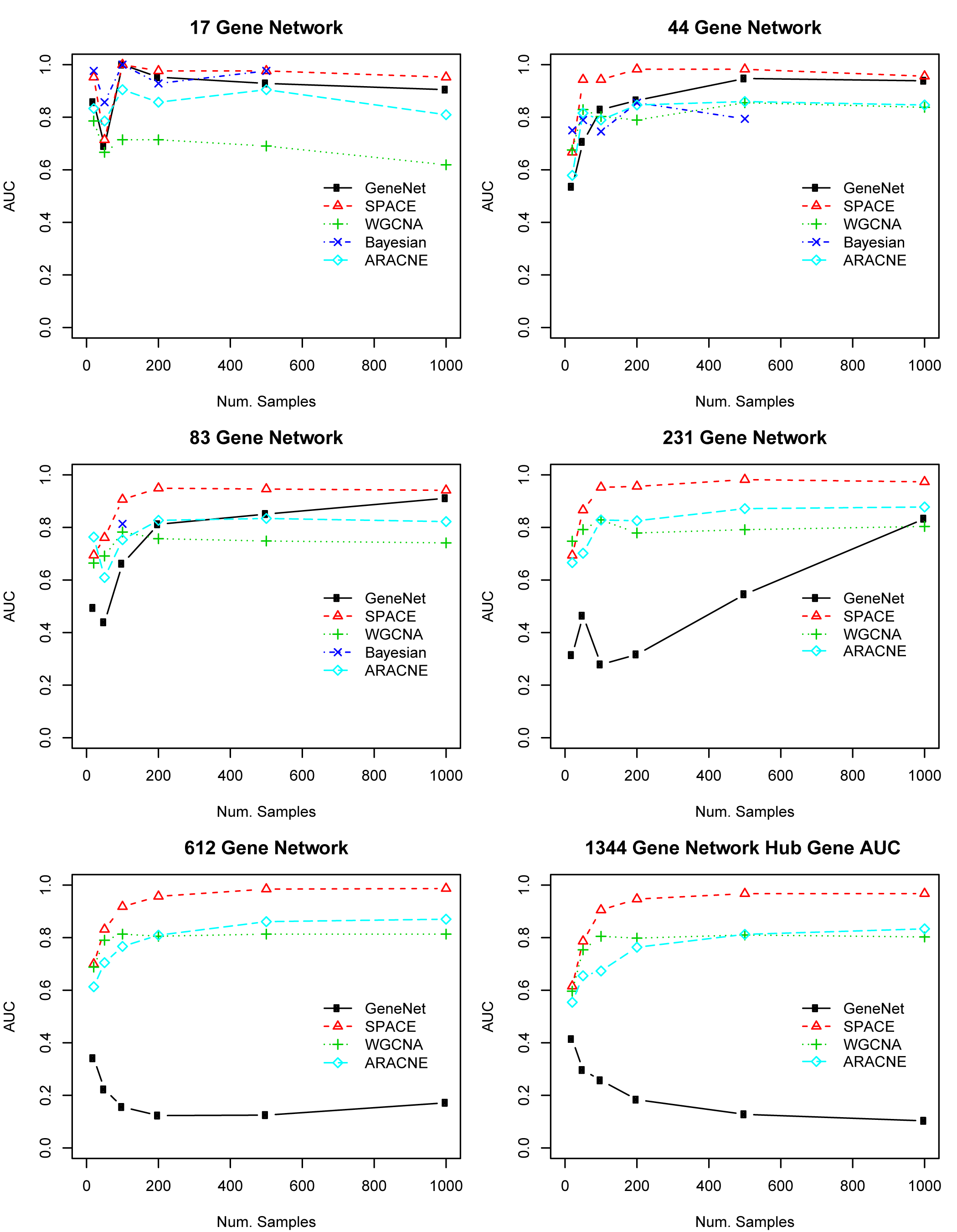

Supplement: Figure S5 — Hub Gene Performance. For all network sizes, the figure shows the relationship between the sample size and the area under the ROC curve (AUC) regarding each method's classification of hub genes by classifying a hub gene as a gene with 4 or more connections. (TIF) [file pone.0029348.s005.tif]

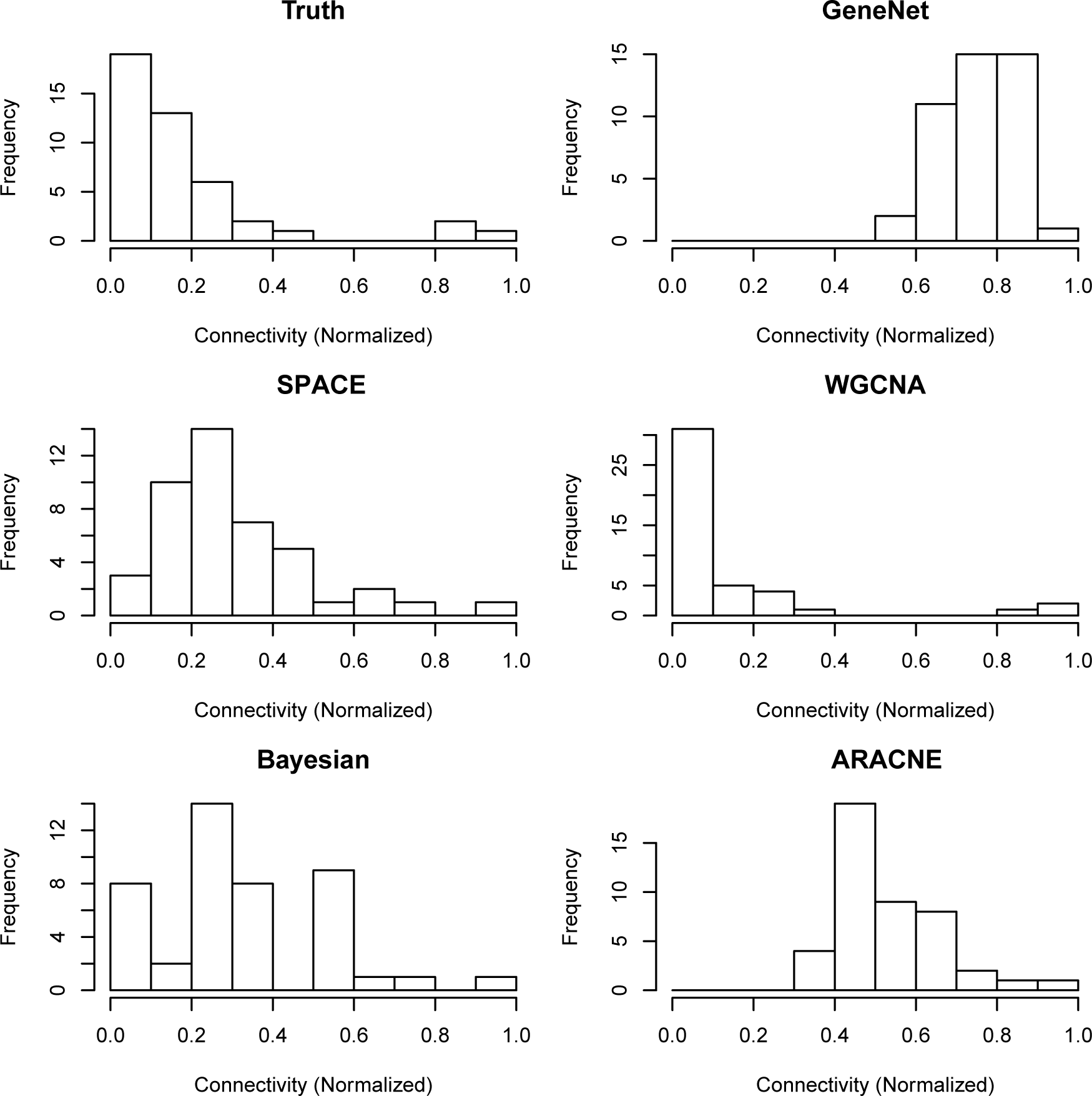

Supplement: Figure S6 — Histograms of Connectivity Scores for Various Methods. Depicts the differences in the distributions of the gene's connectivity values (weighted degree) across the different methods on the 44 gene network with 200 samples. Scores were normalized to [0,1] by dividing all predicted connectivity scores by the maximum connectivity score in that setup. Note that GeneNet is skewed such that most genes are highly-connected when compared to the other methods. This causes problems later on when evaluating the AUC scores for the classification of hub genes for this method. (TIF) [file pone.0029348.s006.tif]

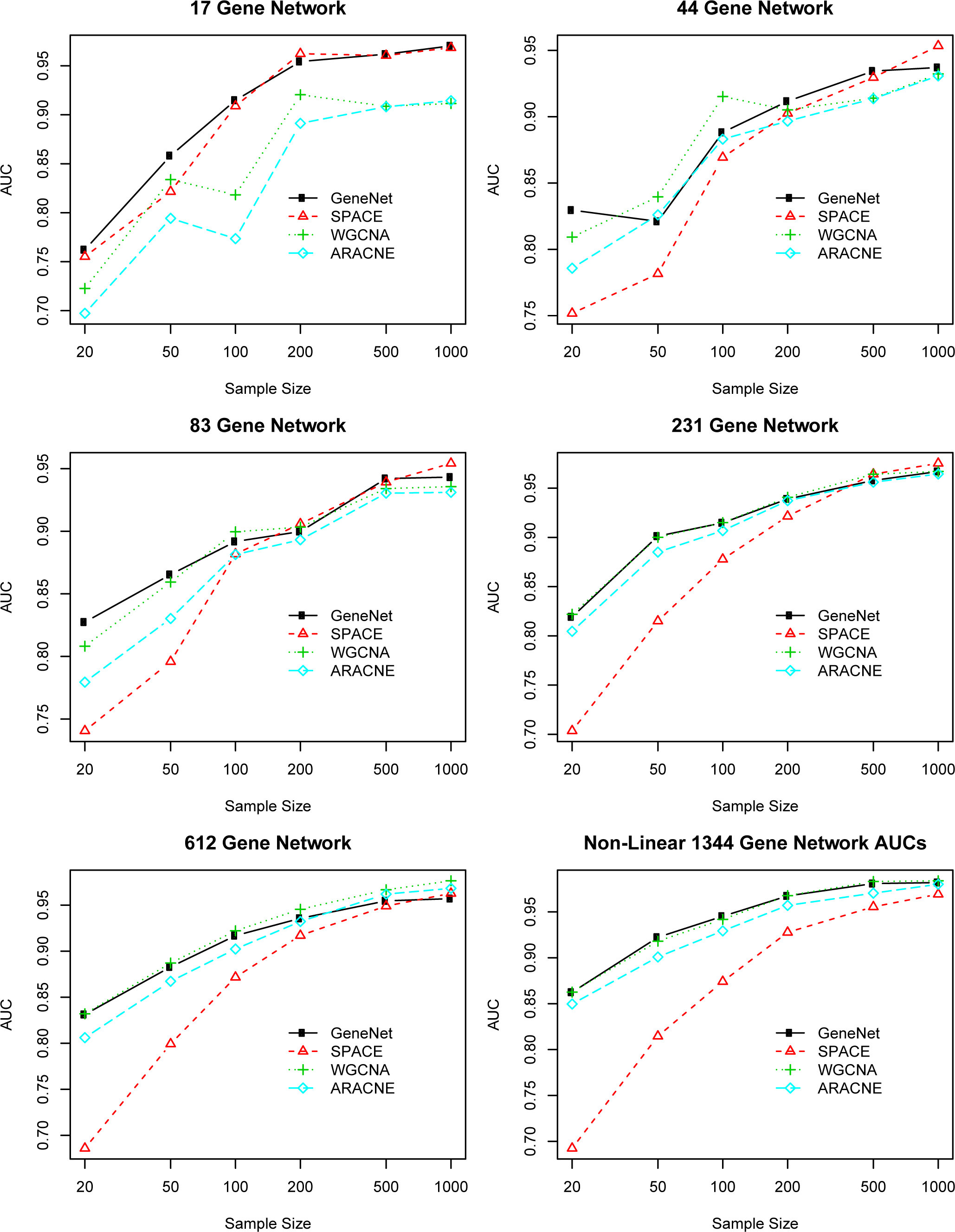

Supplement: Figure S7 — AUCs for All Network Sizes in Simulation Study 2. The relationship between sample size and the area under the ROC curve (AUC) values for each network size and network construction method in simulation study 2 which uses non-normal distribution assumptions for expression values. (TIF) [file pone.0029348.s007.tif]

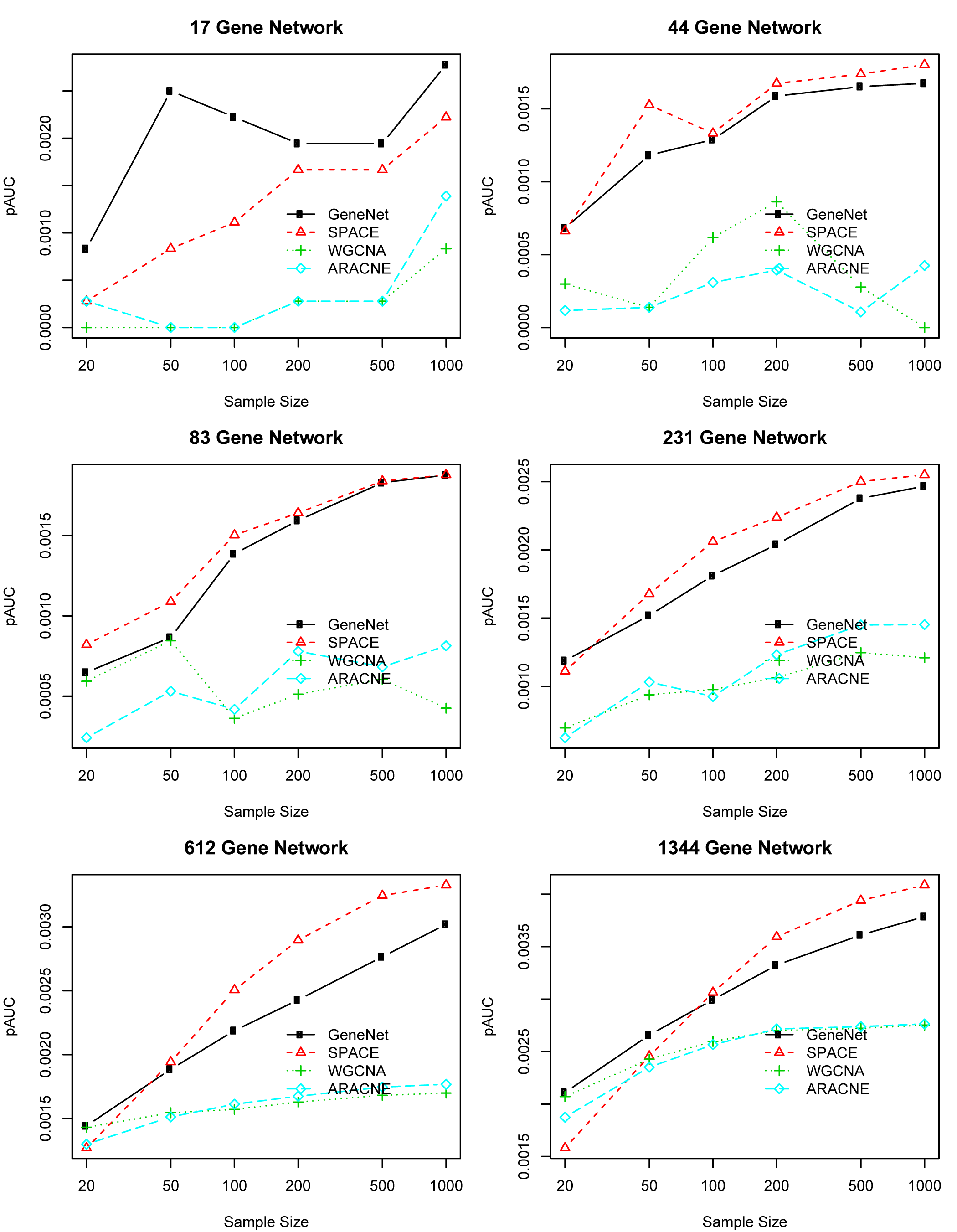

Supplement: Figure S8 — pAUCs for All Network Sizes in simulation study 2. The relationship between sample size and the area under the ROC curve (AUC) values for FPR0.005 for each network size and network construction method in simulation study 2 which uses non-normal distribution assumptions for expression values. (TIF) [file pone.0029348.s008.tif]
