# Supplementary material for: Comparing Statistical Methods for Constructing Large Scale Gene Networks
Source: PLoS One. 2012 Jan 17;7(1):e29348. doi: 10.1371/journal.pone.0029348 (PMC3260142; doi:10.1371/journal.pone.0029348)
Supplement: Sweave S1 — Sweave Documentation for all Analysis. Documents the creation and analysis of the reverse-engineered methods for all network types and network construction methods. (PDF) [file pone.0029348.s009.pdf]

Compare Network Engines

Jeffrey D. Allen

July 28, 2011

This file will document the process by which we examine and compare various methods of constructing genetic dependency graphs.

1 Data Files

Two types of files are used and are assumed to already exist. First, is a set of files which represent the simulated expression levels for a set of genes across various samples. Second is the truth of the underlying network being simulated in these samples. For the sake of simplicity, the truth is stored in adjacency-list format.

We will first construct a list of names for the various networks. These names represent the different categories of networks we'll be testing. For instance, we simulated a "tiny" network with only a few genes, and a "large" network with hundreds of genes, etc.

```
> networkSizes = c("tiny", "small", "moderate", "middle", "large",
+     "huge")
```

For each of these networks, we simulated different experiments which varied in the number of samples they contained. This will test the ability to extract meaning from various numbers of samples from networks of various sizes.

```
> sampleCounts = c(20, 50, 100, 200, 500, 1000)
```

Next we'll convert these names and sample sizes into the names of the files:

```
> files = matrix(nrow = length(networkSizes), ncol = length(sampleCounts))
> for (i in 1:length(networkSizes)) {
+     files[i, ] = paste(networkSizes[i], " nSamp", sampleCounts,
+         ".csv", sep = "")
+ }
> files

     [,1]                    [,2]                    [,3]
[1,] "tiny nSamp20.csv"     "tiny nSamp50.csv"     "tiny nSamp100.csv"
[2,] "small nSamp20.csv"    "small nSamp50.csv"    "small nSamp100.csv"
[3,] "moderate nSamp20.csv" "moderate nSamp50.csv" "moderate nSamp100.csv"
[4,] "middle nSamp20.csv"   "middle nSamp50.csv"   "middle nSamp100.csv"
[5,] "large nSamp20.csv"    "large nSamp50.csv"    "large nSamp100.csv"
[6,] "huge nSamp20.csv"     "huge nSamp50.csv"     "huge nSamp100.csv"
     [,4]                    [,5]                    [,6]
[1,] "tiny nSamp200.csv"     "tiny nSamp500.csv"     "tiny nSamp1000.csv"
[2,] "small nSamp200.csv"    "small nSamp500.csv"    "small nSamp1000.csv"
[3,] "moderate nSamp200.csv" "moderate nSamp500.csv" "moderate nSamp1000.csv"
[4,] "middle nSamp200.csv"   "middle nSamp500.csv"   "middle nSamp1000.csv"
[5,] "large nSamp200.csv"    "large nSamp500.csv"    "large nSamp1000.csv"
[6,] "huge nSamp200.csv"     "huge nSamp500.csv"     "huge nSamp1000.csv"
```

At this point, all of the files of a certain network can be accessed with:

```
> tinyFiles <- paste("../../Simulations/", files[1, ], sep = "")
> tinyFiles
```

```
[1] "../../Simulations/tiny nSamp20.csv"
[2] "../../Simulations/tiny nSamp50.csv"
[3] "../../Simulations/tiny nSamp100.csv"
[4] "../../Simulations/tiny nSamp200.csv"
[5] "../../Simulations/tiny nSamp500.csv"
[6] "../../Simulations/tiny nSamp1000.csv"
```

and specific files can be read in with:

```
> data <- as.matrix(read.csv(tinyFiles[5], header = T, row.names = 1))
```

which presents the data file as a data.frame which gets converted to a matrix for ease of computational access later on. Note that the gene names (in this case, integral identifiers) serve as row labels while the sample identifiers are the column names. Different programs will request this data in different formats, but most should be compatible with either this or its transpose. Now that we have the samples available in a convenient format, we can begin processing the various methods.

2 Processing

2.1 Output Format

The goal of this analysis is to put all of the simulation results into aggregate files which can be used to compare the performance of each method. These files will be in adjacency-list-format and will exhaust every possible connection that isn't itself a cycle (i.e. any gene pointing to any other gene will be an option). Then the columns will each represent the output of some method.

However, all of the methods will work on matrices as input and output, so we'll need a function to convert our matrix into a list of standard format.

```
Package WGCNA version 1.11-3 loaded.

> matrixToList <- function(mat, includeGeneIDs = TRUE) {
+     arSize <- length(length(mat)/2 - dim(mat)[1]/2)
+     for (a in 1:dim(mat)[1]) {
+         for (b in 1:dim(mat)[2]) {
+             strength <- max(abs(mat[a, b]), abs(mat[b, a]))
+             mat[min(a, b), max(a, b)] <- strength
+             mat[max(a, b), min(a, b)] <- 0
+             if (a == b) {
+                 mat[a, b] = 0
+             }
+         }
+     }
+     outputPointer <- 1
+     weights <- array(arSize)
+     sources <- array(arSize)
+     targets <- array(arSize)
+     for (a in 1:(dim(mat)[1] - 1)) {
+         start <- outputPointer
+         end <- dim(mat)[2] + outputPointer - a - 1
+         length <- end - start + 1
+         weights[start:end] <- mat[a, (a + 1):dim(mat)[2]]
+         if (includeGeneIDs) {
+             sources[start:end] <- colnames(mat)[a]
+             targets[start:end] <- colnames(mat)[a + 1:dim(mat)[2]]
+         }
+         outputPointer = outputPointer + length
+     }
+     if (includeGeneIDs) {
+         output <- matrix(nrow = length(sources), ncol = 3)
+         output[, 1] = sources
```

```
+         output[, 2] = targets
+         output[, 3] = weights
+         colnames(output) <- c("Source", "Target", "Strength")
+     }
+     else {
+         output <- matrix(nrow = (outputPointer - 1), ncol = 1)
+         output[, 1] = weights
+     }
+     output
+ }
```

2.2 Truth

Because the truth files will be in adjacency list format, we'll need a function to convert the truth function to a a matrix so they can be used by the above function. (Though we could convert directly to list format, this method will be simple to write/verify/debug.)

```
> getTruthMatrix <- function(networkSizeID) {
+     truthFile <- paste("../../Simulations/truth ", networkSizes[networkSizeID],
+         ".csv", sep = "")
+     data <- read.csv(truthFile, header = T)
+     sources <- as.numeric(data$Source)
+     targets <- as.numeric(data$Target)
+     weights <- abs(as.numeric(data$regulation))
+     data <- as.matrix(read.csv(paste("../../Simulations/", files[networkSizeID,
+         1], sep = ""), header = T, row.names = 1))
+     geneList <- as.numeric(rownames(data))
+     mat <- listToMatrix(geneList, sources, targets, weights)
+     as.matrix(mat)
+ }
> listToMatrix <- function(geneList, g1, g2, weights) {
+     g1 <- as.matrix(g1)
+     g2 <- as.matrix(g2)
+     weights <- as.matrix(weights)
+     mat <- matrix(rep(0, length(geneList) * length(geneList)),
+         nrow = length(geneList), ncol = length(geneList))
+     rownames(mat) <- geneList
+     colnames(mat) <- geneList
+     for (i in 1:length(g1)) {
+         a <- which(geneList == g1[i])
+         b <- which(geneList == g2[i])
+         mat[a, b] = weights[i]
+     }
+     mat
+ }
```

2.3 WGCNA

WGCNA must be downloaded and installedf first. At the time of writing, the package is available at `http://www.genetics.ucla.edu/labs/horvath/CoexpressionNetwork/Rpackages/WGCNA/`. Additionall, the following instruction will install the necessary prerequisite files:

```
install.packages(c("fields", "impute", "dynamicTreeCut", "qvalue", "flashClust", "Hmisc") )
```

We then use the following function to use WGCNA to calculate the adjacency matrix of some set of samples.

```
> adj <- adjacency(t(data))
```

2.4 GeneNet

Extracting a network from GeneNet is just a bit more complex.

```
> buildGeneNet <- function(data) {
+     inferred.pcor <- ggm.estimate.pcor(t(data))
+     test.results <- ggm.test.edges(inferred.pcor, direct = FALSE,
+         plot = FALSE)
+     names <- rownames(data)
+     n1 <- as.numeric(names[test.results$node1])
+     n2 <- as.numeric(names[test.results$node2])
+     res <<- data.frame(g1 = n1, g2 = n2, val = abs(test.results$pcor))
+     geneList <- as.numeric(rownames(data))
+     as.matrix(listToMatrix(geneList, n1, n2, abs(test.results$pcor)))
+ }
```

2.5 Space

Space will require a bit more work as there are some parameters which must be set. Also, Space strips the row names off of the data set, so we'll have to restore those.

```
> buildSpace <- function(data) {
+     n = ncol(data)
+     p = nrow(data)
+     alpha = 1
+     l1 = 1/sqrt(n) * qnorm(1 - alpha/(2 * p^2))
+     iter = 3
+     result <- space.joint(t(data), lam1 = 0, lam2 = 0, weight = 1,
+         iter = iter)$ParCor
+     colnames(result) <- rownames(data)
+     rownames(result) <- rownames(data)
+     as.matrix(result)
+ }
```

2.6 Bayesian

The Bayesian method is unique from the others in that it is not generated in R, but is based on a Matlab program. For this method, then, we'll need to read in the results from Matlab as .csv files and deal with converting the data into a format that will be compatible with the functions created above.

Assuming we're given a format of the raw matrix (no header or row-labels), we'll read that data in and process from there.

```
> getBayesianMat <- function(networkSizeID, sampleCountID) {
+     data <- as.matrix(read.csv(paste("../../Simulations/", files[networkSizeID,
+         1], sep = ""), header = T, row.names = 1))
+     geneList <- as.numeric(rownames(data))
+     fileName <- paste("../../Results/Wehrli/Output-MCMC-SCORES",
+         networkSizes[networkSizeID], "TransposeNSamp", sampleCounts[sampleCountID],
+         ".csv.mat.csv", sep = "")
+     if (file.exists(fileName)) {
+         bayesianMat <- read.csv(fileName, header = FALSE)
+     }
+     else {
+         bayesianMat <- matrix(NA, nrow = length(geneList), ncol = length(geneList))
+     }
+     rownames(bayesianMat) <- geneList
+     colnames(bayesianMat) <- geneList
+     as.matrix(bayesianMat)
+ }
```

3 Aggregate Files

By building an aggregate file of an entire network, we can examine the performance of various algorithms or sample sizes easily. First, we'll want a way to setup an adjacency list structure which can be used as a template for the rest of the data. This structure should contain the gene identifiers for each connection as well as the true value of that connection.

```
> getTruthCols <- function(networkSizeID) {
+     truth <- getTruthMatrix(networkSizeID)
+     truth <- matrixToList(truth)
+     colnames(truth) <- c(colnames(truth)[1:2], "Truth")
+     truth
+ }
```

Next, we'll want a function to calculate the aggregates across all 4 methods for some network size and some number of samples.

```
> getAgg <- function(networkSizeID, sampleCountID) {
+     data <- as.matrix(read.csv(paste("../../Simulations/", files[networkSizeID,
+         sampleCountID], sep = ""), header = T, row.names = 1))
+     agg <- cbind(matrixToList(buildGeneNet(data), FALSE), matrixToList(buildSpace(data),
+         FALSE), matrixToList(adjacency(t(data)), FALSE), matrixToList(getBayesianMat(networkSizeID,
+         sampleCountID), FALSE))
+     colnames(agg) <- c(paste("GeneNet-", sampleCounts[sampleCountID],
+         sep = ""), paste("space-", sampleCounts[sampleCountID],
+         sep = ""), paste("WGCNA-", sampleCounts[sampleCountID],
+         sep = ""), paste("Bayesian-", sampleCounts[sampleCountID],
+         sep = ""))
+     agg
+ }
```

Finally, we'll want a way to iterate the above method on all sample sizes, so that we can build a file containing all of the information about one network size.

```
> getAllAggs <- function(networkSizeID) {
+     output <- getTruthCols(networkSizeID)
+     for (samples in 1:length(sampleCounts)) {
+         output <- cbind(output, getAgg(networkSizeID, samples))
+     }
+     output
+ }
```

This method can be called and the output written to a CSV file using something like:

```
> for (netSize in 1:4) {
+     write.csv(getAllAggs(netSize), paste("../../Results/res5.newAgg",
+         networkSizes[netSize], ".csv", sep = ""), row.names = F,
+         quote = F)
+ }
```

```
Estimating optimal shrinkage intensity lambda (correlation matrix): 0.7948

Estimate (local) false discovery rates (partial correlations):
Step 1... determine cutoff point
Step 2... estimate parameters of null distribution and eta0
Step 3... compute p-values and estimate empirical PDF/CDF
Step 4... compute q-values and local fdr

[1] "iter=1"
[1] "iter=2"
[1] "iter=3"
Estimating optimal shrinkage intensity lambda (correlation matrix): 0.4522
```

```
Estimate (local) false discovery rates (partial correlations):
Step 1... determine cutoff point
Step 2... estimate parameters of null distribution and eta0
Step 3... compute p-values and estimate empirical PDF/CDF
Step 4... compute q-values and local fdr

[1] "iter=1"
[1] "iter=2"
[1] "iter=3"
Estimating optimal shrinkage intensity lambda (correlation matrix): 0.3156

Estimate (local) false discovery rates (partial correlations):
Step 1... determine cutoff point
Step 2... estimate parameters of null distribution and eta0
Step 3... compute p-values and estimate empirical PDF/CDF
Step 4... compute q-values and local fdr

[1] "iter=1"
[1] "iter=2"
[1] "iter=3"
Estimating optimal shrinkage intensity lambda (correlation matrix): 0.2183

Estimate (local) false discovery rates (partial correlations):
Step 1... determine cutoff point
Step 2... estimate parameters of null distribution and eta0
Step 3... compute p-values and estimate empirical PDF/CDF
Step 4... compute q-values and local fdr

[1] "iter=1"
[1] "iter=2"
[1] "iter=3"
Estimating optimal shrinkage intensity lambda (correlation matrix): 0.0939

Estimate (local) false discovery rates (partial correlations):
Step 1... determine cutoff point
Step 2... estimate parameters of null distribution and eta0
Step 3... compute p-values and estimate empirical PDF/CDF
Step 4... compute q-values and local fdr

[1] "iter=1"
[1] "iter=2"
[1] "iter=3"
Estimating optimal shrinkage intensity lambda (correlation matrix): 0.0485

Estimate (local) false discovery rates (partial correlations):
Step 1... determine cutoff point
Step 2... estimate parameters of null distribution and eta0
Step 3... compute p-values and estimate empirical PDF/CDF
Step 4... compute q-values and local fdr

[1] "iter=1"
[1] "iter=2"
[1] "iter=3"
Estimating optimal shrinkage intensity lambda (correlation matrix): 0.8083

Estimate (local) false discovery rates (partial correlations):
Step 1... determine cutoff point
Step 2... estimate parameters of null distribution and eta0
```

```
Step 3... compute p-values and estimate empirical PDF/CDF
Step 4... compute q-values and local fdr

[1] "iter=1"
[1] "iter=2"
[1] "iter=3"
Estimating optimal shrinkage intensity lambda (correlation matrix): 0.5684

Estimate (local) false discovery rates (partial correlations):
Step 1... determine cutoff point
Step 2... estimate parameters of null distribution and eta0
Step 3... compute p-values and estimate empirical PDF/CDF
Step 4... compute q-values and local fdr

[1] "iter=1"
[1] "iter=2"
[1] "iter=3"
Estimating optimal shrinkage intensity lambda (correlation matrix): 0.4227

Estimate (local) false discovery rates (partial correlations):
Step 1... determine cutoff point
Step 2... estimate parameters of null distribution and eta0
Step 3... compute p-values and estimate empirical PDF/CDF
Step 4... compute q-values and local fdr

[1] "iter=1"
[1] "iter=2"
[1] "iter=3"
Estimating optimal shrinkage intensity lambda (correlation matrix): 0.243

Estimate (local) false discovery rates (partial correlations):
Step 1... determine cutoff point
Step 2... estimate parameters of null distribution and eta0
Step 3... compute p-values and estimate empirical PDF/CDF
Step 4... compute q-values and local fdr

[1] "iter=1"
[1] "iter=2"
[1] "iter=3"
Estimating optimal shrinkage intensity lambda (correlation matrix): 0.1224

Estimate (local) false discovery rates (partial correlations):
Step 1... determine cutoff point
Step 2... estimate parameters of null distribution and eta0
Step 3... compute p-values and estimate empirical PDF/CDF
Step 4... compute q-values and local fdr

[1] "iter=1"
[1] "iter=2"
[1] "iter=3"
Estimating optimal shrinkage intensity lambda (correlation matrix): 0.0585

Estimate (local) false discovery rates (partial correlations):
Step 1... determine cutoff point
Step 2... estimate parameters of null distribution and eta0
Step 3... compute p-values and estimate empirical PDF/CDF
Step 4... compute q-values and local fdr

[1] "iter=1"
```

```
[1] "iter=2"
[1] "iter=3"
Estimating optimal shrinkage intensity lambda (correlation matrix): 0.8327

Estimate (local) false discovery rates (partial correlations):
Step 1... determine cutoff point
Step 2... estimate parameters of null distribution and eta0
Step 3... compute p-values and estimate empirical PDF/CDF
Step 4... compute q-values and local fdr

[1] "iter=1"
[1] "iter=2"
[1] "iter=3"
Estimating optimal shrinkage intensity lambda (correlation matrix): 0.7051

Estimate (local) false discovery rates (partial correlations):
Step 1... determine cutoff point
Step 2... estimate parameters of null distribution and eta0
Step 3... compute p-values and estimate empirical PDF/CDF
Step 4... compute q-values and local fdr

[1] "iter=1"
[1] "iter=2"
[1] "iter=3"
Estimating optimal shrinkage intensity lambda (correlation matrix): 0.54

Estimate (local) false discovery rates (partial correlations):
Step 1... determine cutoff point
Step 2... estimate parameters of null distribution and eta0
Step 3... compute p-values and estimate empirical PDF/CDF
Step 4... compute q-values and local fdr

[1] "iter=1"
[1] "iter=2"
[1] "iter=3"
Estimating optimal shrinkage intensity lambda (correlation matrix): 0.3524

Estimate (local) false discovery rates (partial correlations):
Step 1... determine cutoff point
Step 2... estimate parameters of null distribution and eta0
Step 3... compute p-values and estimate empirical PDF/CDF
Step 4... compute q-values and local fdr

[1] "iter=1"
[1] "iter=2"
[1] "iter=3"
Estimating optimal shrinkage intensity lambda (correlation matrix): 0.1896

Estimate (local) false discovery rates (partial correlations):
Step 1... determine cutoff point
Step 2... estimate parameters of null distribution and eta0
Step 3... compute p-values and estimate empirical PDF/CDF
Step 4... compute q-values and local fdr

[1] "iter=1"
[1] "iter=2"
[1] "iter=3"
Estimating optimal shrinkage intensity lambda (correlation matrix): 0.101
```

```
Estimate (local) false discovery rates (partial correlations):
Step 1... determine cutoff point
Step 2... estimate parameters of null distribution and eta0
Step 3... compute p-values and estimate empirical PDF/CDF
Step 4... compute q-values and local fdr

[1] "iter=1"
[1] "iter=2"
[1] "iter=3"
Estimating optimal shrinkage intensity lambda (correlation matrix): 0.8826

Estimate (local) false discovery rates (partial correlations):
Step 1... determine cutoff point
Step 2... estimate parameters of null distribution and eta0
Step 3... compute p-values and estimate empirical PDF/CDF
Step 4... compute q-values and local fdr

[1] "iter=1"
[1] "iter=2"
[1] "iter=3"
Estimating optimal shrinkage intensity lambda (correlation matrix): 0.8666

Estimate (local) false discovery rates (partial correlations):
Step 1... determine cutoff point
Step 2... estimate parameters of null distribution and eta0
Step 3... compute p-values and estimate empirical PDF/CDF
Step 4... compute q-values and local fdr

[1] "iter=1"
[1] "iter=2"
[1] "iter=3"
Estimating optimal shrinkage intensity lambda (correlation matrix): 0.7348

Estimate (local) false discovery rates (partial correlations):
Step 1... determine cutoff point
Step 2... estimate parameters of null distribution and eta0
Step 3... compute p-values and estimate empirical PDF/CDF
Step 4... compute q-values and local fdr

[1] "iter=1"
[1] "iter=2"
[1] "iter=3"
Estimating optimal shrinkage intensity lambda (correlation matrix): 0.624

Estimate (local) false discovery rates (partial correlations):
Step 1... determine cutoff point
Step 2... estimate parameters of null distribution and eta0
Step 3... compute p-values and estimate empirical PDF/CDF
Step 4... compute q-values and local fdr

[1] "iter=1"
[1] "iter=2"
[1] "iter=3"
Estimating optimal shrinkage intensity lambda (correlation matrix): 0.3679

Estimate (local) false discovery rates (partial correlations):
Step 1... determine cutoff point
Step 2... estimate parameters of null distribution and eta0
Step 3... compute p-values and estimate empirical PDF/CDF
```

```
Step 4... compute q-values and local fdr

[1] "iter=1"
[1] "iter=2"
[1] "iter=3"
Estimating optimal shrinkage intensity lambda (correlation matrix): 0.234

Estimate (local) false discovery rates (partial correlations):
Step 1... determine cutoff point
Step 2... estimate parameters of null distribution and eta0
Step 3... compute p-values and estimate empirical PDF/CDF
Step 4... compute q-values and local fdr

[1] "iter=1"
[1] "iter=2"
[1] "iter=3"
```

Compare Network Engines

Jeffrey D. Allen

December 19, 2011

Now we'll want to combine the output from Aracne and add them into the aggregated files from the other methods.

The outputs from aracne are available in ../../Results/aracneConverted/*

We will first construct a list of names for the various networks. These names represent the different categories of networks we'll be testing. For instance, we simulated a "tiny" network with only a few genes, and a "large" network with hundreds of genes, etc.

```
> networkSizes = c("tiny", "small", "moderate", "middle", "large",
+     "huge")
```

For each of these networks, we simulated different experiments which varied in the number of samples they contained. This will test the ability to extract meaning from various numbers of samples from networks of various sizes.

```
> sampleCounts = c(20, 50, 100, 200, 500, 1000)
```

Next we'll convert these names and sample sizes into the names of the files:

```
> files = matrix(nrow = length(networkSizes), ncol = length(sampleCounts))
> for (i in 1:length(networkSizes)) {
+     files[i, ] = paste(networkSizes[i], " nSamp", sampleCounts,
+         ".csv", sep = "")
+ }
> files
```

```
     [,1]                  [,2]                  [,3]
[1,] "tiny nSamp20.csv"     "tiny nSamp50.csv"     "tiny nSamp100.csv"
[2,] "small nSamp20.csv"    "small nSamp50.csv"    "small nSamp100.csv"
[3,] "moderate nSamp20.csv" "moderate nSamp50.csv" "moderate nSamp100.csv"
[4,] "middle nSamp20.csv"   "middle nSamp50.csv"   "middle nSamp100.csv"
[5,] "large nSamp20.csv"    "large nSamp50.csv"    "large nSamp100.csv"
[6,] "huge nSamp20.csv"     "huge nSamp50.csv"     "huge nSamp100.csv"
     [,4]                    [,5]                    [,6]
[1,] "tiny nSamp200.csv"     "tiny nSamp500.csv"     "tiny nSamp1000.csv"
[2,] "small nSamp200.csv"    "small nSamp500.csv"    "small nSamp1000.csv"
[3,] "moderate nSamp200.csv" "moderate nSamp500.csv" "moderate nSamp1000.csv"
[4,] "middle nSamp200.csv"   "middle nSamp500.csv"   "middle nSamp1000.csv"
[5,] "large nSamp200.csv"    "large nSamp500.csv"    "large nSamp1000.csv"
[6,] "huge nSamp200.csv"     "huge nSamp500.csv"     "huge nSamp1000.csv"
```

So the results of Aracne are available in one directory:

```
> aracneFiles <- matrix(paste("../../Results/aracneConverted/",
+     files, ".aracne.out.csv", sep = ""), nrow = 6)
```

And the previously aggregated results are in another:

```
> aggFiles <- paste("../../Results/newAgg", networkSizes, ".csv",
+     sep = "")
```

So we can iterate through the network sizes and add in each of the columns from aracne. Unortunately, the previous results hadn't been sorted. So we'll want to sort both results before combining to ensure that the rows line up.

```
> for (siz in 1:length(networkSizes)) {
+     agg <- read.csv(aggFiles[siz], header = T, row.names = NULL)
+     agg[which(agg[, 1] > agg[, 2]), 1:2] <- agg[which(agg[, 1] >
+         agg[, 2]), 2:1]
+     aggSorted <- agg[order(agg$Source, agg$Target), ]
+     rm(agg)
+     newAgg <- aggSorted[, 1:3]
+     numMethods <- 4
+     oldAggIndex <- 4
+     newAggIndex <- 4
+     sampleIndex <- 1
+     for (sample in 1:length(sampleCounts)) {
+         newAgg[, newAggIndex:(newAggIndex + numMethods - 1)] <- aggSorted[,
+             oldAggIndex:(oldAggIndex + numMethods - 1)]
+         aracne <- read.csv(aracneFiles[siz, sample], header = TRUE,
+             row.names = NULL)
+         aracne[which(aracne[, 1] > aracne[, 2]), 1:2] <- aracne[which(aracne[,
+             1] > aracne[, 2]), 2:1]
+         sortedAracne <- aracne[order(aracne$gene1, aracne$gene2),
+             ]
+         newAgg[, newAggIndex + numMethods] <- sortedAracne$edge
+         colnames(newAgg)[newAggIndex + numMethods] <- paste("Aracne.",
+             sampleCounts[sample], sep = "")
+         sampleIndex <- sampleIndex
+         newAggIndex <- newAggIndex + numMethods + 1
+         oldAggIndex <- oldAggIndex + numMethods
+     }
+     print(paste("Writing CSV for", networkSizes[siz], "network..."))
+     write.csv(newAgg, file = paste("../../Results/aracneAggregate.",
+         networkSizes[siz], ".csv", sep = ""), row.names = FALSE)
+     print(paste("Done at", date()))
+ }
```

```
[1] "Writing CSV for tiny network..."
[1] "Done at Mon Dec 19 15:27:09 2011"
[1] "Writing CSV for small network..."
[1] "Done at Mon Dec 19 15:27:10 2011"
[1] "Writing CSV for moderate network..."
[1] "Done at Mon Dec 19 15:27:10 2011"
[1] "Writing CSV for middle network..."
[1] "Done at Mon Dec 19 15:27:14 2011"
[1] "Writing CSV for large network..."
[1] "Done at Mon Dec 19 15:27:44 2011"
[1] "Writing CSV for huge network..."
[1] "Done at Mon Dec 19 15:30:58 2011"
```

AUC and pAUC Calculations

Jeffrey D. Allen

August 24, 2011

This paper will document the process of generating p/AUC graphs based on the aggreagate files already created. These will graphically and quantitatively represent the performance of various algorithms of constructing gene networks.

1 Input Files

We will first construct a list of names for the various networks. These names represent the different categories of networks we'll be testing. For instance, we simulated a "tiny" network with only a few genes, and a "large" network with hundreds of genes, etc. We have files named in one nomenclature but want to use a different naming convention for the output formats.

```
> networkSizes = c("tiny", "small", "moderate", "middle", "large",
+     "huge")
> outputSizes = c("17 Gene Network", "44 Gene Network", "83 Gene Network",
+     "231 Gene Network", "612 Gene Network", "1344 Gene Network")
```

For each of these networks, we simulated different experiments which varied in the number of samples they contained. This will test the ability to extract meaning from various numbers of samples from networks of various sizes.

```
> sampleCounts = c(20, 50, 100, 200, 500, 1000)
```

We'll then need to be able to read in the aggregate files:

```
> getAgg <- function(sizeID) {
+     fileName <- paste("../../Results/aracneAggregate.", networkSizes[sizeID],
+         ".csv", sep = "")
+     fileName
+     agg <- read.csv(fileName)
+     agg[, 3][which(agg[, 3] > 0)] = 1
+     agg
+ }
```

We can now begin to do some analysis on each table.

2 ROCs

We'll first graphically represent the ROC curves for each method and/or network size.

```
> library(ROCR)
> getROC <- function(truth, predicted) {
+     pred <- prediction(predicted, truth)
+     perf <- performance(pred, "sens", "spec")
+     perf
+ }
> calculateNetworkROCs <- function(networkSize) {
+     agg <- getAgg(networkSize)
+     truth <- agg[, 3]
+     par(mfrow = c(6, 5), mar = c(4, 4, 3, 1))
```

```
+     for (i in 4:length(agg[1, ])) {
+         pred <- agg[, i]
+         if (!is.na(sum(pred))) {
+             roc <- getROC(truth, pred)
+             plot(roc, main = colnames(agg)[i])
+         }
+         else {
+             plot(0, 0, main = colnames(agg[i]))
+         }
+     }
+ }
> calculateNetworkROCs(1)
```

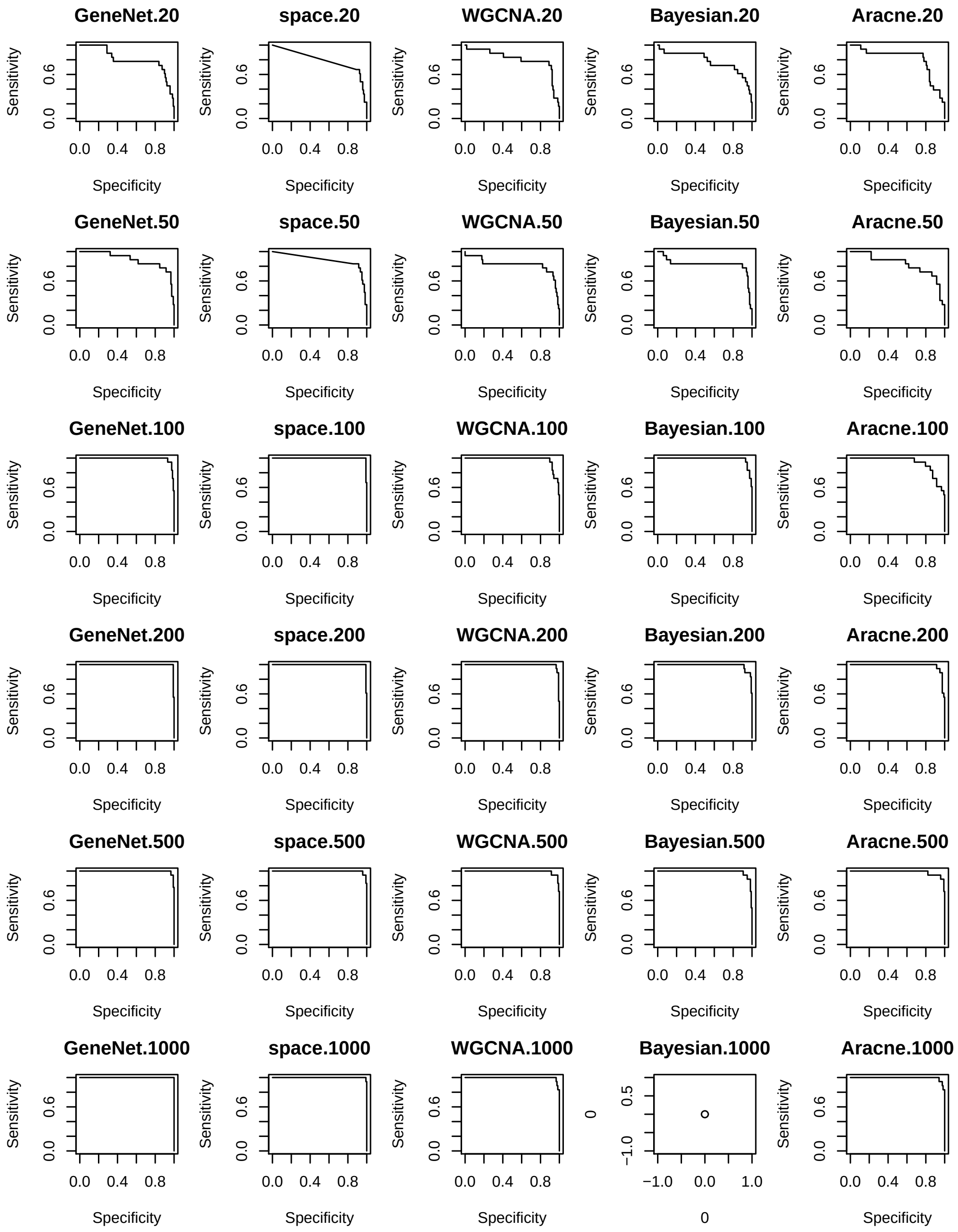

3 AUCs

We'll first want to calculate a matrix of AUCs to quantify the performance of each method. We can compute a matrix which is setup to allow for easy analysis of either method-based or network-based performance.

```
> library(ROCR)
> getAUC <- function(truth, predicted) {
+     pred <- prediction(predicted, truth)
+     auc <- performance(pred, "auc")@y.values[[1]]
+     auc
+ }
> AUCsToMatrix <- function(AUC) {
+     nSamples <- length(sampleCounts)
+     nMethods <- length(AUC)/nSamples
+     if (as.integer(nMethods) != nMethods) {
+         return
+     }
+     AUCs <- matrix(nrow = nSamples, ncol = nMethods)
+     for (i in 1:length(AUC)) {
+         method <- i%%nMethods
+         if (method == 0) {
+             method = nMethods
+         }
+         AUCs[(i - 1)%/%nMethods + 1, method] <- AUC[i]
+     }
+     methodNames <- array()
+     for (i in 1:length(AUCs[1, ])) {
+         methodNames[i] <- strsplit(names(AUC)[i], ".", fixed = TRUE)[[1]][1]
+     }
+     colnames(AUCs) <- methodNames
+     rownames(AUCs) = sampleCounts
+     AUCs
+ }
> calculateNetworkAUCs <- function(networkSize) {
+     agg <- getAgg(networkSize)
+     truth <- agg[, 3]
+     AUCs <- array()
+     for (i in 4:length(agg[1, ])) {
+         pred <- agg[, i]
+         if (!is.na(sum(pred))) {
+             auc <- getAUC(truth, pred)
+         }
+         else {
+             auc <- NA
+         }
+         AUCs[i] <- auc
+     }
+     AUCs <- AUCs[4:length(AUCs)]
+     names(AUCs) <- colnames(agg[4:length(agg[1, ])])
+     AUCsToMatrix(AUCs)
+ }
> calculateNetworkAUCs(1)
```

```
       GeneNet     space     WGCNA  Bayesian    Aracne
20   0.8060264 0.7937853 0.8093220 0.7791902 0.8093220
50   0.8912429 0.8825330 0.8187382 0.8234463 0.8248588
100  0.9901130 0.9971751 0.9764595 0.9868173 0.9364407
200  0.9962335 0.9967043 0.9934087 0.9882298 0.9849341
500  0.9967043 0.9967043 0.9924670 0.9872881 0.9863465
1000 1.0000000 0.9995292 0.9957627        NA 0.9943503
```

This matrix can then be plotted in a variety of ways to emphasize performance differences from one method to the next. Or, to plot them everything on one graph per network size:

```
> plotAUC <- function(AUCs, netsize, pAUC = FALSE, ylimitsOrig = NULL) {
+     if (is.null(ylimitsOrig)) {
```

```
+         minY = min(AUCs[which(!is.na(AUCs))])
+         maxY = max(AUCs[which(!is.na(AUCs))])
+         ylimits = c(minY, maxY)
+     }
+     else {
+         ylimits <- ylimitsOrig
+     }
+     if (pAUC) {
+         yLab = "pAUC"
+     }
+     else {
+         yLab = "AUC"
+     }
+     plot(sampleCounts, AUCs[, 1], main = outputSizes[netsize],
+         type = "b", col = 1, pch = 1, ylim = ylimits, xlab = "Sample Size",
+         ylab = yLab, log = "x")
+     for (method in 2:length(AUCs[1, ])) {
+         lines(sampleCounts, AUCs[, method], col = method, pch = method,
+             type = "b", lty = method)
+     }
+     isAllNA <- function(x) {
+         all(is.na(x))
+     }
+     legend(170, mean(ylimits), colnames(AUCs)[!apply(AUCs, 2,
+         isAllNA)], col = which(!apply(AUCs, 2, isAllNA)), lty = which(!apply(AUCs,
+         2, isAllNA)), bty = "n", pch = which(!apply(AUCs, 2,
+         isAllNA)))
+ }
> plotSingle <- function(netsize, pAUC = FALSE, ylimitsOrig = NULL) {
+     if (pAUC) {
+         AUCs <- calculateNetworkpAUCs(netsize)
+     }
+     else {
+         AUCs <- calculateNetworkAUCs(netsize)
+     }
+     plotAUC(AUCs, netsize, pAUC, ylimitsOrig)
+ }
> plotAUCs <- function(pAUC = FALSE, ylimits = NULL) {
+     ylimitsOrig <- ylimits
+     par(mfrow = c(3, 2), mar = c(4, 4, 3, 1))
+     for (netsize in 1:length(networkSizes)) {
+         plotSingle(netsize, pAUC, ylimits)
+     }
+ }
> plotAUCs(FALSE)
```

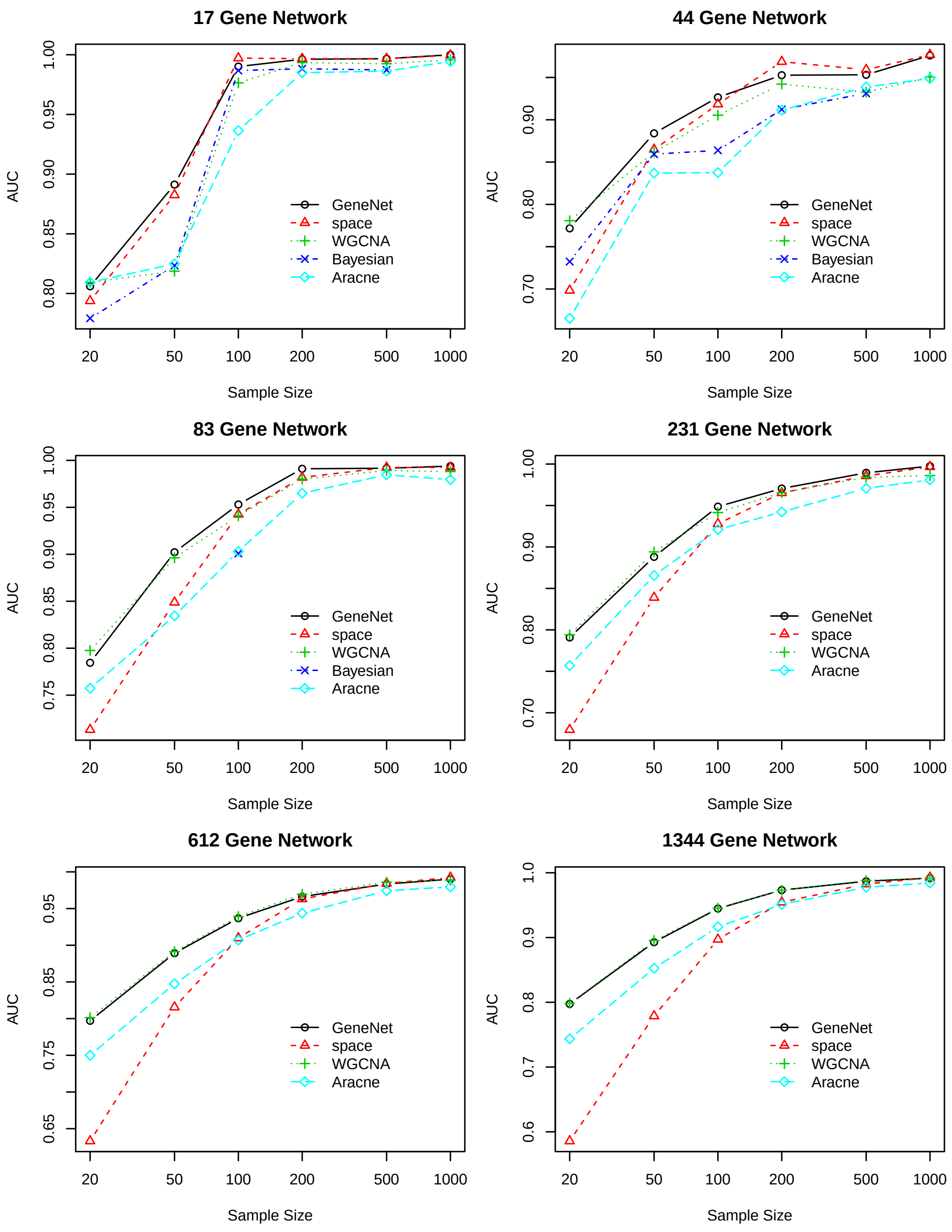

4 pAUCs

Finally, we'll want to analyze the pAUCs for each method.

```
> getpAUC <- function(truth, predicted, threshold = 0.005) {
+     pred <- prediction(predicted, truth)
+     auc <- performance(pred, "auc", fpr.stop = threshold)@y.values[[1]]
+     auc
+ }
> calculateNetworkpAUCs <- function(networkSize) {
+     agg <- getAgg(networkSize)
+     truth <- agg[, 3]
+     pAUCs <- array()
+     for (i in 4:length(agg[1, ])) {
+         pred <- agg[, i]
+         if (!is.na(sum(pred))) {
+             pauc <- getpAUC(truth, pred)
```

```
+         }
+         else {
+             pauc <- NA
+         }
+         pAUCs[i] <- pauc
+     }
+     pAUCs <- pAUCs[4:length(pAUCs)]
+     names(pAUCs) <- colnames(agg[4:length(agg[1, ])])
+     AUCsToMatrix(pAUCs)
+ }
> plotAUCs(TRUE, ylim = NULL)
```

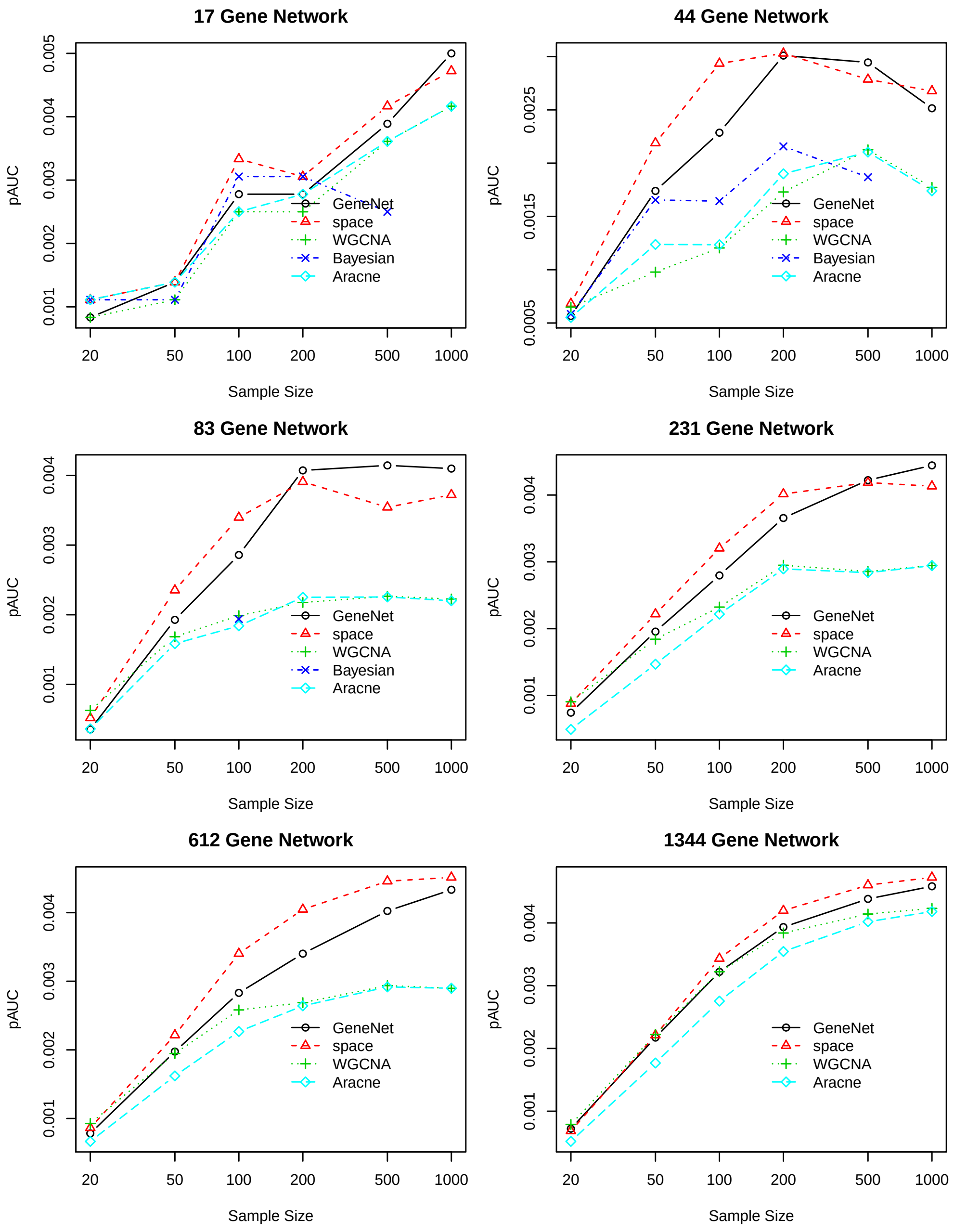

5 ROC Curves

There are a couple of additional ROC curves which we may want to include in the manuscript. Let's define the function for plotting them.

```
> combineROCs <- function(networkSize, sampleSize) {
+     agg <- getAgg(networkSize)
+     truth <- agg[, 3]
+     nSamples <- length(sampleCounts)
+     nMethods <- (dim(agg)[2] - 3)/nSamples
+     methodNames <- c("GeneNet", "Space", "WGCNA", "Bayesian",
+         "Aracne")
+     nonEmptyMethods <- array()
+     colInd <- 1
+     for (i in (4 + ((sampleSize - 1) * nMethods)):(3 + ((sampleSize) *
+         nMethods))) {
+         pred <- agg[, i]
+         if (!all(is.na(pred))) {
+             nonEmptyMethods <- c(nonEmptyMethods, colInd)
+             roc <- getROC(truth, pred)
+             if (i == 4 + ((sampleSize - 1) * nMethods)) {
+                 plot(roc, main = paste(outputSizes[networkSize],
+                   "With", sampleCounts[sampleSize], "Samples"))
+             }
+             else {
+                 plot(roc, add = TRUE, col = colInd, lty = colInd)
+             }
+         }
+         colInd <- colInd + 1
+     }
+     legend(0, 0.4, legend = methodNames[nonEmptyMethods], col = nonEmptyMethods,
+         lty = nonEmptyMethods, bty = "n")
+ }
```

And we'll plot a couple of them here.

```
> combineROCs(5, 5)
```

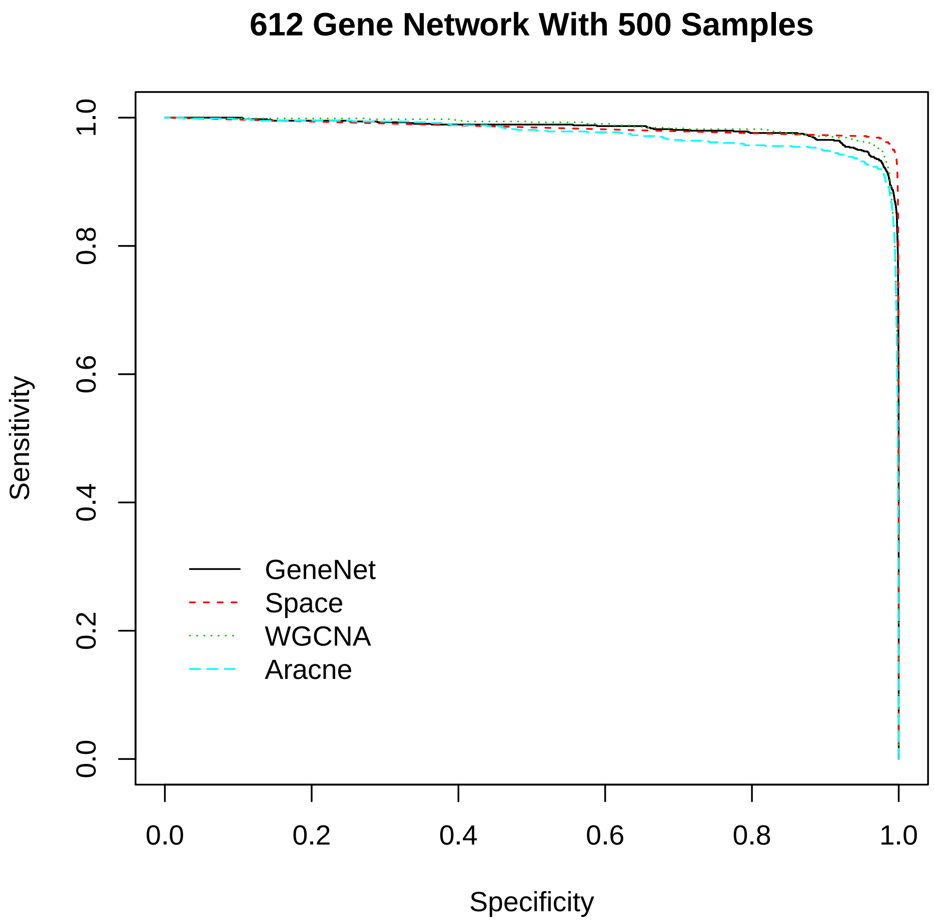


```
> combineROCs(5, 6)
```

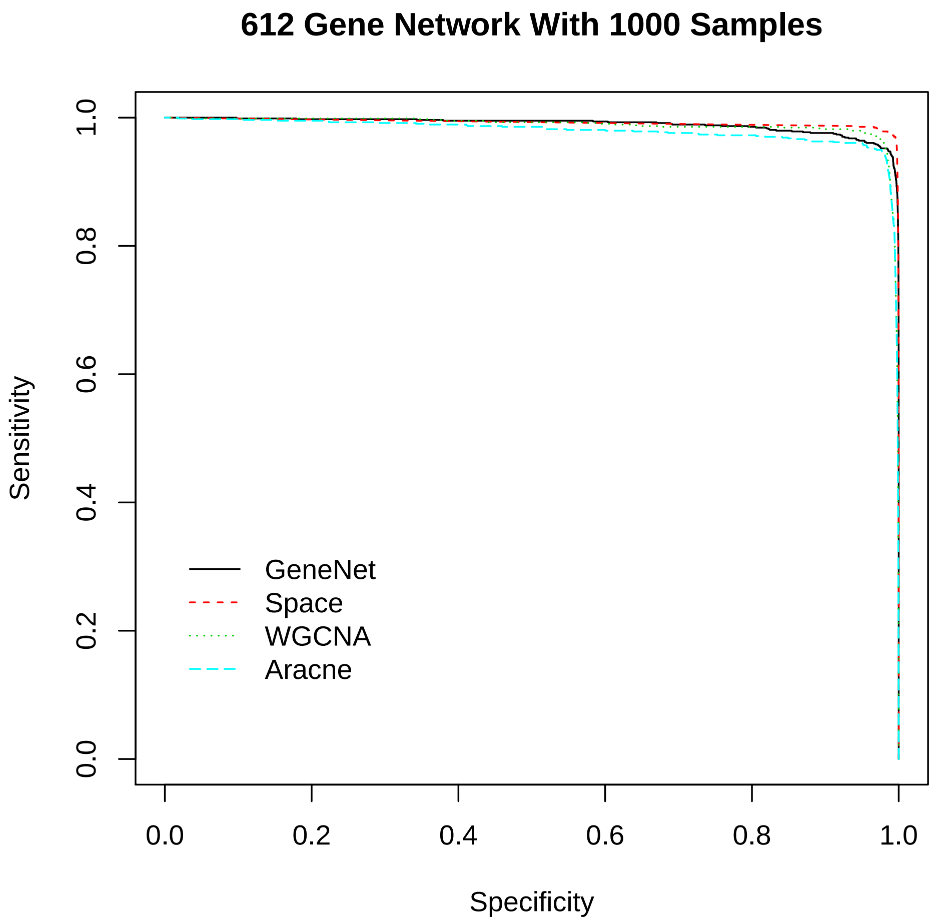


```
> combineROCs(3, 1)
```

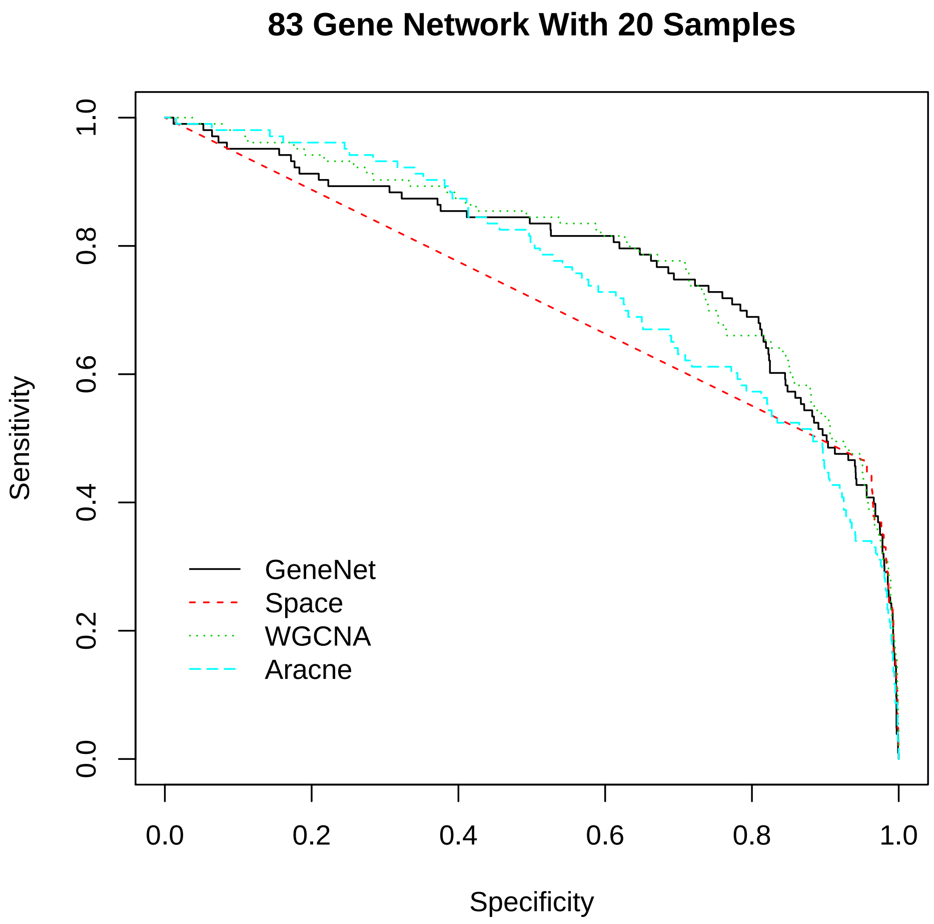

83 Gene Network With 20 Samples
Sensitivity
Specificity
0.0
0.2
0.4
0.6
0.8
1.0
GeneNet
Space
WGCNA
Aracne

Connectivity

Jeffrey D. Allen

September 1, 2011

This document will explore the conectivity scores of each method as an attempt to explain why GeneNet would have such strange performance in some settings.

We will first construct a list of names for the various networks. These names represent the different categories of networks we'll be testing. For instance, we simulated a "tiny" network with only a few genes, and a "large" network with hundreds of genes, etc.

```
> networkSizes = c("tiny", "small", "moderate", "middle", "large",
+     "huge")
> outputSizes = c("17 Gene Network", "44 Gene Network", "83 Gene Network",
+     "231 Gene Network", "612 Gene Network", "1344 Gene Network")
```

For each of these networks, we simulated different experiments which varied in the number of samples they contained. This will test the ability to extract meaning from various numbers of samples from networks of various sizes.

```
> sampleCounts = c(20, 50, 100, 200, 500, 1000)
```

We'll then need to be able to read in the aggregate files:

```
> getAgg <- function(sizeID) {
+     fileName <- paste("../../Results/aracneAggregate.", networkSizes[sizeID],
+         ".csv", sep = "")
+     fileName
+     agg <- read.csv(fileName)
+     agg[, 3][which(agg[, 3] > 0)] = 1
+     agg
+ }
```

We'll also want to be able to calculate the connectivity scores. We can use the function from a previous file.

```
> geneConnectivity <- function(weights) {
+     connect <- list()
+     for (i in 1:length(weights[, 1])) {
+         a <- paste(weights[i, 1])
+         if (is.null(connect[a][[1]])) {
+             connect[a] = 0
+         }
+         connect[a][[1]] <- connect[a][[1]] + weights[i, 3][[1]]
+         b <- paste(weights[i, 2])
+         if (is.null(connect[b][[1]])) {
+             connect[b] = 0
+         }
+         connect[b][[1]] <- connect[b][[1]] + weights[i, 3][[1]]
+     }
+     connect
+ }
```

This function merely returns a list who's indexes are gene names and values are the sum of the weights of all connections affiliated with that gene.

This code could be used like the following example:

```
> agg <- getAgg(1)
> weights <- cbind(agg$Source, agg$Target, agg$GeneNet.20)
> SG <- geneConnectivity(weights)
> SG["983"]

$`983`
[1] 0.5915871
```

where the output of this example is the connectivity value for gene named "983".

Now we have a way to get the relative connectivity values of each gene, but we probably want to normalize these connectivity scores to [0, 1]. That can be done with this function (and probably another, built-in R function).

```
> normalize <- function(connList) {
+     w <- array()
+     for (i in 1:length(connList)) {
+         w[i] = connList[i][[1]]
+     }
+     range <- max(w)
+     for (i in 1:length(connList)) {
+         connList[i][[1]] = (connList[i][[1]])/range
+     }
+     connList
+ }
```

which maintains the list structure, but manipulates the inner values to be on the interval from [0, 1].

At this point, we have a way of scoring the connectivity of a gene in a network and then normalizing that value to more easily compare.

A loop to automatically iterate over all algorithms/sample sizes and calculate their connectivity could be something like:

```
> getConnectivity <- function(agg) {
+     allConn <- list()
+     for (i in 3:length(agg[1, ])) {
+         weights <- cbind(agg$Source, agg$Target, agg[, i])
+         conn <- geneConnectivity(weights)
+         conn <- normalize(conn)
+         allConn <- cbind(allConn, conn)
+     }
+     colnames(allConn) <- colnames(agg)[3:length(colnames(agg))]
+     allConn
+ }
> agg <- getAgg(1)
> head(getConnectivity(agg))

     Truth GeneNet.20 space.20  WGCNA.20  Bayesian.20 Aracne.20 GeneNet.50
472  0.25  0.8811262  0.6705645 0.1734738 0.7076971   0.7526778 0.7914055
545  0.25  0.8567562  0.4968285 0.1558933 0.6857737   0.7502365 0.7229768
983  0.375 0.8080925  0.1225448 0.1144906 0.681627    0.7501622 0.8717003
994  0.125 0.8886913  0.3859765 0.2396461 0.757357    0.848961  0.7554396
995  0.5   0.9452072  0.7775094 0.5771888 0.8419106   0.8920296 0.8697318
1111 0.25  0.8942557  0.495461  0.3986875 0.6523923   0.7131561 0.6763424
     space.50   WGCNA.50    Bayesian.50 Aracne.50 GeneNet.100 space.100
472  0.3677458  0.1433712   0.5885838   0.5588126 0.6689395   0.236538
545  0.07902036 0.003170287 0.3776225   0.3880256 0.6822972   0.3671312
983  0.7486836  0.390452    0.760737    0.7280885 0.677316    0.5153037
994  0.3872335  0.2473196   0.5299868   0.6828324 0.4640572   0.3460195
995  0.6002012  0.24985     0.7728874   0.844906  0.7062701   0.7482974
1111 0.3676074  0.09279469  0.5783049   0.626861  0.6106462   0.3817132
     WGCNA.100   Bayesian.100 Aracne.100 GeneNet.200 space.200 WGCNA.200
472  0.003328825 0.5307075    0.6332917  0.6088121   0.3651845 0.02398479
```

```
545  0.06061028  0.5847749    0.8431643  0.5139475   0.3094697 0.01400261
983  0.2380868   0.617707     0.7706217  0.6916747   0.5991716 0.2650986
994  0.230229    0.3673716    0.7151573  0.4125488   0.2704897 0.2491862
995  0.2392317   0.7500644    0.909632   0.6779378   0.6401618 0.2792852
1111 0.1602934   0.4765163    0.8225392  0.5637988   0.3868741 0.2367471
     Bayesian.200 Aracne.200 GeneNet.500 space.500 WGCNA.500  Bayesian.500
472  0.4147941    0.6664142  0.5547175   0.3704464 0.01327958 0.4557646
545  0.4514007    0.6052482  0.5123467   0.3194773 0.01805808 0.4480627
983  0.4436392    0.9109205  0.5565467   0.4984423 0.2336173  0.5077054
994  0.2455246    0.6990053  0.4303646   0.2918493 0.1932996  0.3183501
995  0.4404188    0.9600731  0.6767985   0.6678099 0.2249516  0.820655
1111 0.4047758    0.7843673  0.4867411   0.4036448 0.1688589  0.3573911
     Aracne.500 GeneNet.1000 space.1000 WGCNA.1000 Bayesian.1000 Aracne.1000
472  0.5203769  0.6310549    0.5048351  0.00675345 NA            0.4436776
545  0.5148911  0.5795136    0.4838187  0.01221472 NA            0.4402564
983  0.921516   0.4501239    0.4198186  0.3643962  NA            0.9199814
994  0.6854301  0.3403829    0.2660181  0.350345   NA            0.7219233
995  1          0.6618076    0.6654302  0.1814409  NA            0.9404364
1111 0.7230308  0.467685     0.3798845  0.1342415  NA            0.6932528
```

which produces all of the connectivity values for the tiny network.

We can now begin to do some analysis on each table.

We'll need a function that can plot the histogram of a given network size:

```
> plotHists <- function(networkSizeID, sampleSizeID) {
+     par(mfrow = c(3, 2), mar = c(4, 4, 3, 1))
+     agg <- getAgg(networkSizeID)
+     conn <- getConnectivity(agg)
+     nMethods <- (dim(agg)[2] - 3)/length(sampleCounts)
+     startCol <- 2 + (sampleSizeID - 1) * nMethods
+     for (i in c(1, startCol:(startCol + nMethods - 1))) {
+         name <- strsplit(colnames(conn)[i], ".", fixed = "TRUE")[[1]][1]
+         hist(as.numeric(conn[, i]), main = name, xlab = "Connectivity (Normalized)",
+             xlim = 0:1, breaks = seq(0, 1, 0.1))
+     }
+ }
```

Now we can plot a couple of graphs to see the performance:

```
> plotHists(2, 4)
```

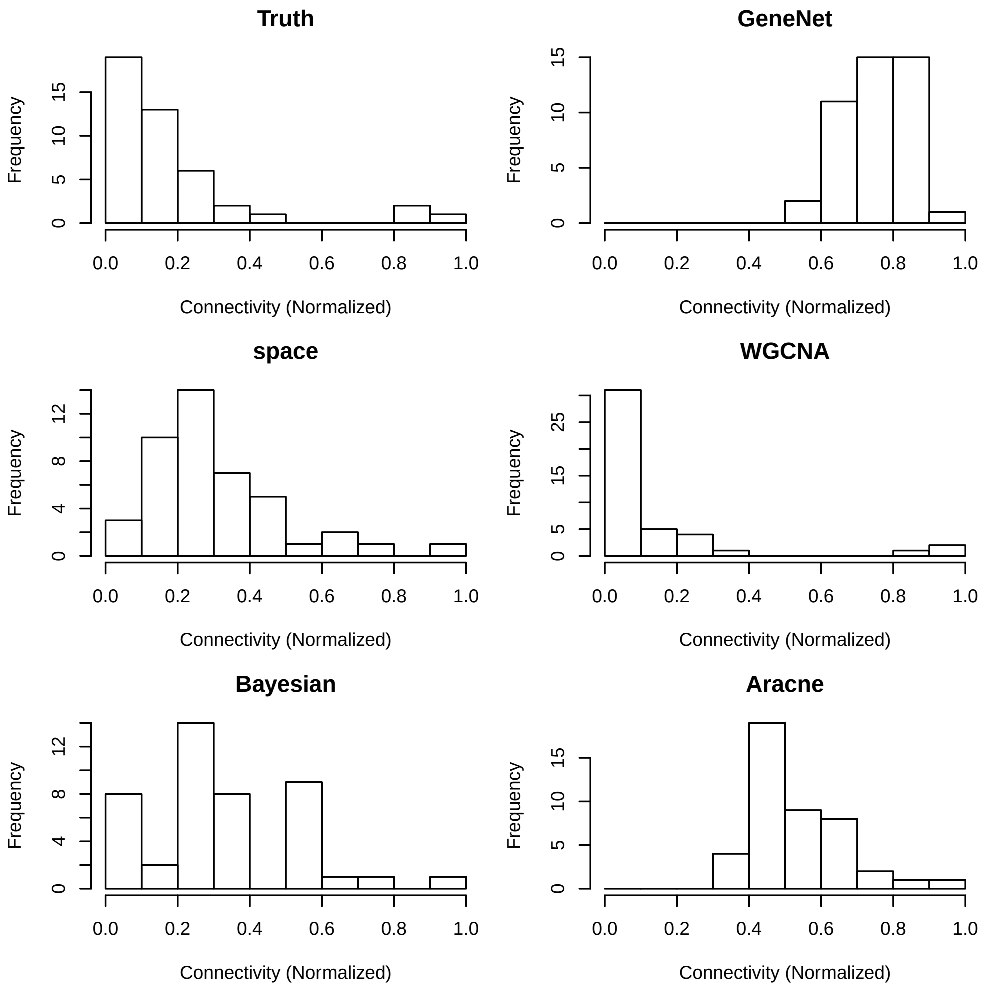

And a larger one:

```
> plotHists(3, 3)
```

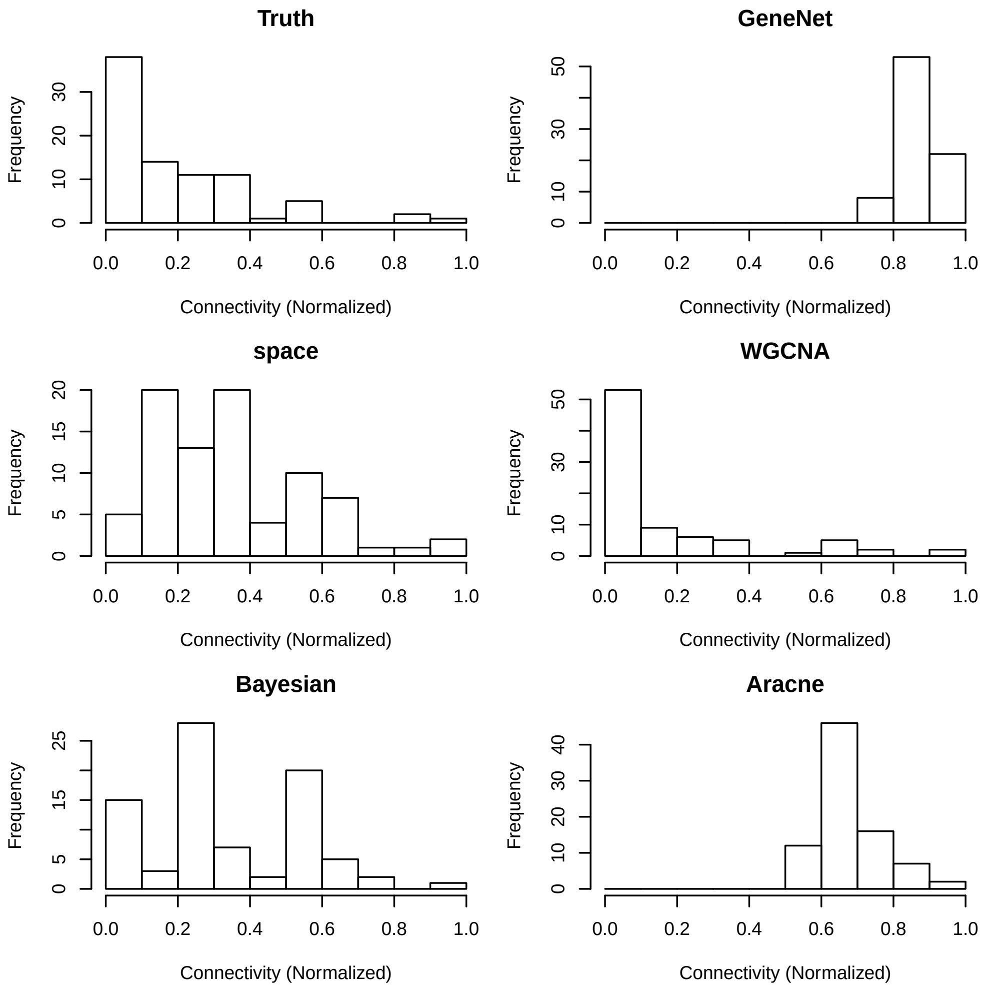
Truth
GeneNet
space
WGCNA
Bayesian
Aracne
Frequency
Connectivity (Normalized)
0.0 0.2 0.4 0.6 0.8 1.0

Hub Gene Calculations

Jeffrey D. Allen

December 18, 2010

This document will build on the previous documentation which constructed aggregate files which were tables describing the output of various gene network construction methods. This file will use those aggregate tables to analyze the performance of each method specifically on hub genes.

We will first construct a list of names for the various networks. These names represent the different categories of networks we'll be testing. For instance, we simulated a "tiny" network with only a few genes, and a "large" network with hundreds of genes, etc.

```
> networkSizes = c("tiny", "small", "moderate", "middle", "large",
+     "huge")
> outputSizes = c("17 Gene Network", "44 Gene Network", "83 Gene Network",
+     "231 Gene Network", "612 Gene Network", "1344 Gene Network")
```

For each of these networks, we simulated different experiments which varied in the number of samples they contained. This will test the ability to extract meaning from various numbers of samples from networks of various sizes.

```
> sampleCounts = c(20, 50, 100, 200, 500, 1000)
```

We'll then need to be able to read in the aggregate files:

```
> getAgg <- function(sizeID) {
+     fileName <- paste("../../Results/newAgg", networkSizes[sizeID],
+         ".csv", sep = "")
+     fileName
+     agg <- read.csv(fileName)
+     agg[, 3][which(agg[, 3] > 0)] = 1
+     agg
+ }
```

We can now begin to do some analysis on each table.

The principle behind the idea of hub-genes is that we're seeking very highly-connected genes. Intuitively, this is most easily accomplished by analyzing a sparse, binary adjacency matrix and finding those genes which have an abnormally high number of connections. However, some of the methods tested in creating the aggregate tables produce very non-sparse matrices with weighted connections. This presents a problem in that, in some cases, nearly all possible connections are non-zero, so we'll need to consider the weight in calculating the "connectivity" of a gene.

Because this is an undirected graph, a connection between A and B will increase the connectivity scores of both A and B. Thus, we can loop through all of the connections in a produced network and keep a running sum of the weights of the connections for each gene.

```
> geneConnectivity <- function(weights) {
+     connect <- list()
+     for (i in 1:length(weights[, 1])) {
+         a <- paste(weights[i, 1])
+         if (is.null(connect[a][[1]])) {
+             connect[a] = 0
+         }
+         connect[a][[1]] <- connect[a][[1]] + weights[i, 3][[1]]
+         b <- paste(weights[i, 2])
+         if (is.null(connect[b][[1]])) {
```

```
+             connect[b] = 0
+         }
+         connect[b][[1]] <- connect[b][[1]] + weights[i, 3][[1]]
+     }
+     connect
+ }
```

This function merely returns a list who's indexes are gene names and values are the sum of the weights of all connections affiliated with that gene.

This code could be used like the following example:

```
> agg <- getAgg(1)
> weights <- cbind(agg$Source, agg$Target, agg$GeneNet.20)
> SG <- geneConnectivity(weights)
> SG["983"]

$`983`
[1] 0.5915871
```

where the output of this example is the connectivity value for gene named "983".

Now we have a way to get the relative connectivity values of each gene, but we probably want to normalize these connectivity scores to [0, 1]. That can be done with this function (and probably another, built-in R function).

```
> normalize <- function(connList) {
+     w <- array()
+     for (i in 1:length(connList)) {
+         w[i] = connList[i][[1]]
+     }
+     range <- max(w)
+     for (i in 1:length(connList)) {
+         connList[i][[1]] = (connList[i][[1]])/range
+     }
+     connList
+ }
```

which maintains the list structure, but manipulates the inner values to be on the interval from [0, 1].

At this point, we have a way of scoring the connectivity of a gene in a network and then normalizing that value to more easily compare. A loop to automatically iterate over all algorithms/sample sizes and calculate their connectivity could be something like:

```
> getConnectivity <- function(agg) {
+     allConn <- list()
+     for (i in 3:length(agg[1, ])) {
+         weights <- cbind(agg$Source, agg$Target, agg[, i])
+         conn <- geneConnectivity(weights)
+         conn <- normalize(conn)
+         allConn <- cbind(allConn, conn)
+     }
+     colnames(allConn) <- colnames(agg)[3:length(colnames(agg))]
+     allConn
+ }
> agg <- getAgg(1)
> head(getConnectivity(agg))

     Truth GeneNet.20 space.20  WGCNA.20   Bayesian.20 GeneNet.50 space.50
983  0.375 0.8080925  0.1225448 0.1144906  0.681627    0.8717003  0.7486836
1111 0.25  0.8942557  0.495461  0.3986875  0.6523923   0.6763424  0.3676074
472  0.25  0.8811262  0.6705645 0.1734738  0.707697    0.7914055  0.3677458
1460 0.25  0.890325   0.8410155 0.9945949  0.6872572   0.7557967  0.6223033
545  0.25  0.8567562  0.4968285 0.1558933  0.6857737   0.7229768  0.07902036
```

```
2033 0.125 0.6223432  0.1002879 0.02834089 0.5343078   0.7995738  0.2935475
     WGCNA.50    Bayesian.50 GeneNet.100 space.100 WGCNA.100   Bayesian.100
983  0.390452    0.760737    0.677316    0.5153037 0.2380868   0.617707
1111 0.09279469  0.578305    0.6106462   0.3817132 0.1602934   0.4765163
472  0.1433712   0.5885838   0.6689395   0.236538  0.003328825 0.5307075
1460 0.96462     0.5467211   0.6467995   0.6065943 1           0.4524476
545  0.003170287 0.3776225   0.6822972   0.3671312 0.06061028  0.5847749
2033 0.00985677  0.6242806   0.6166012   0.2329421 0.05250699  0.5026825
     GeneNet.200 space.200 WGCNA.200  Bayesian.200 GeneNet.500 space.500
983  0.6916747   0.5991716 0.2650986  0.4436392    0.5565467   0.4984423
1111 0.5637988   0.3868741 0.2367471  0.4047758    0.4867411   0.4036448
472  0.6088121   0.3651845 0.02398479 0.4147941    0.5547175   0.3704464
1460 0.4696661   0.5138428 1          0.374319     0.7097904   0.6414811
545  0.5139475   0.3094697 0.01400261 0.4514007    0.5123467   0.3194773
2033 0.5060332   0.2773567 0.02563786 0.3241087    0.494493    0.2970715
     WGCNA.500   Bayesian.500 GeneNet.1000 space.1000 WGCNA.1000  Bayesian.1000
983  0.2336173   0.5077054    0.4501239    0.4198186  0.3643962   NA
1111 0.1688589   0.3573911    0.467685     0.3798845  0.1342415   NA
472  0.01327958  0.4557646    0.6310549    0.5048351  0.00675345  NA
1460 1           0.4871704    0.7269783    0.6906353  1           NA
545  0.01805808  0.4480627    0.5795136    0.4838187  0.01221472  NA
2033 0.008899569 0.435045     0.4584641    0.3640128  0.005462523 NA
```

which produces all of the connectivity values for the tiny network.

We could also process all of the networks at once and save the output as a series of CSV files.

```
> i <- 1
> agg <- getAgg(i)
> con <- getConnectivity(agg)
> write.csv(con, paste("../../Results/ConnectivityOutput-", outputSizes[i],
+     ".csv", sep = ""))
```

It's much easier to work with the data if we binarize the truth vector regarding which genes actually are or are not hub genes. For this, we'll set a threshold (which could be based on the raw number of connections calculated earlier, or set arbitrarily here) and then round accordingly. We want any gene with 4 or more connections to be considered a hub gene, so we'll need to calculate what the normalized threshold would be.

```
> getThreshold <- function(agg, numGenes = 4) {
+     weights <- agg[, 1:3]
+     rawConn <- geneConnectivity(weights)
+     normConn <- normalize(rawConn)
+     truthThreshold <- min(as.numeric(normConn[which(rawConn >=
+         numGenes)]))
+     truthThreshold
+ }
```

Given this threshold, we can then binarize the true connection vector using code similar to:

```
> agg <- getAgg(1)
> conn <- getConnectivity(agg)
> truthConn <- conn[, 1]
> truthConn <- truthConn >= getThreshold(agg)
> truthConn

  983  1111   472  1460   545  2033  5347  5591   995  1263  1457  9088  7157
FALSE FALSE FALSE FALSE FALSE FALSE FALSE FALSE  TRUE FALSE FALSE FALSE  TRUE
11200   994  6125  4140
 TRUE FALSE FALSE FALSE
```

where truthConn then represents the Boolean value of whether or not the given gene is a hub gene.

We can then create an ROC curve based off of this data for various configurations

```
> library(ROCR)
> calcAUCs <- function(agg, numGenes = 0) {
+     weights <- agg[, 1:3]
+     conn <- getConnectivity(agg)
+     truthConn <- conn[, 1]
+     if (numGenes == 0) {
+         truthConn <- truthConn >= getThreshold(agg)
+     }
+     else {
+         truthConn <- truthConn >= getThreshold(agg, numGenes)
+     }
+     AUCs <- array()
+     for (i in 2:length(conn[1, ])) {
+         if (!all(is.na(conn[, i]))) {
+             pred <- prediction(as.numeric(conn[, i]), as.integer(truthConn))
+             AUC <- performance(pred, "auc")@y.values[[1]]
+             AUCs[i - 1] = AUC
+         }
+         else {
+             AUCs[i - 1] = NA
+         }
+     }
+     names(AUCs) = colnames(conn)[2:length(conn[1, ])]
+     AUCs
+ }
```

which calculates the AUCs of the various methods for the current network.

We can plot these AUCs to show performance of a method over the number of samples.

```
> AUCsToMatrix <- function(AUC) {
+     nSamples <- length(sampleCounts)
+     nMethods <- length(AUC)/nSamples
+     if (as.integer(nMethods) != nMethods) {
+         return
+     }
+     AUCs <- matrix(nrow = nSamples, ncol = nMethods)
+     for (i in 1:length(AUC)) {
+         method <- i%%nMethods
+         if (method == 0) {
+             method = nMethods
+         }
+         AUCs[(i - 1)%/%nMethods + 1, method] <- AUC[i]
+     }
+     methodNames <- array()
+     for (i in 1:length(AUCs[1, ])) {
+         methodNames[i] <- strsplit(names(AUC)[i], ".", fixed = TRUE)[[1]][1]
+     }
+     colnames(AUCs) <- methodNames
+     rownames(AUCs) = sampleCounts
+     AUCs
+ }
> plotHubGeneAUCs <- function(AUCs, title = "AUC of Hub Gene Detection") {
+     AUCs <- AUCsToMatrix(AUCs)
+     plot(NA, NA, ylim = c(0, 1), xlim = c(0, max(sampleCounts)),
+         main = title, xlab = "Num. Samples", ylab = "AUC")
+     x <- sampleCounts
+     for (i in 1:length(AUCs[1, ])) {
+         y <- AUCs[, i]
+         lines(x, y, col = i, pch = i, type = "b", lty = i)
+     }
```

```
+     legend(650, 0.5, colnames(AUCs), col = 1:length(AUCs[1, ]),
+         pch = 1:length(AUCs[1, ]), lty = 1:length(AUCs[1, ]),
+         bty = "o")
+ }
```

Finally, all graphs can be generated as follows, defining a hub gene as a gene with 4 or more connections:

```
> par(mfrow = c(3, 2), mar = c(4, 4, 3, 1))
> for (i in 1:length(networkSizes)) {
+     agg <- getAgg(i)
+     conn <- getConnectivity(agg)
+     plotHubGeneAUCs(calcAUCs(agg, 4), outputSizes[i])
+ }
```

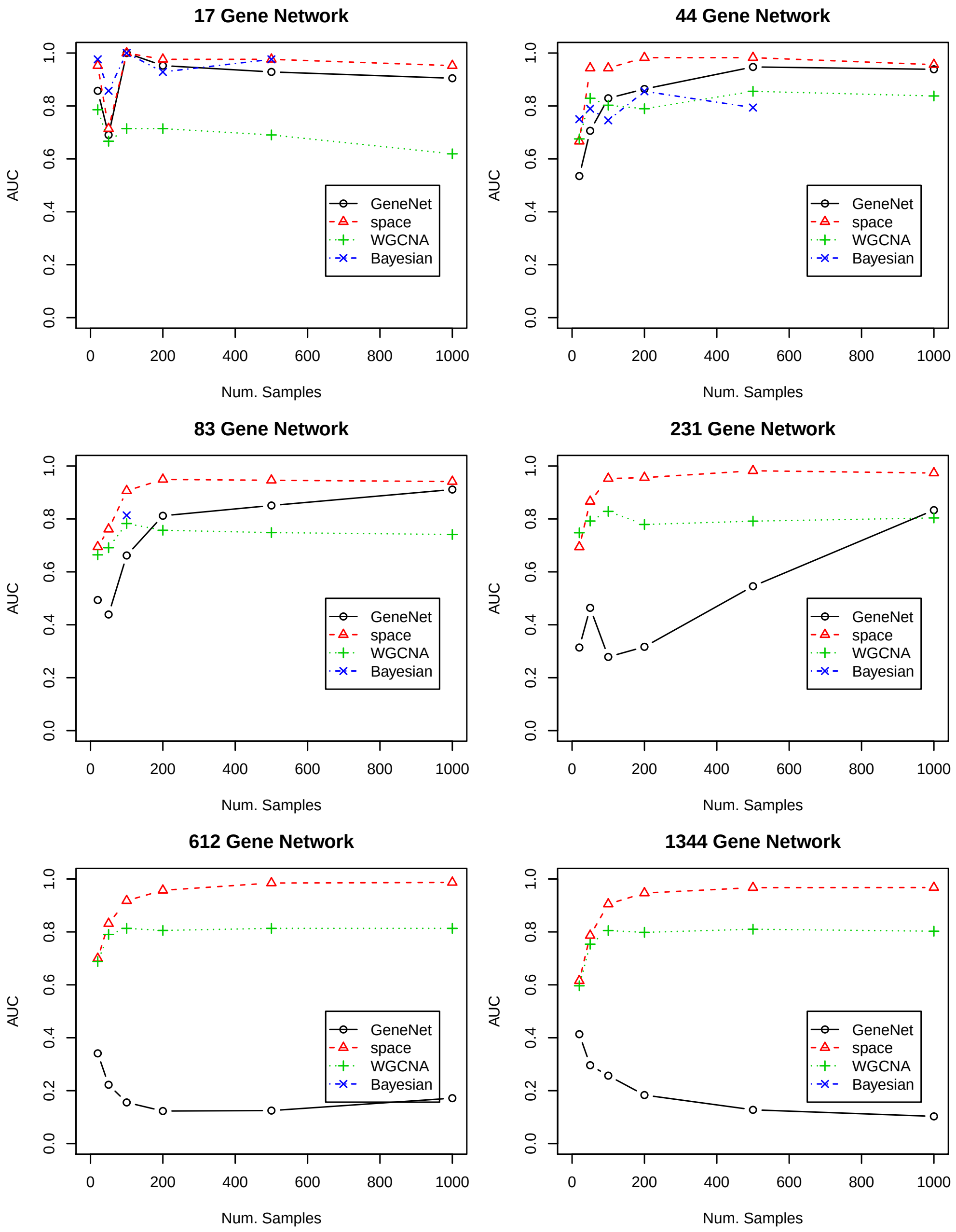

For 5 or more:
And for 6 or more:

Compare Network Engines

Jeffrey D. Allen

July 29, 2011

```
> data <- as.matrix(read.csv("../../Ecoli/Ecoli expression.csv",
+     header = T, row.names = 1))
```

```
Package WGCNA version 1.11-3 loaded.
```

```
> matrixToList <- function(mat, includeGeneIDs = TRUE) {
+     arSize <- length(length(mat)/2 - dim(mat)[1]/2)
+     for (a in 1:dim(mat)[1]) {
+         for (b in 1:dim(mat)[2]) {
+             strength <- max(abs(mat[a, b]), abs(mat[b, a]))
+             mat[min(a, b), max(a, b)] <- strength
+             mat[max(a, b), min(a, b)] <- 0
+             if (a == b) {
+                 mat[a, b] = 0
+             }
+         }
+     }
+     outputPointer <- 1
+     weights <- array(arSize)
+     sources <- array(arSize)
+     targets <- array(arSize)
+     for (a in 1:(dim(mat)[1] - 1)) {
+         start <- outputPointer
+         end <- dim(mat)[2] + outputPointer - a - 1
+         length <- end - start + 1
+         weights[start:end] <- mat[a, (a + 1):dim(mat)[2]]
+         if (includeGeneIDs) {
+             sources[start:end] <- colnames(mat)[a]
+             targets[start:end] <- colnames(mat)[a + 1:dim(mat)[2]]
+         }
+         outputPointer = outputPointer + length
+     }
+     if (includeGeneIDs) {
+         output <- matrix(nrow = length(sources), ncol = 3)
+         output[, 1] = sources
+         output[, 2] = targets
+         output[, 3] = weights
+         colnames(output) <- c("Source", "Target", "Strength")
+     }
+     else {
+         output <- matrix(nrow = (outputPointer - 1), ncol = 1)
+         output[, 1] = weights
+     }
+     output
+ }
```

0.1 Truth

Because the truth files will be in adjacency list format, we'll need a function to convert the truth function to a a matrix so they can be used by the above function. (Though we could convert directly to list format,

this method will be simple to write/verify/debug.)

```
> listToMatrix <- function(geneList, g1, g2, weights) {
+     g1 <- as.matrix(g1)
+     g2 <- as.matrix(g2)
+     weights <- as.matrix(weights)
+     mat <- matrix(rep(0, length(geneList) * length(geneList)),
+         nrow = length(geneList), ncol = length(geneList))
+     rownames(mat) <- geneList
+     colnames(mat) <- geneList
+     for (i in 1:length(g1)) {
+         a <- which(geneList == g1[i])
+         b <- which(geneList == g2[i])
+         mat[a, b] = weights[i]
+     }
+     mat
+ }
```

0.2 WGCNA

WGCNA must be downloaded and installedf first. At the time of writing, the package is available at `http://www.genetics.ucla.edu/labs/horvath/CoexpressionNetwork/Rpackages/WGCNA/`. Additionall, the following instruction will install the necessary prerequisite files:

```
install.packages(c("fields", "impute", "dynamicTreeCut", "qvalue", "flashClust", "Hmisc") )
```

We then use the following function to use WGCNA to calculate the adjacency matrix of some set of samples.

```
> wgcna <- adjacency(t(data))
```

0.3 GeneNet

Extracting a network from GeneNet is just a bit more complex.

```
> buildGeneNet <- function(data) {
+     inferred.pcor <- ggm.estimate.pcor(t(data))
+     test.results <- ggm.test.edges(inferred.pcor, direct = FALSE,
+         plot = FALSE)
+     names <- rownames(data)
+     n1 <- names[test.results$node1]
+     n2 <- names[test.results$node2]
+     res <<- data.frame(g1 = n1, g2 = n2, val = abs(test.results$pcor))
+     geneList <- rownames(data)
+     as.matrix(listToMatrix(geneList, n1, n2, abs(test.results$pcor)))
+ }
> gn <- buildGeneNet(data)
```

```
Estimating optimal shrinkage intensity lambda (correlation matrix): 0.0712

Estimate (local) false discovery rates (partial correlations):
Step 1... determine cutoff point
Step 2... estimate parameters of null distribution and eta0
Step 3... compute p-values and estimate empirical PDF/CDF
Step 4... compute q-values and local fdr
```

0.4 Space

Space will require a bit more work as there are some parameters which must be set. Also, Space strips the row names off of the data set, so we'll have to restore those.

```
> buildSpace <- function(data, method = 1) {
+     n = ncol(data)
+     p = nrow(data)
+     alpha = 1
+     l1 = 1/sqrt(n) * qnorm(1 - alpha/(2 * p^2))
+     iter = 3
+     if (method == 1) {
+         result <- space.joint(t(data), lam1 = l1 * n, lam2 = 0,
+             weight = 1, iter = iter)$ParCor
+     }
+     else if (method == 3) {
+         result <- space.joint(t(data), lam1 = l1 * n * 1.86,
+             lam2 = 0, weight = 1, iter = iter)$ParCor
+     }
+     else if (method == 4) {
+         result <- space.joint(t(data), lam1 = l1 * n * 1.61,
+             lam2 = 0, weight = 2, iter = iter)$ParCor
+     }
+     else if (method == 5) {
+         result <- space.joint(t(data), lam1 = 0, lam2 = 0, weight = 1,
+             iter = iter)$ParCor
+     }
+     colnames(result) <- rownames(data)
+     rownames(result) <- rownames(data)
+     as.matrix(result)
+ }
> space1 <- buildSpace(data, 1)
```

```
[1] "iter=1"
[1] "iter=2"
[1] "iter=3"
```

```
> save(space1, file = "space1.Rda")
> space3 <- buildSpace(data, 3)
```

```
[1] "iter=1"
[1] "iter=2"
[1] "iter=3"
```

```
> save(space3, file = "space3.Rda")
> space4 <- buildSpace(data, 4)
```

```
[1] "iter=1"
[1] "iter=2"
[1] "iter=3"
```

```
> save(space4, file = "space4.Rda")
```

We can now aggregate these matrices into a single adjacency list format which contains all of the information.

```
> getAgg <- function() {
+     agg <- cbind(matrixToList(gn, TRUE), matrixToList(space1,
+         FALSE), matrixToList(space3, FALSE), matrixToList(space4,
+         FALSE), matrixToList(wgcna, FALSE))
+     colnames(agg) <- c("Source", "Target", "GeneNet", "Space1",
+         "Space3", "Space4", "WGCNA")
+     agg
+ }
> agg <- getAgg()
> write.csv(agg, file = "ecoli.csv", row.names = FALSE)
> save(agg, file = "agg.Rda")
```

Compare Network Engines

Jeffrey D. Allen

August 11, 2011

Now we'll want to combine the output from Aracne and add them into the aggregated files from the other methods.

The outputs from aracne are available in ../../Ecoli/aracneConverted/*

So the results of Aracne are available in one directory:

```
> aracneFile <- "../../Ecoli/Ecoli expression_k0.138.adj.csv"
```

And the previously aggregated results are in another:

```
> aggFile <- "../../Results/Ecoli.allspace.csv"
```

So we can iterate through the network sizes and add in each of the columns from aracne. Unortunately, the previous results hadn't been sorted. So we'll want to sort both results before combining to ensure that the rows line up.

```
> agg <- read.csv(aggFile, header = T, row.names = NULL, stringsAsFactors = FALSE)
> agg[which(agg[, 1] > agg[, 2]), 1:2] <- agg[which(agg[, 1] >
+     agg[, 2]), 2:1]
> newAgg <- agg[order(agg$Source, agg$Target), ]
> rm(agg)
> aracne <- read.csv(aracneFile, header = TRUE, row.names = NULL)
> aracne[which(aracne[, 1] > aracne[, 2]), 1:2] <- aracne[which(aracne[,
+     1] > aracne[, 2]), 2:1]
> sortedAracne <- aracne[order(aracne$gene1, aracne$gene2), ]
> newAgg <- cbind(newAgg, sortedAracne$edge)
> colnames(newAgg)[dim(newAgg)[2]] <- "Aracne"
> write.csv(newAgg, file = "../../Results/Ecoli.aggregate.csv",
+     row.names = FALSE)
> print(paste("Done at", date()))
```

```
[1] "Done at Thu Aug 11 11:26:34 2011"
```

Compare Network Engines - NonLinear

Jeffrey D. Allen

August 10, 2011

This file will document the process by which we examine and compare various methods of constructing genetic dependency graphs.

1 Data Files

Two types of files are used and are assumed to already exist. First, is a set of files which represent the simulated expression levels for a set of genes across various samples. Second is the truth of the underlying network being simulated in these samples. For the sake of simplicity, the truth is stored in adjacency-list format.

We will first construct a list of names for the various networks. These names represent the different categories of networks we'll be testing. For instance, we simulated a "tiny" network with only a few genes, and a "large" network with hundreds of genes, etc.

```
> networkSizes = c("tiny", "small", "moderate", "middle", "large",
+     "huge")
```

For each of these networks, we simulated different experiments which varied in the number of samples they contained. This will test the ability to extract meaning from various numbers of samples from networks of various sizes.

```
> sampleCounts = c(20, 50, 100, 200, 500, 1000)
```

Next we'll convert these names and sample sizes into the names of the files:

```
> files = matrix(nrow = length(networkSizes), ncol = length(sampleCounts))
> for (i in 1:length(networkSizes)) {
+     files[i, ] = paste("../../NLSimulations/", networkSizes[i],
+         "nSamp", sampleCounts, ".csv", sep = "")
+ }
> files
```

```
     [,1]
[1,] "../../NLSimulations/tinynSamp20.csv"
[2,] "../../NLSimulations/smallnSamp20.csv"
[3,] "../../NLSimulations/moderatenSamp20.csv"
[4,] "../../NLSimulations/middlenSamp20.csv"
[5,] "../../NLSimulations/largenSamp20.csv"
[6,] "../../NLSimulations/hugenSamp20.csv"
     [,2]
[1,] "../../NLSimulations/tinynSamp50.csv"
[2,] "../../NLSimulations/smallnSamp50.csv"
[3,] "../../NLSimulations/moderatenSamp50.csv"
[4,] "../../NLSimulations/middlenSamp50.csv"
[5,] "../../NLSimulations/largenSamp50.csv"
[6,] "../../NLSimulations/hugenSamp50.csv"
     [,3]
[1,] "../../NLSimulations/tinynSamp100.csv"
[2,] "../../NLSimulations/smallnSamp100.csv"
[3,] "../../NLSimulations/moderatenSamp100.csv"
[4,] "../../NLSimulations/middlenSamp100.csv"
```

```
[5,] "../../NLSimulations/largenSamp100.csv"
[6,] "../../NLSimulations/hugenSamp100.csv"
     [,4]
[1,] "../../NLSimulations/tinynSamp200.csv"
[2,] "../../NLSimulations/smallnSamp200.csv"
[3,] "../../NLSimulations/moderatenSamp200.csv"
[4,] "../../NLSimulations/middlenSamp200.csv"
[5,] "../../NLSimulations/largenSamp200.csv"
[6,] "../../NLSimulations/hugenSamp200.csv"
     [,5]
[1,] "../../NLSimulations/tinynSamp500.csv"
[2,] "../../NLSimulations/smallnSamp500.csv"
[3,] "../../NLSimulations/moderatenSamp500.csv"
[4,] "../../NLSimulations/middlenSamp500.csv"
[5,] "../../NLSimulations/largenSamp500.csv"
[6,] "../../NLSimulations/hugenSamp500.csv"
     [,6]
[1,] "../../NLSimulations/tinynSamp1000.csv"
[2,] "../../NLSimulations/smallnSamp1000.csv"
[3,] "../../NLSimulations/moderatenSamp1000.csv"
[4,] "../../NLSimulations/middlenSamp1000.csv"
[5,] "../../NLSimulations/largenSamp1000.csv"
[6,] "../../NLSimulations/hugenSamp1000.csv"
```

At this point, all of the files of a certain network can be accessed with:

```
> tinyFiles <- files[1, ]
> tinyFiles
```

```
[1] "../../NLSimulations/tinynSamp20.csv"
[2] "../../NLSimulations/tinynSamp50.csv"
[3] "../../NLSimulations/tinynSamp100.csv"
[4] "../../NLSimulations/tinynSamp200.csv"
[5] "../../NLSimulations/tinynSamp500.csv"
[6] "../../NLSimulations/tinynSamp1000.csv"
```

and specific files can be read in with:

```
> data <- as.matrix(read.csv(tinyFiles[5], header = T, row.names = 1))
```

which presents the data file as a data.frame which gets converted to a matrix for ease of computational access later on. Note that the gene names (in this case, integral identifiers) serve as row labels while the sample identifiers are the column names. Different programs will request this data in different formats, but most should be compatible with either this or its transpose. Now that we have the samples available in a convenient format, we can begin processing the various methods.

2 Processing

2.1 Output Format

The goal of this analysis is to put all of the simulation results into aggregate files which can be used to compare the performance of each method. These files will be in adjacency-list-format and will exhaust every possible connection that isn't itself a cycle (i.e. any gene pointing to any other gene will be an option). Then the columns will each represent the output of some method.

However, all of the methods will work on matrices as input and output, so we'll need a function to convert our matrix into a list of standard format.

```
Package WGCNA version 1.11-3 loaded.
```

```
> matrixToList <- function(mat, includeGeneIDs = TRUE) {
+     arSize <- length(length(mat)/2 - dim(mat)[1]/2)
```

```
+     for (a in 1:dim(mat)[1]) {
+         for (b in 1:dim(mat)[2]) {
+             strength <- max(abs(mat[a, b]), abs(mat[b, a]))
+             mat[min(a, b), max(a, b)] <- strength
+             mat[max(a, b), min(a, b)] <- 0
+             if (a == b) {
+                 mat[a, b] = 0
+             }
+         }
+     }
+     outputPointer <- 1
+     weights <- array(arSize)
+     sources <- array(arSize)
+     targets <- array(arSize)
+     for (a in 1:(dim(mat)[1] - 1)) {
+         start <- outputPointer
+         end <- dim(mat)[2] + outputPointer - a - 1
+         length <- end - start + 1
+         weights[start:end] <- mat[a, (a + 1):dim(mat)[2]]
+         if (includeGeneIDs) {
+             sources[start:end] <- colnames(mat)[a]
+             targets[start:end] <- colnames(mat)[a + 1:dim(mat)[2]]
+         }
+         outputPointer = outputPointer + length
+     }
+     if (includeGeneIDs) {
+         output <- matrix(nrow = length(sources), ncol = 3)
+         output[, 1] = sources
+         output[, 2] = targets
+         output[, 3] = weights
+         colnames(output) <- c("Source", "Target", "Strength")
+     }
+     else {
+         output <- matrix(nrow = (outputPointer - 1), ncol = 1)
+         output[, 1] = weights
+     }
+     output
+ }
```

2.2 Truth

Because the truth files will be in adjacency list format, we'll need a function to convert the truth function to a a matrix so they can be used by the above function. (Though we could convert directly to list format, this method will be simple to write/verify/debug.)

```
> getTruthMatrix <- function(networkSizeID) {
+     truthFile <- paste("../../Simulations/truth ", networkSizes[networkSizeID],
+         ".csv", sep = "")
+     data <- read.csv(truthFile, header = T)
+     sources <- as.numeric(data$Source)
+     targets <- as.numeric(data$Target)
+     weights <- abs(as.numeric(data$regulation))
+     data <- as.matrix(read.csv(files[networkSizeID, 1], header = T,
+         row.names = 1))
+     geneList <- as.numeric(rownames(data))
+     mat <- listToMatrix(geneList, sources, targets, weights)
+     as.matrix(mat)
+ }
> listToMatrix <- function(geneList, g1, g2, weights) {
+     g1 <- as.matrix(g1)
```

```
+     g2 <- as.matrix(g2)
+     weights <- as.matrix(weights)
+     mat <- matrix(rep(0, length(geneList) * length(geneList)),
+         nrow = length(geneList), ncol = length(geneList))
+     rownames(mat) <- geneList
+     colnames(mat) <- geneList
+     for (i in 1:length(g1)) {
+         a <- which(geneList == g1[i])
+         b <- which(geneList == g2[i])
+         mat[a, b] = weights[i]
+     }
+     mat
+ }
```

2.3 WGCNA

WGCNA must be downloaded and installedf first. At the time of writing, the package is available at http://www.genetics.ucla.edu/labs/horvath/CoexpressionNetwork/Rpackages/WGCNA/. Additionall, the following instruction will install the necessary prerequisite files:

```
install.packages(c("fields", "impute", "dynamicTreeCut", "qvalue", "flashClust", "Hmisc") )
```

We then use the following function to use WGCNA to calculate the adjacency matrix of some set of samples.

```
> adj <- adjacency(t(data))
```

2.4 GeneNet

Extracting a network from GeneNet is just a bit more complex.

```
> buildGeneNet <- function(data) {
+     inferred.pcor <- ggm.estimate.pcor(t(data))
+     test.results <- ggm.test.edges(inferred.pcor, direct = FALSE,
+         plot = FALSE)
+     names <- rownames(data)
+     n1 <- as.numeric(names[test.results$node1])
+     n2 <- as.numeric(names[test.results$node2])
+     res <<- data.frame(g1 = n1, g2 = n2, val = abs(test.results$pcor))
+     geneList <- as.numeric(rownames(data))
+     as.matrix(listToMatrix(geneList, n1, n2, abs(test.results$pcor)))
+ }
```

2.5 Space

Space will require a bit more work as there are some parameters which must be set. Also, Space strips the row names off of the data set, so we'll have to restore those.

```
> buildSpace <- function(data) {
+     n = ncol(data)
+     p = nrow(data)
+     alpha = 1
+     l1 = 1/sqrt(n) * qnorm(1 - alpha/(2 * p^2))
+     iter = 3
+     result <- space.joint(t(data), lam1 = l1 * n, lam2 = 0, weight = 1,
+         iter = iter)$ParCor
+     colnames(result) <- rownames(data)
+     rownames(result) <- rownames(data)
+     as.matrix(result)
+ }
```

3 Aggregate Files

By building an aggregate file of an entire network, we can examine the performance of various algorithms or sample sizes easily. First, we'll want a way to setup an adjacency list structure which can be used as a template for the rest of the data. This structure should contain the gene identifiers for each connection as well as the true value of that connection.

```
> getTruthCols <- function(networkSizeID) {
+     truth <- getTruthMatrix(networkSizeID)
+     truth <- matrixToList(truth)
+     colnames(truth) <- c(colnames(truth)[1:2], "Truth")
+     truth
+ }
```

Next, we'll want a function to calculate the aggregates across all 4 methods for some network size and some number of samples.

```
> getAgg <- function(networkSizeID, sampleCountID) {
+     data <- as.matrix(read.csv(files[networkSizeID, sampleCountID],
+         header = T, row.names = 1))
+     agg <- cbind(matrixToList(buildGeneNet(data), FALSE), matrixToList(buildSpace(data),
+         FALSE), matrixToList(adjacency(t(data)), FALSE))
+     colnames(agg) <- c(paste("GeneNet-", sampleCounts[sampleCountID],
+         sep = ""), paste("space-", sampleCounts[sampleCountID],
+         sep = ""), paste("WGCNA-", sampleCounts[sampleCountID],
+         sep = ""))
+     agg
+ }
```

Finally, we'll want a way to iterate the above method on all sample sizes, so that we can build a file containing all of the information about one network size.

```
> getAllAggs <- function(networkSizeID) {
+     output <- getTruthCols(networkSizeID)
+     for (samples in 1:length(sampleCounts)) {
+         output <- cbind(output, getAgg(networkSizeID, samples))
+     }
+     output
+ }
```

This method can be called and the output written to a CSV file using something like:

```
> for (netSize in 1:length(networkSizes)) {
+     write.csv(getAllAggs(netSize), paste("../../Results/NLAgg",
+         networkSizes[netSize], ".csv", sep = ""), row.names = F,
+         quote = F)
+ }
```

```
Estimating optimal shrinkage intensity lambda (correlation matrix): 0.3188

Estimate (local) false discovery rates (partial correlations):
Step 1... determine cutoff point
Step 2... estimate parameters of null distribution and eta0
Step 3... compute p-values and estimate empirical PDF/CDF
Step 4... compute q-values and local fdr

[1] "iter=1"
[1] "iter=2"
[1] "iter=3"
Estimating optimal shrinkage intensity lambda (correlation matrix): 0.1527

Estimate (local) false discovery rates (partial correlations):
```

```
Step 1... determine cutoff point
Step 2... estimate parameters of null distribution and eta0
Step 3... compute p-values and estimate empirical PDF/CDF
Step 4... compute q-values and local fdr

[1] "iter=1"
[1] "iter=2"
[1] "iter=3"
Estimating optimal shrinkage intensity lambda (correlation matrix): 0.0824

Estimate (local) false discovery rates (partial correlations):
Step 1... determine cutoff point
Step 2... estimate parameters of null distribution and eta0
Step 3... compute p-values and estimate empirical PDF/CDF
Step 4... compute q-values and local fdr

[1] "iter=1"
[1] "iter=2"
[1] "iter=3"
Estimating optimal shrinkage intensity lambda (correlation matrix): 0.0442

Estimate (local) false discovery rates (partial correlations):
Step 1... determine cutoff point
Step 2... estimate parameters of null distribution and eta0
Step 3... compute p-values and estimate empirical PDF/CDF
Step 4... compute q-values and local fdr

[1] "iter=1"
[1] "iter=2"
[1] "iter=3"
Estimating optimal shrinkage intensity lambda (correlation matrix): 0.0178

Estimate (local) false discovery rates (partial correlations):
Step 1... determine cutoff point
Step 2... estimate parameters of null distribution and eta0
Step 3... compute p-values and estimate empirical PDF/CDF
Step 4... compute q-values and local fdr

[1] "iter=1"
[1] "iter=2"
[1] "iter=3"
Estimating optimal shrinkage intensity lambda (correlation matrix): 0.0088

Estimate (local) false discovery rates (partial correlations):
Step 1... determine cutoff point
Step 2... estimate parameters of null distribution and eta0
Step 3... compute p-values and estimate empirical PDF/CDF
Step 4... compute q-values and local fdr

[1] "iter=1"
[1] "iter=2"
[1] "iter=3"
Estimating optimal shrinkage intensity lambda (correlation matrix): 0.5362

Estimate (local) false discovery rates (partial correlations):
Step 1... determine cutoff point
Step 2... estimate parameters of null distribution and eta0
Step 3... compute p-values and estimate empirical PDF/CDF
Step 4... compute q-values and local fdr
```

```
[1] "iter=1"
[1] "iter=2"
[1] "iter=3"
Estimating optimal shrinkage intensity lambda (correlation matrix): 0.3159

Estimate (local) false discovery rates (partial correlations):
Step 1... determine cutoff point
Step 2... estimate parameters of null distribution and eta0
Step 3... compute p-values and estimate empirical PDF/CDF
Step 4... compute q-values and local fdr

[1] "iter=1"
[1] "iter=2"
[1] "iter=3"
Estimating optimal shrinkage intensity lambda (correlation matrix): 0.1907

Estimate (local) false discovery rates (partial correlations):
Step 1... determine cutoff point
Step 2... estimate parameters of null distribution and eta0
Step 3... compute p-values and estimate empirical PDF/CDF
Step 4... compute q-values and local fdr

[1] "iter=1"
[1] "iter=2"
[1] "iter=3"
Estimating optimal shrinkage intensity lambda (correlation matrix): 0.1045

Estimate (local) false discovery rates (partial correlations):
Step 1... determine cutoff point
Step 2... estimate parameters of null distribution and eta0
Step 3... compute p-values and estimate empirical PDF/CDF
Step 4... compute q-values and local fdr

[1] "iter=1"
[1] "iter=2"
[1] "iter=3"
Estimating optimal shrinkage intensity lambda (correlation matrix): 0.0441

Estimate (local) false discovery rates (partial correlations):
Step 1... determine cutoff point
Step 2... estimate parameters of null distribution and eta0
Step 3... compute p-values and estimate empirical PDF/CDF
Step 4... compute q-values and local fdr

[1] "iter=1"
[1] "iter=2"
[1] "iter=3"
Estimating optimal shrinkage intensity lambda (correlation matrix): 0.0223

Estimate (local) false discovery rates (partial correlations):
Step 1... determine cutoff point
Step 2... estimate parameters of null distribution and eta0
Step 3... compute p-values and estimate empirical PDF/CDF
Step 4... compute q-values and local fdr

[1] "iter=1"
[1] "iter=2"
[1] "iter=3"
```

```
Estimating optimal shrinkage intensity lambda (correlation matrix): 0.613

Estimate (local) false discovery rates (partial correlations):
Step 1... determine cutoff point
Step 2... estimate parameters of null distribution and eta0
Step 3... compute p-values and estimate empirical PDF/CDF
Step 4... compute q-values and local fdr

[1] "iter=1"
[1] "iter=2"
[1] "iter=3"
Estimating optimal shrinkage intensity lambda (correlation matrix): 0.3771

Estimate (local) false discovery rates (partial correlations):
Step 1... determine cutoff point
Step 2... estimate parameters of null distribution and eta0
Step 3... compute p-values and estimate empirical PDF/CDF
Step 4... compute q-values and local fdr

[1] "iter=1"
[1] "iter=2"
[1] "iter=3"
Estimating optimal shrinkage intensity lambda (correlation matrix): 0.2401

Estimate (local) false discovery rates (partial correlations):
Step 1... determine cutoff point
Step 2... estimate parameters of null distribution and eta0
Step 3... compute p-values and estimate empirical PDF/CDF
Step 4... compute q-values and local fdr

[1] "iter=1"
[1] "iter=2"
[1] "iter=3"
Estimating optimal shrinkage intensity lambda (correlation matrix): 0.1402

Estimate (local) false discovery rates (partial correlations):
Step 1... determine cutoff point
Step 2... estimate parameters of null distribution and eta0
Step 3... compute p-values and estimate empirical PDF/CDF
Step 4... compute q-values and local fdr

[1] "iter=1"
[1] "iter=2"
[1] "iter=3"
Estimating optimal shrinkage intensity lambda (correlation matrix): 0.0587

Estimate (local) false discovery rates (partial correlations):
Step 1... determine cutoff point
Step 2... estimate parameters of null distribution and eta0
Step 3... compute p-values and estimate empirical PDF/CDF
Step 4... compute q-values and local fdr

[1] "iter=1"
[1] "iter=2"
[1] "iter=3"
Estimating optimal shrinkage intensity lambda (correlation matrix): 0.0306

Estimate (local) false discovery rates (partial correlations):
Step 1... determine cutoff point
```

```
Step 2... estimate parameters of null distribution and eta0
Step 3... compute p-values and estimate empirical PDF/CDF
Step 4... compute q-values and local fdr

[1] "iter=1"
[1] "iter=2"
[1] "iter=3"
Estimating optimal shrinkage intensity lambda (correlation matrix): 0.7706

Estimate (local) false discovery rates (partial correlations):
Step 1... determine cutoff point
Step 2... estimate parameters of null distribution and eta0
Step 3... compute p-values and estimate empirical PDF/CDF
Step 4... compute q-values and local fdr

[1] "iter=1"
[1] "iter=2"
[1] "iter=3"
Estimating optimal shrinkage intensity lambda (correlation matrix): 0.6246

Estimate (local) false discovery rates (partial correlations):
Step 1... determine cutoff point
Step 2... estimate parameters of null distribution and eta0
Step 3... compute p-values and estimate empirical PDF/CDF
Step 4... compute q-values and local fdr

[1] "iter=1"
[1] "iter=2"
[1] "iter=3"
Estimating optimal shrinkage intensity lambda (correlation matrix): 0.4062

Estimate (local) false discovery rates (partial correlations):
Step 1... determine cutoff point
Step 2... estimate parameters of null distribution and eta0
Step 3... compute p-values and estimate empirical PDF/CDF
Step 4... compute q-values and local fdr

[1] "iter=1"
[1] "iter=2"
[1] "iter=3"
Estimating optimal shrinkage intensity lambda (correlation matrix): 0.2775

Estimate (local) false discovery rates (partial correlations):
Step 1... determine cutoff point
Step 2... estimate parameters of null distribution and eta0
Step 3... compute p-values and estimate empirical PDF/CDF
Step 4... compute q-values and local fdr

[1] "iter=1"
[1] "iter=2"
[1] "iter=3"
Estimating optimal shrinkage intensity lambda (correlation matrix): 0.1346

Estimate (local) false discovery rates (partial correlations):
Step 1... determine cutoff point
Step 2... estimate parameters of null distribution and eta0
Step 3... compute p-values and estimate empirical PDF/CDF
Step 4... compute q-values and local fdr
```

```
[1] "iter=1"
[1] "iter=2"
[1] "iter=3"
Estimating optimal shrinkage intensity lambda (correlation matrix): 0.0716

Estimate (local) false discovery rates (partial correlations):
Step 1... determine cutoff point
Step 2... estimate parameters of null distribution and eta0
Step 3... compute p-values and estimate empirical PDF/CDF
Step 4... compute q-values and local fdr

[1] "iter=1"
[1] "iter=2"
[1] "iter=3"
Estimating optimal shrinkage intensity lambda (correlation matrix): 0.839

Estimate (local) false discovery rates (partial correlations):
Step 1... determine cutoff point
Step 2... estimate parameters of null distribution and eta0
Step 3... compute p-values and estimate empirical PDF/CDF
Step 4... compute q-values and local fdr

[1] "iter=1"
[1] "iter=2"
[1] "iter=3"
Estimating optimal shrinkage intensity lambda (correlation matrix): 0.7683

Estimate (local) false discovery rates (partial correlations):
Step 1... determine cutoff point
Step 2... estimate parameters of null distribution and eta0
Step 3... compute p-values and estimate empirical PDF/CDF
Step 4... compute q-values and local fdr

[1] "iter=1"
[1] "iter=2"
[1] "iter=3"
Estimating optimal shrinkage intensity lambda (correlation matrix): 0.6361

Estimate (local) false discovery rates (partial correlations):
Step 1... determine cutoff point
Step 2... estimate parameters of null distribution and eta0
Step 3... compute p-values and estimate empirical PDF/CDF
Step 4... compute q-values and local fdr

[1] "iter=1"
[1] "iter=2"
[1] "iter=3"
Estimating optimal shrinkage intensity lambda (correlation matrix): 0.4729

Estimate (local) false discovery rates (partial correlations):
Step 1... determine cutoff point
Step 2... estimate parameters of null distribution and eta0
Step 3... compute p-values and estimate empirical PDF/CDF
Step 4... compute q-values and local fdr

[1] "iter=1"
[1] "iter=2"
[1] "iter=3"
Estimating optimal shrinkage intensity lambda (correlation matrix): 0.2655
```

```
Estimate (local) false discovery rates (partial correlations):
Step 1... determine cutoff point
Step 2... estimate parameters of null distribution and eta0
Step 3... compute p-values and estimate empirical PDF/CDF
Step 4... compute q-values and local fdr

[1] "iter=1"
[1] "iter=2"
[1] "iter=3"
Estimating optimal shrinkage intensity lambda (correlation matrix): 0.1527

Estimate (local) false discovery rates (partial correlations):
Step 1... determine cutoff point
Step 2... estimate parameters of null distribution and eta0
Step 3... compute p-values and estimate empirical PDF/CDF
Step 4... compute q-values and local fdr

[1] "iter=1"
[1] "iter=2"
[1] "iter=3"
Estimating optimal shrinkage intensity lambda (correlation matrix): 0.9124

Estimate (local) false discovery rates (partial correlations):
Step 1... determine cutoff point
Step 2... estimate parameters of null distribution and eta0
Step 3... compute p-values and estimate empirical PDF/CDF
Step 4... compute q-values and local fdr

[1] "iter=1"
[1] "iter=2"
[1] "iter=3"
Estimating optimal shrinkage intensity lambda (correlation matrix): 0.8698

Estimate (local) false discovery rates (partial correlations):
Step 1... determine cutoff point
Step 2... estimate parameters of null distribution and eta0
Step 3... compute p-values and estimate empirical PDF/CDF
Step 4... compute q-values and local fdr

[1] "iter=1"
[1] "iter=2"
[1] "iter=3"
Estimating optimal shrinkage intensity lambda (correlation matrix): 0.7965

Estimate (local) false discovery rates (partial correlations):
Step 1... determine cutoff point
Step 2... estimate parameters of null distribution and eta0
Step 3... compute p-values and estimate empirical PDF/CDF
Step 4... compute q-values and local fdr

[1] "iter=1"
[1] "iter=2"
[1] "iter=3"
Estimating optimal shrinkage intensity lambda (correlation matrix): 0.6623

Estimate (local) false discovery rates (partial correlations):
Step 1... determine cutoff point
Step 2... estimate parameters of null distribution and eta0
```

```
Step 3... compute p-values and estimate empirical PDF/CDF
Step 4... compute q-values and local fdr

[1] "iter=1"
[1] "iter=2"
[1] "iter=3"
Estimating optimal shrinkage intensity lambda (correlation matrix): 0.4416

Estimate (local) false discovery rates (partial correlations):
Step 1... determine cutoff point
Step 2... estimate parameters of null distribution and eta0
Step 3... compute p-values and estimate empirical PDF/CDF
Step 4... compute q-values and local fdr

[1] "iter=1"
[1] "iter=2"
[1] "iter=3"
Estimating optimal shrinkage intensity lambda (correlation matrix): 0.2842

Estimate (local) false discovery rates (partial correlations):
Step 1... determine cutoff point
Step 2... estimate parameters of null distribution and eta0
Step 3... compute p-values and estimate empirical PDF/CDF
Step 4... compute q-values and local fdr

[1] "iter=1"
[1] "iter=2"
[1] "iter=3"
```

Compare Network Engines

Jeffrey D. Allen

August 10, 2011

Now we'll want to combine the output from Aracne and add them into the aggregated files from the other methods.

The outputs from aracne are available in ../../Results/aracneConverted/*

We will first construct a list of names for the various networks. These names represent the different categories of networks we'll be testing. For instance, we simulated a "tiny" network with only a few genes, and a "large" network with hundreds of genes, etc.

```
> networkSizes = c("tiny", "small", "moderate", "middle", "large",
+     "huge")
```

For each of these networks, we simulated different experiments which varied in the number of samples they contained. This will test the ability to extract meaning from various numbers of samples from networks of various sizes.

```
> sampleCounts = c(20, 50, 100, 200, 500, 1000)
```

Next we'll convert these names and sample sizes into the names of the files:

```
> files = matrix(nrow = length(networkSizes), ncol = length(sampleCounts))
> for (i in 1:length(networkSizes)) {
+     files[i, ] = paste(networkSizes[i], "nSamp", sampleCounts,
+         ".csv", sep = "")
+ }
> files
```

```
     [,1]                   [,2]                   [,3]
[1,] "tinynSamp20.csv"      "tinynSamp50.csv"      "tinynSamp100.csv"
[2,] "smallnSamp20.csv"     "smallnSamp50.csv"     "smallnSamp100.csv"
[3,] "moderatenSamp20.csv"  "moderatenSamp50.csv"  "moderatenSamp100.csv"
[4,] "middlenSamp20.csv"    "middlenSamp50.csv"    "middlenSamp100.csv"
[5,] "largenSamp20.csv"     "largenSamp50.csv"     "largenSamp100.csv"
[6,] "hugenSamp20.csv"      "hugenSamp50.csv"      "hugenSamp100.csv"
     [,4]                    [,5]                    [,6]
[1,] "tinynSamp200.csv"      "tinynSamp500.csv"      "tinynSamp1000.csv"
[2,] "smallnSamp200.csv"     "smallnSamp500.csv"     "smallnSamp1000.csv"
[3,] "moderatenSamp200.csv"  "moderatenSamp500.csv"  "moderatenSamp1000.csv"
[4,] "middlenSamp200.csv"    "middlenSamp500.csv"    "middlenSamp1000.csv"
[5,] "largenSamp200.csv"     "largenSamp500.csv"     "largenSamp1000.csv"
[6,] "hugenSamp200.csv"      "hugenSamp500.csv"      "hugenSamp1000.csv"
```

So the results of Aracne are available in one directory:

```
> aracneFiles <- matrix(paste("../../Results/NLaracneConverted/",
+     files, ".aracne.out.csv", sep = ""), nrow = 6)
```

And the previously aggregated results are in another:

```
> aggFiles <- paste("../../Results/NLAgg", networkSizes, ".csv",
+     sep = "")
```

So we can iterate through the network sizes and add in each of the columns from aracne. Unortunately, the previous results hadn't been sorted. So we'll want to sort both results before combining to ensure that the rows line up.

```
> for (siz in 1:length(networkSizes)) {
+     agg <- read.csv(aggFiles[siz], header = T, row.names = NULL)
+     agg[which(agg[, 1] > agg[, 2]), 1:2] <- agg[which(agg[, 1] >
+         agg[, 2]), 2:1]
+     aggSorted <- agg[order(agg$Source, agg$Target), ]
+     rm(agg)
+     newAgg <- aggSorted[, 1:3]
+     numMethods <- 3
+     oldAggIndex <- 4
+     newAggIndex <- 4
+     sampleIndex <- 1
+     for (sample in 1:length(sampleCounts)) {
+         newAgg[, newAggIndex:(newAggIndex + numMethods - 1)] <- aggSorted[,
+             oldAggIndex:(oldAggIndex + numMethods - 1)]
+         aracne <- read.csv(aracneFiles[siz, sample], header = TRUE,
+             row.names = NULL)
+         aracne[which(aracne[, 1] > aracne[, 2]), 1:2] <- aracne[which(aracne[,
+             1] > aracne[, 2]), 2:1]
+         sortedAracne <- aracne[order(aracne$gene1, aracne$gene2),
+             ]
+         newAgg[, newAggIndex + numMethods] <- sortedAracne$edge
+         colnames(newAgg)[newAggIndex + numMethods] <- paste("Aracne.",
+             sampleCounts[sample], sep = "")
+         sampleIndex <- sampleIndex
+         newAggIndex <- newAggIndex + numMethods + 1
+         oldAggIndex <- oldAggIndex + numMethods
+     }
+     print(paste("Writing CSV for", networkSizes[siz], "network..."))
+     write.csv(newAgg, file = paste("../../Results/NLaracneAggregate.",
+         networkSizes[siz], ".csv", sep = ""), row.names = FALSE)
+     print(paste("Done at", date()))
+ }

[1] "Writing CSV for tiny network..."
[1] "Done at Wed Aug 10 12:18:18 2011"
[1] "Writing CSV for small network..."
[1] "Done at Wed Aug 10 12:18:18 2011"
[1] "Writing CSV for moderate network..."
[1] "Done at Wed Aug 10 12:18:18 2011"
[1] "Writing CSV for middle network..."
[1] "Done at Wed Aug 10 12:18:22 2011"
[1] "Writing CSV for large network..."
[1] "Done at Wed Aug 10 12:18:52 2011"
[1] "Writing CSV for huge network..."
[1] "Done at Wed Aug 10 12:22:02 2011"
```

AUC and pAUC Calculations

Jeffrey D. Allen

August 10, 2011

This paper will document the process of generating p/AUC graphs based on the aggreagate files already created. These will graphically and quantitatively represent the performance of various algorithms of constructing gene networks.

1 Input Files

We will first construct a list of names for the various networks. These names represent the different categories of networks we'll be testing. For instance, we simulated a "tiny" network with only a few genes, and a "large" network with hundreds of genes, etc. We have files named in one nomenclature but want to use a different naming convention for the output formats.

```
> networkSizes = c("tiny", "small", "moderate", "middle", "large",
+     "huge")
> outputSizes = c("17 Gene Network", "44 Gene Network", "83 Gene Network",
+     "231 Gene Network", "612 Gene Network", "1344 Gene Network")
```

For each of these networks, we simulated different experiments which varied in the number of samples they contained. This will test the ability to extract meaning from various numbers of samples from networks of various sizes.

```
> sampleCounts = c(20, 50, 100, 200, 500, 1000)
```

We'll then need to be able to read in the aggregate files:

```
> getAgg <- function(sizeID) {
+     fileName <- paste("../../Results/NLaracneAggregate.", networkSizes[sizeID],
+         ".csv", sep = "")
+     fileName
+     agg <- read.csv(fileName)
+     agg[, 3][which(agg[, 3] > 0)] = 1
+     agg
+ }
```

We can now begin to do some analysis on each table.

2 ROCs

We'll first graphically represent the ROC curves for each method and/or network size.

```
> library(ROCR)
> getROC <- function(truth, predicted) {
+     pred <- prediction(predicted, truth)
+     perf <- performance(pred, "sens", "spec")
+     perf
+ }
> calculateNetworkROCs <- function(networkSize) {
+     agg <- getAgg(networkSize)
+     truth <- agg[, 3]
+     par(mfrow = c(6, 5), mar = c(4, 4, 3, 1))
```

```
+     for (i in 4:length(agg[1, ])) {
+         pred <- agg[, i]
+         if (!is.na(sum(pred))) {
+             roc <- getROC(truth, pred)
+             plot(roc, main = colnames(agg)[i])
+         }
+         else {
+             plot(0, 0, main = colnames(agg[i]))
+         }
+     }
+ }
> calculateNetworkROCs(1)
```

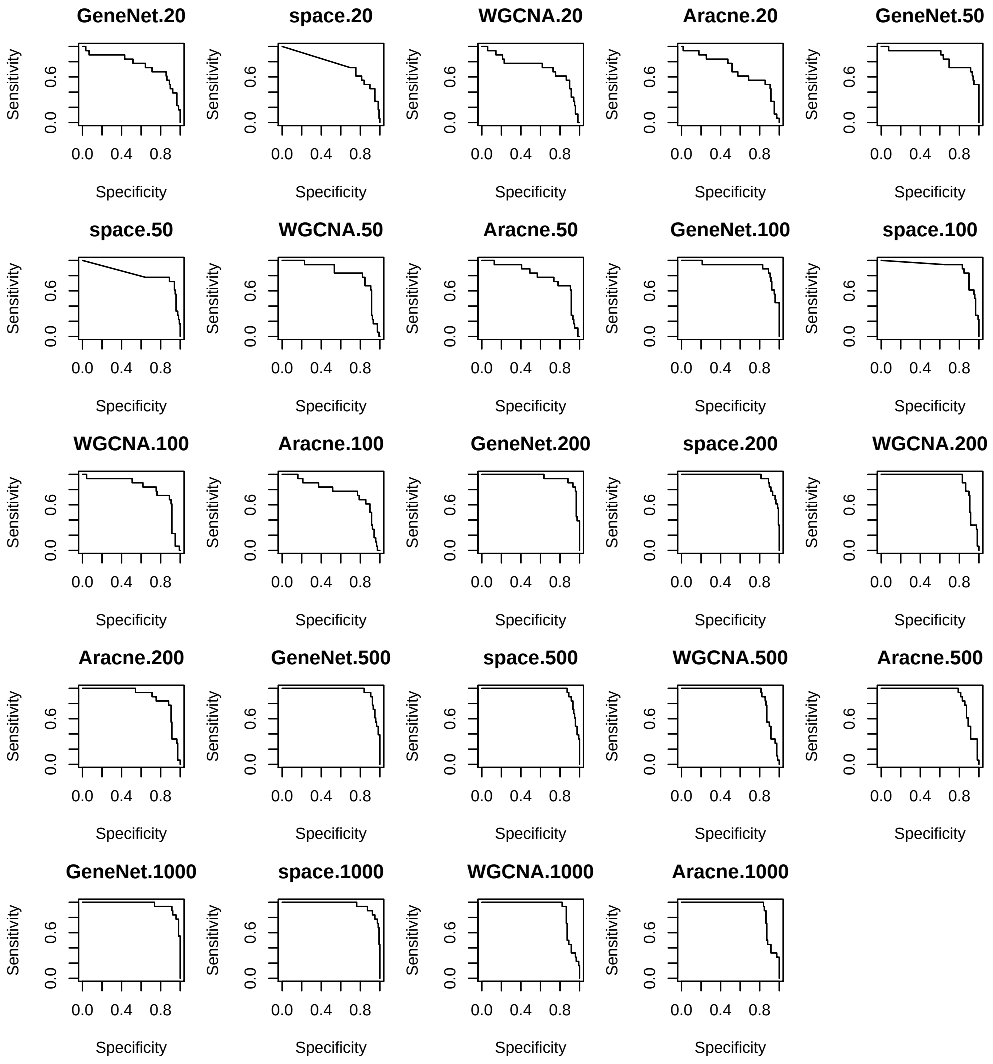

3 AUCs

We'll first want to calculate a matrix of AUCs to quantify the performance of each method. We can compute a matrix which is setup to allow for easy analysis of either method-based or network-based performance.

```
> library(ROCR)
> getAUC <- function(truth, predicted) {
+     pred <- prediction(predicted, truth)
+     auc <- performance(pred, "auc")@y.values[[1]]
+     auc
+ }
> AUCsToMatrix <- function(AUC) {
+     nSamples <- length(sampleCounts)
+     nMethods <- length(AUC)/nSamples
+     if (as.integer(nMethods) != nMethods) {
+         return
+     }
+     AUCs <- matrix(nrow = nSamples, ncol = nMethods)
+     for (i in 1:length(AUC)) {
+         method <- i%%nMethods
+         if (method == 0) {
+             method = nMethods
+         }
+         AUCs[(i - 1)%/%nMethods + 1, method] <- AUC[i]
+     }
+     methodNames <- array()
+     for (i in 1:length(AUCs[1, ])) {
+         methodNames[i] <- strsplit(names(AUC)[i], ".", fixed = TRUE)[[1]][1]
+     }
+     colnames(AUCs) <- methodNames
+     rownames(AUCs) = sampleCounts
+     AUCs
+ }
> calculateNetworkAUCs <- function(networkSize) {
+     agg <- getAgg(networkSize)
+     truth <- agg[, 3]
+     AUCs <- array()
+     for (i in 4:length(agg[1, ])) {
+         pred <- agg[, i]
+         if (!is.na(sum(pred))) {
+             auc <- getAUC(truth, pred)
+         }
+         else {
+             auc <- NA
+         }
+         AUCs[i] <- auc
+     }
+     AUCs <- AUCs[4:length(AUCs)]
+     names(AUCs) <- colnames(agg[4:length(agg[1, ])])
+     AUCsToMatrix(AUCs)
+ }
> calculateNetworkAUCs(1)
```

```
       GeneNet     space     WGCNA    Aracne
20   0.7622411 0.7551789 0.7226930 0.6972693
50   0.8582863 0.8215631 0.8338041 0.7942561
100  0.9147834 0.9088983 0.8182674 0.7735405
200  0.9543315 0.9623352 0.9204331 0.8912429
500  0.9618644 0.9604520 0.9086629 0.9081921
1000 0.9703390 0.9684557 0.9114878 0.9143126
```

This matrix can then be plotted in a variety of ways to emphasize performance differences from one method to the next. Or, to plot them everything on one graph per network size:

```
> plotAUC <- function(AUCs, netsize, pAUC = FALSE, ylimitsOrig = NULL) {
+     if (is.null(ylimitsOrig)) {
```

```
+         minY = min(AUCs[which(!is.na(AUCs))])
+         maxY = max(AUCs[which(!is.na(AUCs))])
+         ylimits = c(minY, maxY)
+     }
+     else {
+         ylimits <- ylimitsOrig
+     }
+     if (pAUC) {
+         yLab = "pAUC"
+     }
+     else {
+         yLab = "AUC"
+     }
+     plot(sampleCounts, AUCs[, 1], main = outputSizes[netsize],
+         type = "b", col = 1, pch = 1, ylim = ylimits, xlab = "Sample Size",
+         ylab = yLab)
+     for (method in 2:length(AUCs[1, ])) {
+         lines(sampleCounts, AUCs[, method], col = method, pch = method,
+             type = "b", lty = method)
+     }
+     isAllNA <- function(x) {
+         all(is.na(x))
+     }
+     legend(700, mean(ylimits), colnames(AUCs)[!apply(AUCs, 2,
+         isAllNA)], col = which(!apply(AUCs, 2, isAllNA)), lty = which(!apply(AUCs,
+         2, isAllNA)), bty = "n", pch = which(!apply(AUCs, 2,
+         isAllNA)))
+ }
> plotSingle <- function(netsize, pAUC = FALSE, ylimitsOrig = NULL) {
+     if (pAUC) {
+         AUCs <- calculateNetworkpAUCs(netsize)
+     }
+     else {
+         AUCs <- calculateNetworkAUCs(netsize)
+     }
+     plotAUC(AUCs, netsize, pAUC, ylimitsOrig)
+ }
> plotAUCs <- function(pAUC = FALSE, ylimits = NULL) {
+     ylimitsOrig <- ylimits
+     par(mfrow = c(3, 2), mar = c(4, 4, 3, 1))
+     for (netsize in 1:length(networkSizes)) {
+         plotSingle(netsize, pAUC, ylimits)
+     }
+ }
> plotAUCs(FALSE)
```

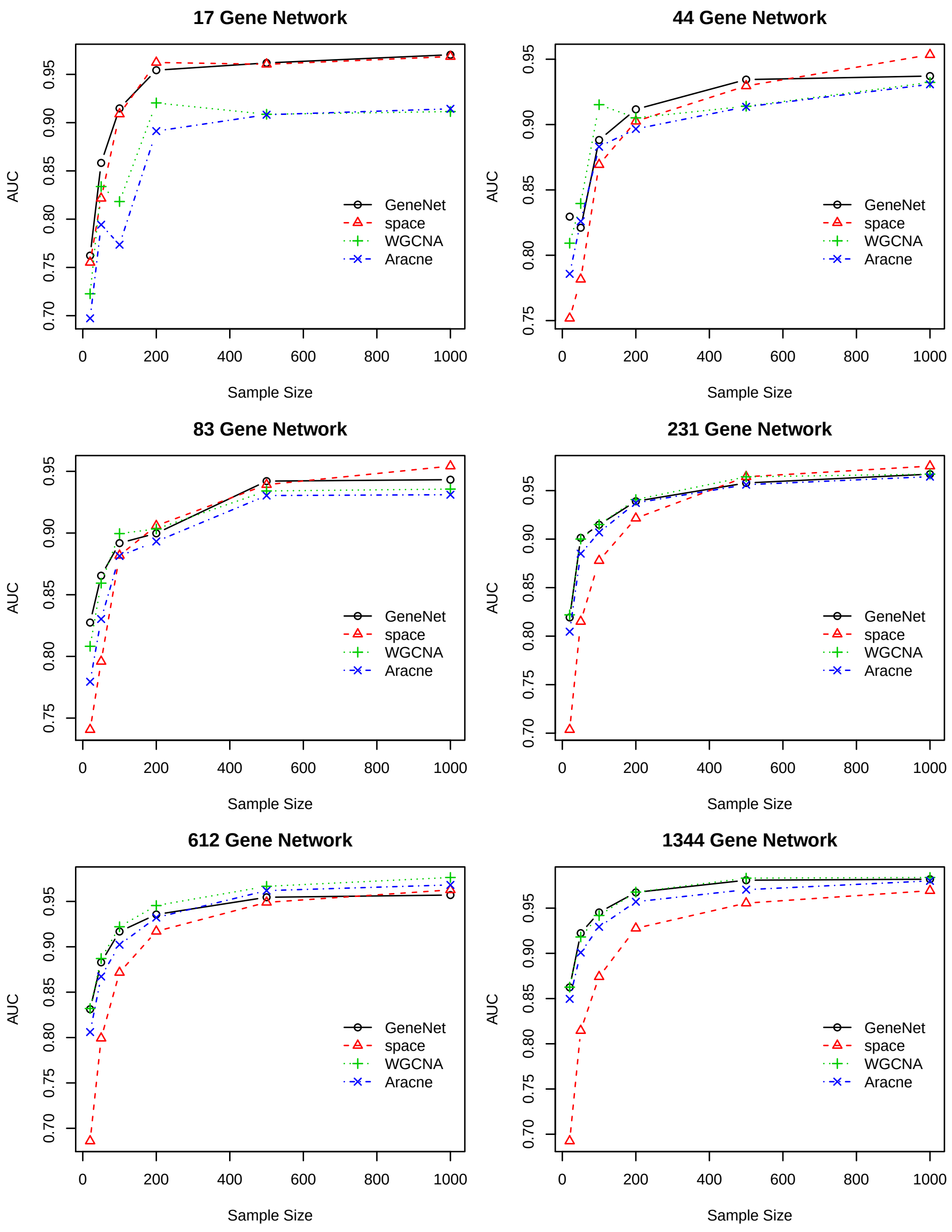

4 pAUCs

Finally, we'll want to analyze the pAUCs for each method.

```
> getpAUC <- function(truth, predicted, threshold = 0.005) {
+     pred <- prediction(predicted, truth)
+     auc <- performance(pred, "auc", fpr.stop = threshold)@y.values[[1]]
+     auc
+ }
> calculateNetworkpAUCs <- function(networkSize) {
+     agg <- getAgg(networkSize)
+     truth <- agg[, 3]
+     pAUCs <- array()
+     for (i in 4:length(agg[1, ])) {
+         pred <- agg[, i]
+         if (!is.na(sum(pred))) {
+             pauc <- getpAUC(truth, pred)
```

```
+           }
+           else {
+               pauc <- NA
+           }
+           pAUCs[i] <- pauc
+       }
+       pAUCs <- pAUCs[4:length(pAUCs)]
+       names(pAUCs) <- colnames(agg[4:length(agg[1, ])])
+       AUCsToMatrix(pAUCs)
+ }
> plotAUCs(TRUE, ylim = NULL)
```

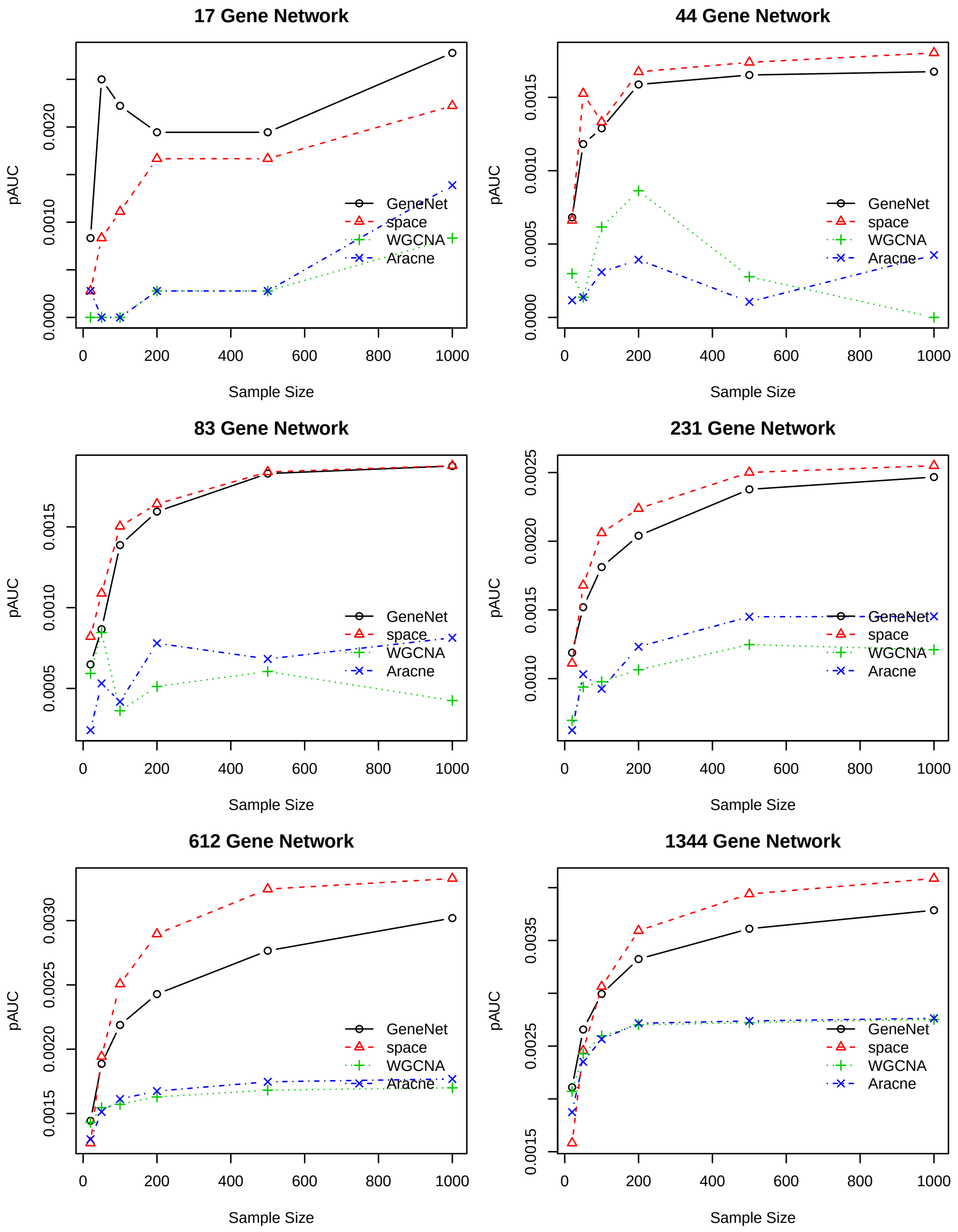

5 P vs TP

We now can plot out the number of positives vs. the number of true positives.

```
> pvstp <- function(networkSize, threshold = 0) {
+       agg <- getAgg(networkSize)
```

```
+     truth <- agg[, 3]
+     p <- apply(agg[, 4:length(agg[1, ])] > threshold, 2, sum)
+     tp <- array()
+     for (i in 4:length(agg[1, ])) {
+         tp[i - 3] <- sum((agg[, 3] != 0) & (agg[, 3] == (agg[,
+             i] > threshold)))
+     }
+     names(tp) <- names(p)
+     return(list(p = p, tp = tp))
+ }
```

But it turns out that's not what we want to do. What we want to do is plat the threshold-independent performance of each method for the Very Large network @ a sample size of 200. To do that, we'll write a simpler function that iterates through the thresholds. We could reduce the data and deduce only those coordinates at which the value would change (i.e. the only values which need to be plotted). Or we could just estimate that by plotting at even intervals over 100 points on the x-axis. We'll do that.

This may be something ROCR could do.

```
> calcPandTP <- function(truth, predicted, threshold) {
+     p <- sum(as.integer(predicted >= threshold))
+     pos <- which(predicted >= threshold)
+     tp <- sum(as.integer(truth[pos] > 0))
+     return(list(p = p, tp = tp))
+ }
> calcLine <- function(truth, predicted, steps = 1000) {
+     if (length(predicted) > steps) {
+         breaks <- array(dim = steps)
+         reductionFactor <- length(predicted)/steps
+         for (i in 0:steps) {
+             breaks[i] = predicted[as.integer(i * reductionFactor)]
+         }
+     }
+     else {
+         breaks <- predicted
+     }
+     xs <- array(dim = length(predicted) + 1)
+     ys <- array(dim = length(predicted) + 1)
+     sortedP <- sort(breaks)
+     for (i in 0:length(sortedP)) {
+         if (i == 0) {
+             thresh = 0
+         }
+         else {
+             thresh <- sortedP[i]
+         }
+         res <- calcPandTP(truth, predicted, thresh)
+         xs[i + 1] <- res$p
+         ys[i + 1] <- res$tp
+     }
+     list(x = xs, y = ys)
+ }
> plotPvsTP <- function(networkSize, sampleCountID, xCutoff = NA,
+     invert = FALSE) {
+     agg <- getAgg(networkSize)
+     nSamples <- length(sampleCounts)
+     nMethods <- (dim(agg)[2] - 3)/nSamples
+     methodTitles <- array(dim = nMethods)
+     plottedMethods <- array(dim = nMethods)
+     isAllNA <- function(x) {
+         all(is.na(x))
```

```
+     }
+     legendY <- 0
+     for (method in 1:nMethods) {
+         methodTitles[method] <- strsplit(colnames(agg)[3 + (nMethods *
+             (sampleCountID - 1)) + method], ".", fixed = TRUE)[[1]][1]
+         if (isAllNA(agg[, 3 + (nMethods * (sampleCountID - 1)) +
+             method])) {
+             plottedMethods[method] <- FALSE
+         }
+         else {
+             plottedMethods[method] <- TRUE
+         }
+         line <- calcLine(truth = agg[, 3], predicted = agg[,
+             3 + (nMethods * (sampleCountID - 1)) + method])
+         if (method == 1) {
+             legendX = 0
+             if (is.na(xCutoff)) {
+                 xLimits = NULL
+                 legendX = max(line$x)/2
+             }
+             else {
+                 xLimits = c(0, xCutoff)
+                 legendX = xCutoff/2
+             }
+             xLabel <- "# Total Positives"
+             yLabel <- "# True Positives"
+             if (invert) {
+                 buffer <- line$x
+                 line$x <- line$y
+                 line$y <- buffer
+             }
+             if (!invert) {
+                 plot(line$x, line$y, col = method, pch = method,
+                   type = "l", ylab = yLabel, xlab = xLabel, main = paste("Positives vs. True Positives
+                     outputSizes[networkSize], "with", sampleCounts[sampleCountID],
+                     "samples"), xlim = xLimits, lwd = 2)
+             }
+             else {
+                 plot(line$x, line$y, col = method, pch = method,
+                   type = "l", ylab = xLabel, xlab = yLabel, main = paste("Positives vs. True Positives
+                     outputSizes[networkSize], "with", sampleCounts[sampleCountID],
+                     "samples"), ylim = xLimits, lwd = 2)
+             }
+         }
+         else {
+             if (!invert) {
+                 lines(line$x, line$y, col = method, pch = method,
+                   lty = method, lwd = 2)
+             }
+             else {
+                 lines(line$y, line$x, col = method, pch = method,
+                   lty = method, lw2 = 2)
+             }
+         }
+         legendY <- max(legendY, max(line$y[which(!is.na(line$y))])/3)
+     }
+     if (!invert) {
+         legend(legendX, legendY, methodTitles[plottedMethods],
+             col = which(plottedMethods), pch = "", lty = which(plottedMethods),
```

```
+               bty = "n")
+       }
+       else {
+           legend(0, legendX * 1.5, methodTitles[plottedMethods],
+               col = which(plottedMethods), pch = "", lty = which(plottedMethods),
+               bty = "n")
+       }
+ }
```

In the paper we'll include the data for the 1344 gene network with 200 samples. This network is networkID = 6 and sampleSize of 200 is #4.

We essentially are looking at the "pAUC" equivalent of this function, however. We really just want to see the "interesting"part of the curve near 0. We'll plot out a few different versions of this graph.

Thus, we'll build the image with the following function:

```
> par(mfrow = c(1, 1))
> plotPvsTP(networkSize = 6, sampleCountID = 4, 2000)
```

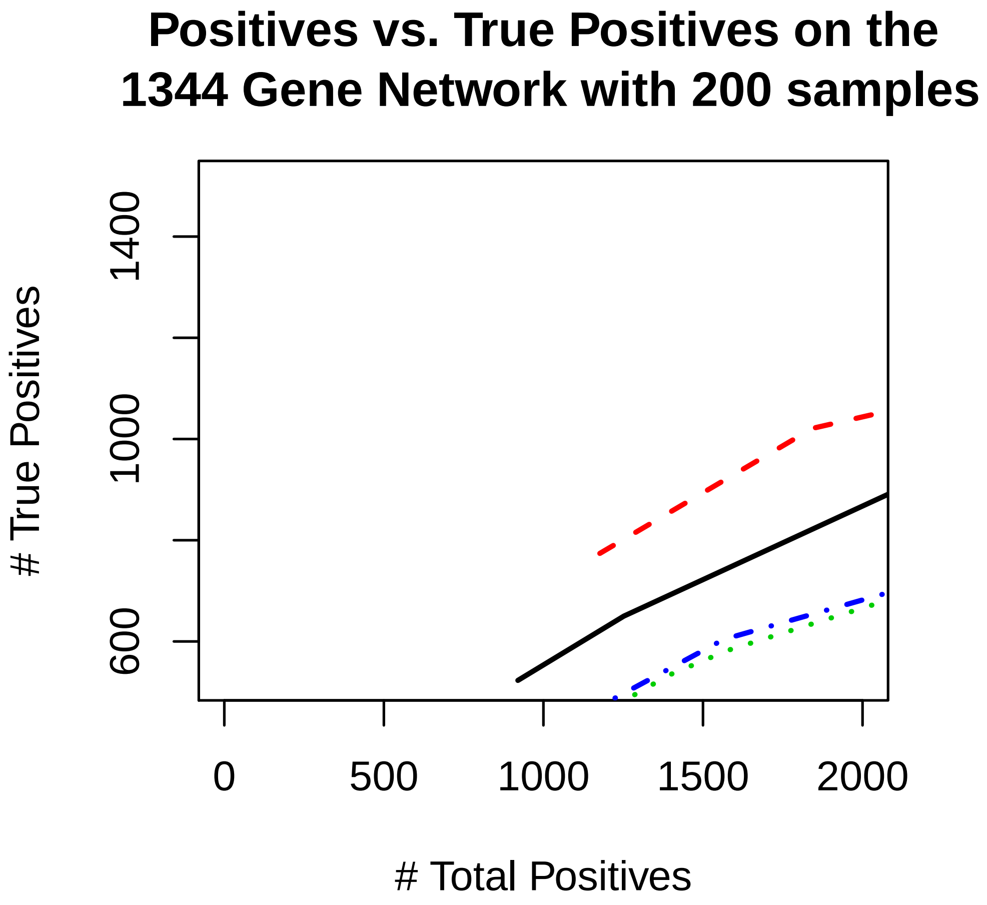


```
> par(mfrow = c(1, 1))
> plotPvsTP(networkSize = 6, sampleCountID = 4, 4000)
```

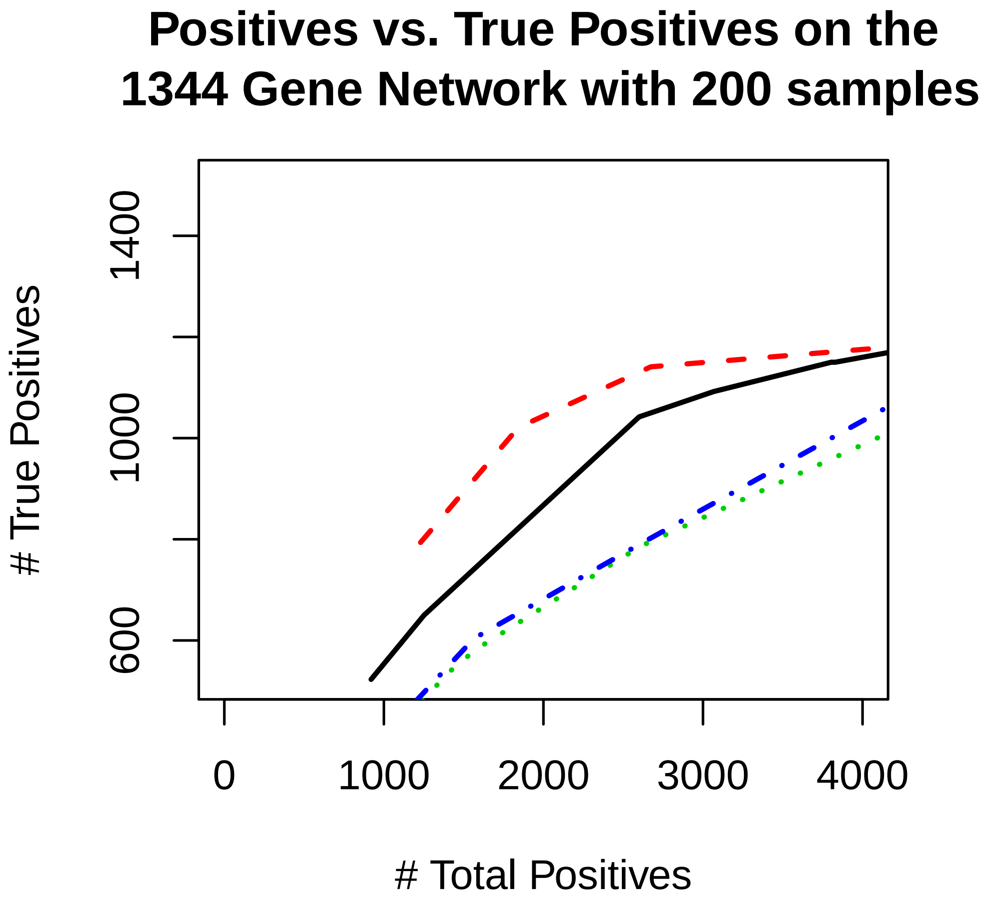


```
> par(mfrow = c(1, 1))
> plotPvsTP(networkSize = 6, sampleCountID = 4, 5000)
```

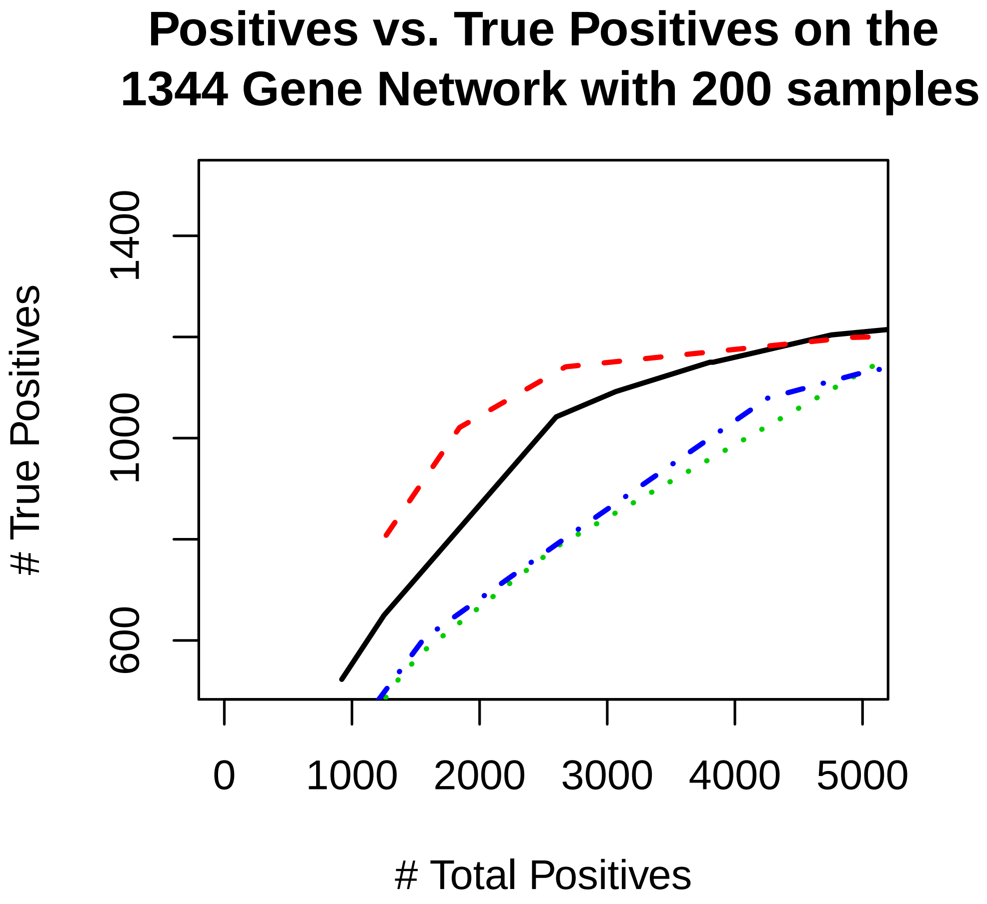

Finally, we'll want to also include the inverted pictures, in case we'd rather display them that way.

```
> par(mfrow = c(1, 1))
> plotPvsTP(networkSize = 6, sampleCountID = 4, 2000, TRUE)
```

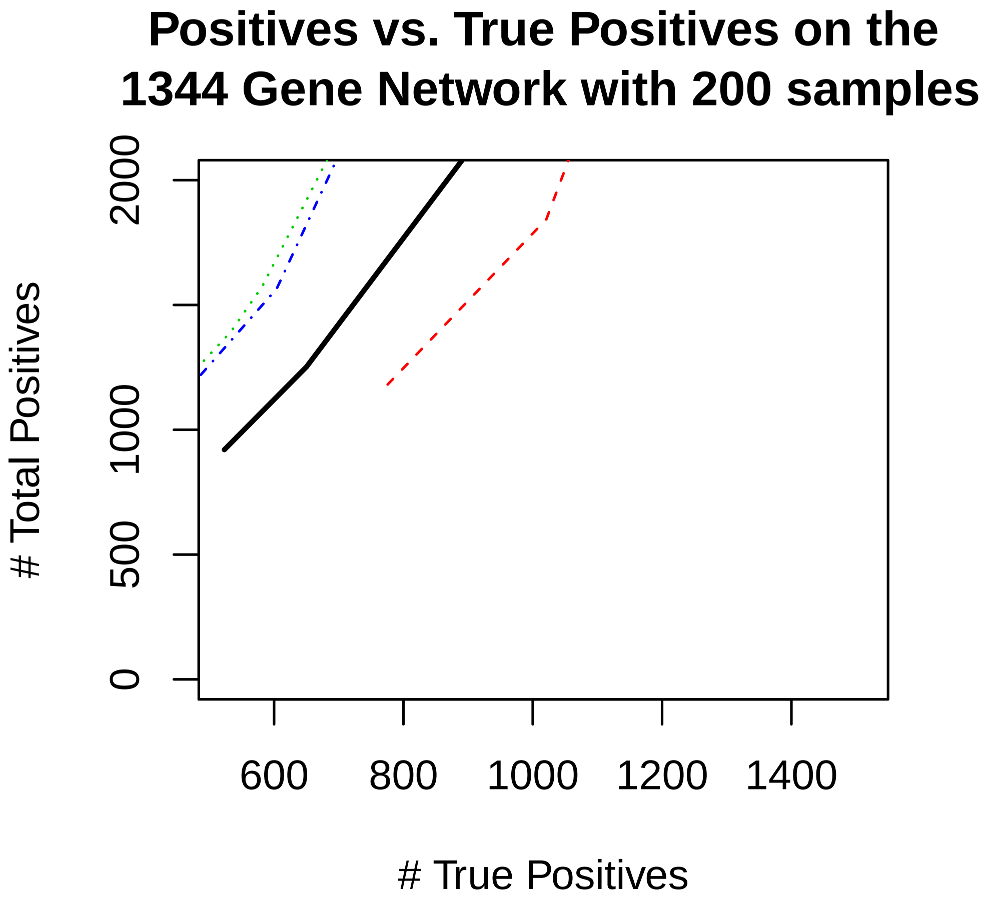


```
> par(mfrow = c(1, 1))
> plotPvsTP(networkSize = 6, sampleCountID = 4, 4000, TRUE)
```

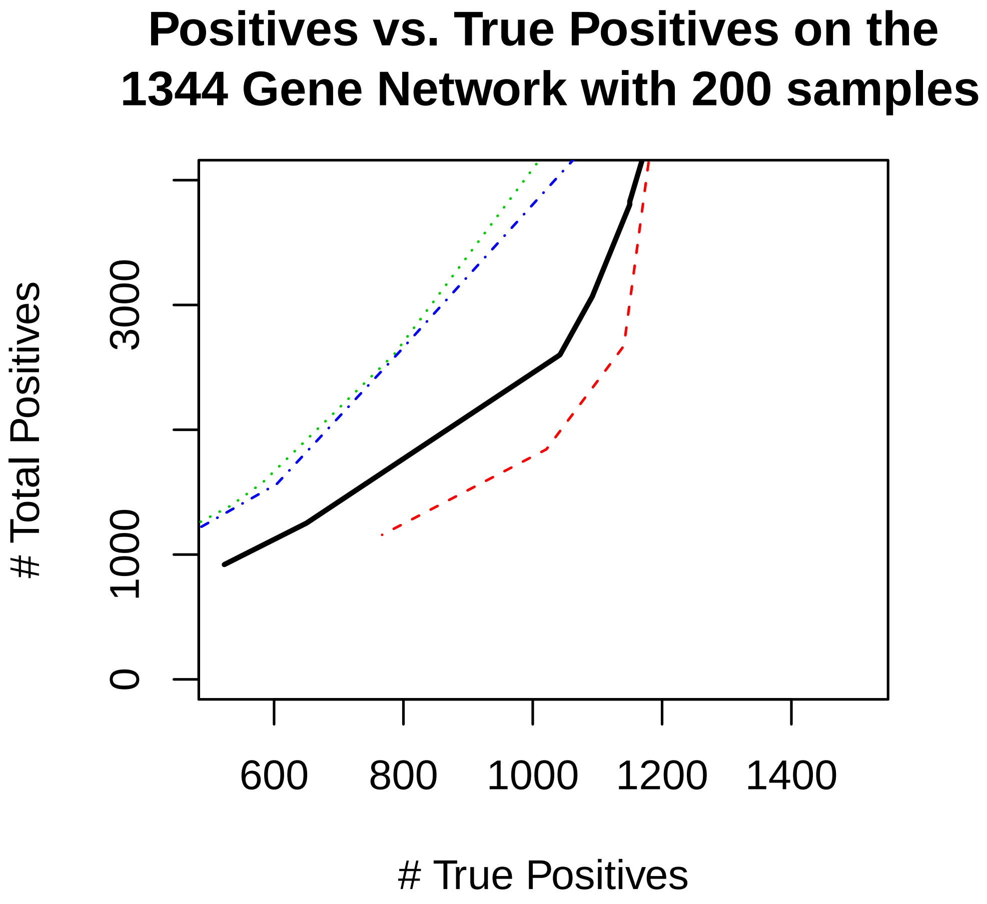


```
> par(mfrow = c(1, 1))
> plotPvsTP(networkSize = 6, sampleCountID = 4, 5000, TRUE)
```

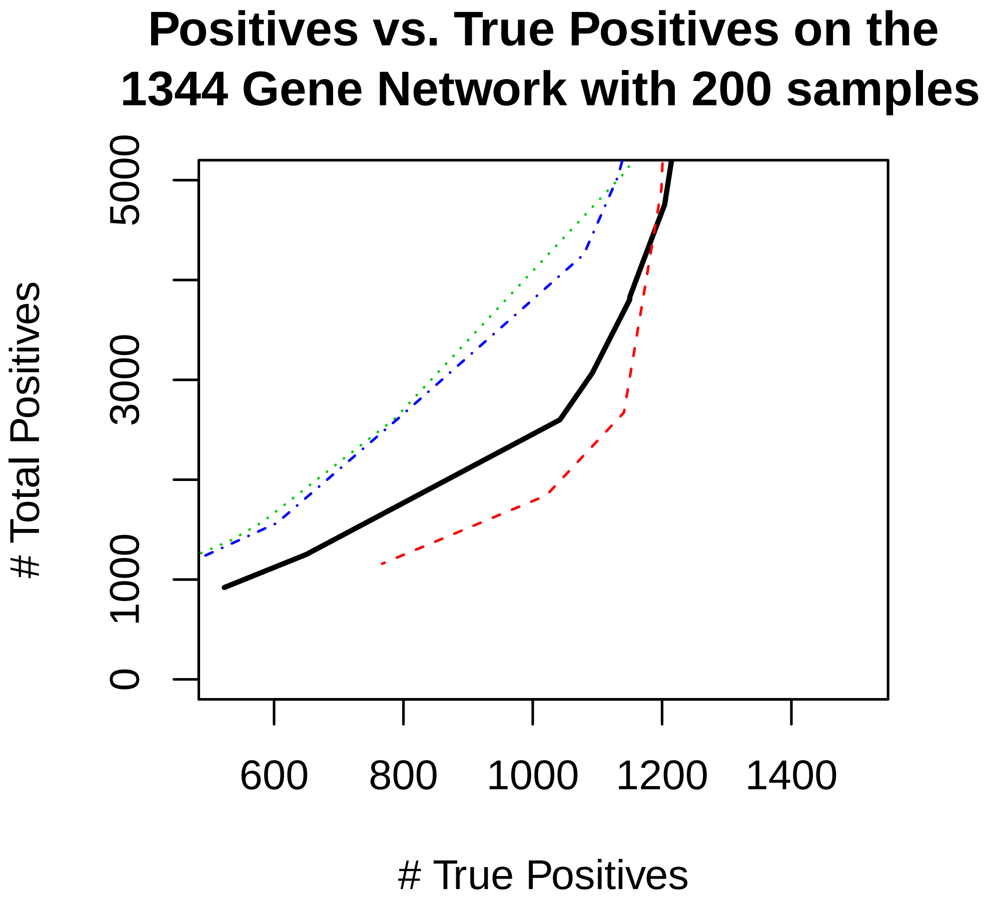

6 Precision-Recall

We'll now generate precision-recall graphs for the network closest to the biological network we'll be testing. This is the 612 gene network with 500 samples.

```
> calcPRLine <- function(truth, predicted, steps = 1000) {
+     if (length(predicted) > steps) {
+         breaks <- array(dim = steps)
+         reductionFactor <- length(predicted)/steps
+         for (i in 0:steps) {
+             breaks[i] = predicted[as.integer(i * reductionFactor)]
+         }
+     }
+     else {
+         breaks <- predicted
+     }
+     xs <- array(dim = length(predicted) + 1)
+     ys <- array(dim = length(predicted) + 1)
+     sortedP <- sort(breaks)
+     for (i in 0:length(sortedP)) {
+         if (i == 0) {
+             thresh = 0
+         }
+         else {
+             thresh <- sortedP[i]
+         }
```

```
+         res <- calcPandTP(truth, predicted, thresh)
+         xs[i + 1] <- res$tp/sum(truth > 0)
+         ys[i + 1] <- res$tp/res$p
+     }
+     list(x = xs, y = ys)
+ }
> plotPRSamples <- function(networkSize, method) {
+     agg <- getAgg(networkSize)
+     nSamples <- length(sampleCounts)
+     nMethods <- (dim(agg)[2] - 3)/nSamples
+     isAllNA <- function(x) {
+         all(is.na(x))
+     }
+     for (sampleCountID in 1:length(sampleCounts)) {
+         methodTitle <- strsplit(colnames(agg)[3 + (nMethods *
+             (sampleCountID - 1)) + method], ".", fixed = TRUE)[[1]][1]
+         line <- calcPRLine(truth = agg[, 3], predicted = agg[,
+             3 + (nMethods * (sampleCountID - 1)) + method])
+         if (sampleCountID == 1) {
+             xLimits = NULL
+             xLabel <- "Recall"
+             yLabel <- "Precision"
+             plot(line$x, line$y, col = sampleCountID, pch = sampleCountID,
+                 type = "l", ylab = yLabel, xlab = xLabel, main = paste("Precision-Recall Curve of",
+                   methodTitle, "\non the", outputSizes[networkSize]),
+                 xlim = c(0, 1), ylim = c(0, 1), lwd = 2)
+         }
+         else {
+             lines(line$x, line$y, col = sampleCountID, pch = sampleCountID,
+                 lty = sampleCountID, lwd = 2)
+         }
+     }
+     legend(0, 0.3, paste(sampleCounts, "Samples"), col = 1:length(sampleCounts),
+         pch = "", lty = 1:length(sampleCounts), bty = "n")
+ }
> par(mfrow = c(2, 2))
> for (i in 1:4) {
+     plotPRSamples(1, i)
+ }
```

Or if we want to do a PR curve for each method:

```
> plotPRMethods <- function(networkSize, sampleCountID) {
+     agg <- getAgg(networkSize)
+     nSamples <- length(sampleCounts)
+     nMethods <- (dim(agg)[2] - 3)/nSamples
+     methodTitles <- array(dim = nMethods)
+     plottedMethods <- array(dim = nMethods)
+     isAllNA <- function(x) {
+         all(is.na(x))
+     }
+     legendY <- 0
+     for (method in 1:nMethods) {
+         methodTitles[method] <- strsplit(colnames(agg)[3 + (nMethods *
+             (sampleCountID - 1)) + method], ".", fixed = TRUE)[[1]][1]
+         if (isAllNA(agg[, 3 + (nMethods * (sampleCountID - 1)) +
+             method])) {
+             plottedMethods[method] <- FALSE
+         }
+         else {
```

```
+             plottedMethods[method] <- TRUE
+         }
+         line <- calcPRLine(truth = agg[, 3], predicted = agg[,
+             3 + (nMethods * (sampleCountID - 1)) + method])
+         if (method == 1) {
+             xLabel <- "Recall"
+             yLabel <- "Precision"
+             plot(line$x, line$y, col = method, pch = method,
+                 type = "l", ylab = yLabel, xlab = xLabel, main = paste("Precision Recall Curve on the\
+                   outputSizes[networkSize], "with", sampleCounts[sampleCountID],
+                   "samples"), xlim = c(0, 1), ylim = c(0, 1),
+                 lwd = 2)
+         }
+         else {
+             lines(line$x, line$y, col = method, pch = method,
+                 lty = method, lwd = 2)
+         }
+     }
+     legend(0, 0.4, methodTitles[plottedMethods], col = which(plottedMethods),
+         pch = "", lty = which(plottedMethods), bty = "n")
+ }
```

And we'll plot the relevant network-sample combination

```
> par(mfrow = c(1, 1))
> plotPRMethods(5, 5)
```

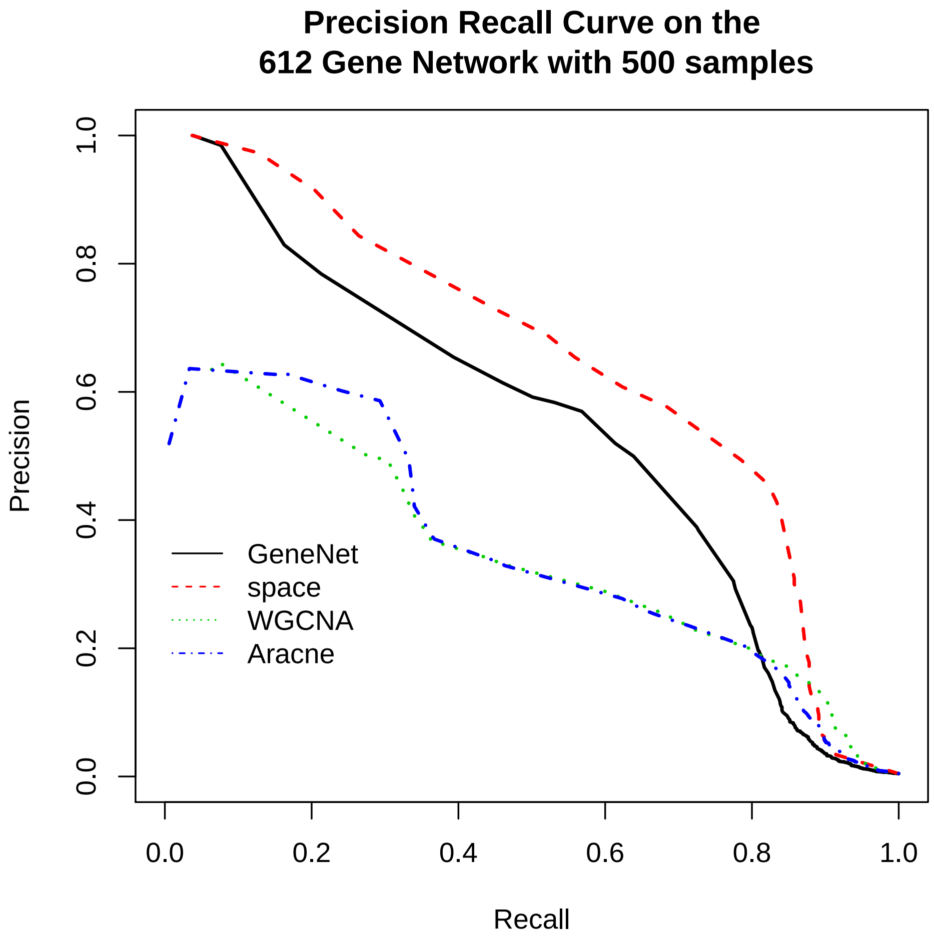


```
> par(mfrow = c(1, 1))
> plotPRMethods(1, 5)
```

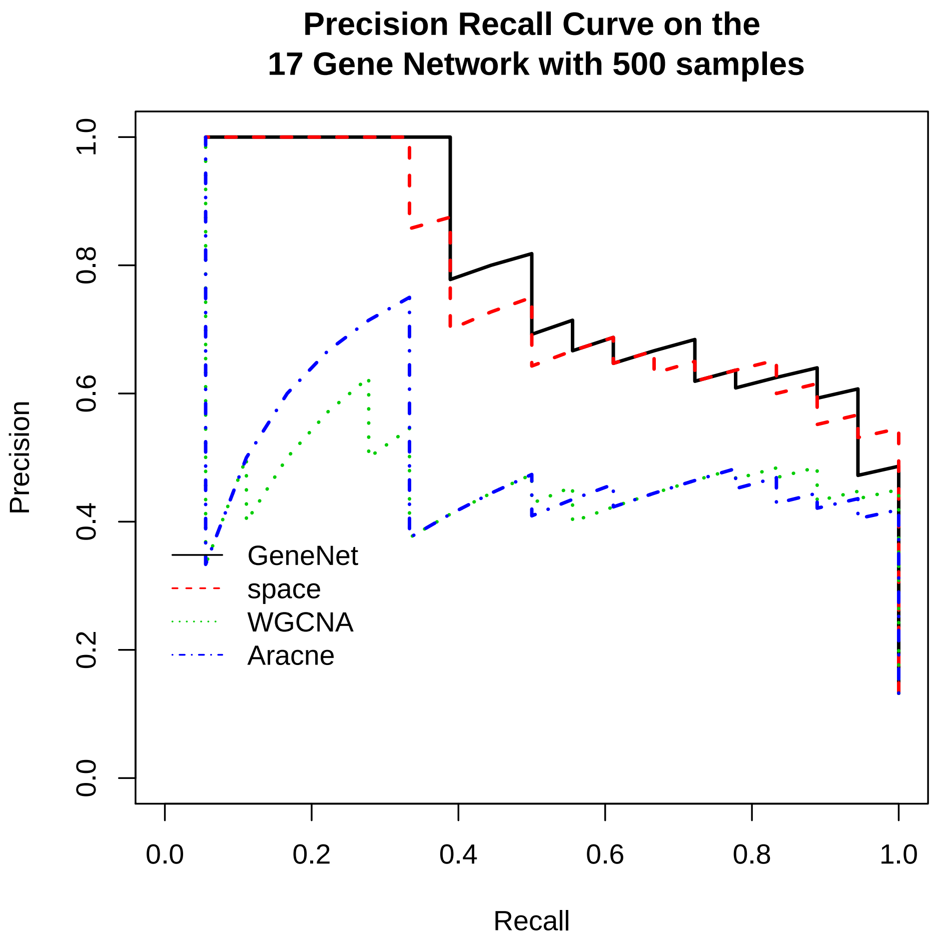
Precision Recall Curve on the
17 Gene Network with 500 samples
Precision
Recall
0.0
0.2
0.4
0.6
0.8
1.0
GeneNet
space
WGCNA
Aracne
